# Signal transduction controls heterogeneous NF-κB dynamics and target gene expression through cytokine-specific refractory states

**DOI:** 10.1038/ncomms12057

**Published:** 2016-07-06

**Authors:** Antony Adamson, Christopher Boddington, Polly Downton, William Rowe, James Bagnall, Connie Lam, Apolinar Maya-Mendoza, Lorraine Schmidt, Claire V. Harper, David G. Spiller, David A. Rand, Dean A. Jackson, Michael R. H. White, Pawel Paszek

**Affiliations:** 1 Systems Microscopy Centre, Faculty of Life Sciences, University of Manchester, Oxford Road, Manchester M13 9PT, UK; 2 The Danish Cancer Society Research Center, Strandboulevarden 49, DK-2100 Copenhagen, Denmark; 3 Warwick Systems Biology and Mathematics Institute, University of Warwick, Coventry CV4 7AL, UK

## Abstract

Cells respond dynamically to pulsatile cytokine stimulation. Here we report that single, or well-spaced pulses of TNFα (>100 min apart) give a high probability of NF-κB activation. However, fewer cells respond to shorter pulse intervals (<100 min) suggesting a heterogeneous refractory state. This refractory state is established in the signal transduction network downstream of TNFR and upstream of IKK, and depends on the level of the NF-κB system negative feedback protein A20. If a second pulse within the refractory phase is IL-1β instead of TNFα, all of the cells respond. This suggests a mechanism by which two cytokines can synergistically activate an inflammatory response. Gene expression analyses show strong correlation between the cellular dynamic response and NF-κB-dependent target gene activation. These data suggest that refractory states in the NF-κB system constitute an inherent design motif of the inflammatory response and we suggest that this may avoid harmful homogenous cellular activation.

## Abstract (web summary)

In biological systems, timing is often critical to the interpretation of signals that determine cell fate. Here the authors demonstrate how single cells and cellular populations respond dynamically to pulsatile stimulation by TNFα and IL-1β, and suggest a mechanism by which the two cytokines can synergistically modulate inflammation.

## Graphical abstract

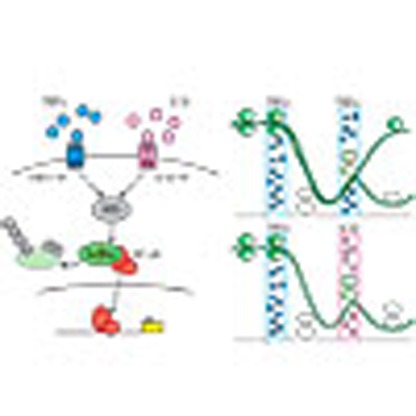

In biological systems, timing is critical in the precise order of events required to produce a functional signalling molecule, to the accurate interpretation of temporally encoded signals that determine cell fate. Cellular fate decisions may vary from fully committed binary outcomes, for example, live or die^1^, to graded responses that are fine-tuned according to the changing amplitude, duration and intensity of the signal^2^. Surprisingly, growing evidence suggests these responses might in fact be random and subject to changes over time^3^. This has been attributed to intrinsic noise in gene expression^4^, heterogeneous dynamics of key transcriptional networks^5^ as well as the existence of multiple cellular states in genetically identical populations^6^,^7^. Cells must reproducibly discriminate varying environmental signals over time; however, how these apparently heterogeneous responses may be coordinated in single cells and cellular populations is not fully understood.

The nuclear factor kappa B (NF-κB) transcription factor is among the best characterized mammalian signalling systems involved in an immune response^8^, and its deregulation is associated with inflammatory disease and cancer^9^. NF-κB p65 exhibits heterogeneous nuclear-to-cytoplasmic oscillations in its cellular localization in response to tumour necrosis factor α (TNFα)^10^,^11^,^12^,^13^, a principal inflammatory signalling molecule. These dynamics are in part due to NF-κB-dependent transcription of inhibitory kappa B protein family (mainly IκBα and IκBɛ), which regulate intracellular localization of the NF-κB (refs 10, 14). Changes in oscillation frequency were associated in part with differential gene expression^15^, suggesting that the NF-κB system, like calcium Ca^2+^ (ref. 16) and other biological oscillators^5^, may be capable of decoding extracellular signals by frequency. The activation of the NF-κB system is also encoded digitally, as the decrease of the TNFα concentration over four orders of magnitude (or the level of antigen stimulation in lymphocytes^17^) resulted in fewer responding cells in the population^2^,^18^. Additional analogue parameters, including the amplitude of NF-κB nuclear translocation, among others, also contributed to the downstream gene expression patterns^2^,^15^,^19^. A long-term pulsed cytokine input resulted in more synchronous NF-κB translocations and increased downstream gene expression, compared with a continuous treatment, suggesting that the NF-κB system may be capable of encoding rapidly changing environmental signals^20^.

The regulation of the IκB kinase (IKK) has been proposed to be particularly relevant for the temporal control of NF-κB responses^21^. IKK integrates different signals ranging from stress, bacterial endotoxin or cytokine stimulation, such as TNFα and interleukin 1β (IL-1β)^22^,^23^. Stimulus-dependent activation of IKK, a multi-protein complex composed of IKKα, IKKβ and a catalytic subunit NEMO, leads to degradation of IκB inhibitors and release of NF-κB into the nucleus^8^. IKK activity is temporally controlled via conformational and phosphorylation cycles^24^, which are dictated by a range of mechanisms. These involve a network of complex and not fully resolved interactions including over 20 molecular species, for example, TRAFs and RNF11 adaptors, RIP and TAK1 kinases as well as IRAK1-4, TAK1, Lubac, Cezanne, ABIN, Tpl2 and Itch among others^8^,^25^,^26^. These proteins play a key role in transduction of different signals; for example, TNFα and IL-1β act via their cognate receptors and have been shown to converge on IKK via TRAF2/RIP and TRAF6/IRAK-mediated pathways, respectively^27^,^28^,^29^,^30^. The responsiveness of cells may be limited by their ability to regulate IKK via A20 (a dual-function deubiquitinase and E3 ligase enzyme) in a negative NF-κB-dependent transcriptional feedback loop^15^,^31^. Indeed, A20-deficient cells are unable to resolve NF-κB response due to disruption of ubiquitin-controlled IKK activity^29^,^32^.

During inflammation, cells are often exposed to a rapidly changing tissue-level environment. For example, TNFα may be produced by a number of immune cells, at different times and doses, creating steep local concentration gradients over time^33^,^34^,^35^. IL-1β may be released in bursts associated with inflammasome-mediated activation of myeloid cells^36^. In this work, we investigated how dynamic cytokine signals are encoded by the NF-κB system in individual cells. We employ time-lapse microscopy to track p65 and IκBα fluorescent fusions in human S-type neuroblastoma (SK-N-AS) cells in response to pulses of TNFα and 1L-1β at different time intervals ranging from 50 to 100 min. We discovered that the observed single-cell NF-κB dynamics are controlled by a stimulus-specific refractory state, which varied between cells, resulting in cellular heterogeneity. The refractory state is not stochastic, because it was conserved in individual cells for several hours. Mathematical modelling predicts that it was due to pseudo-stable cellular states or levels of protein in the signal transduction pathway. We hypothesize that refractory states might be a part of an inherent design motif in the inflammatory response, which enables precise control of the tissue-level inflammatory response by the timing of cytokine stimulation and cellular heterogeneity.

## Results

### NF-κB p65 and IκBα oscillate out-of-phase in single cells

**Figure 1:**
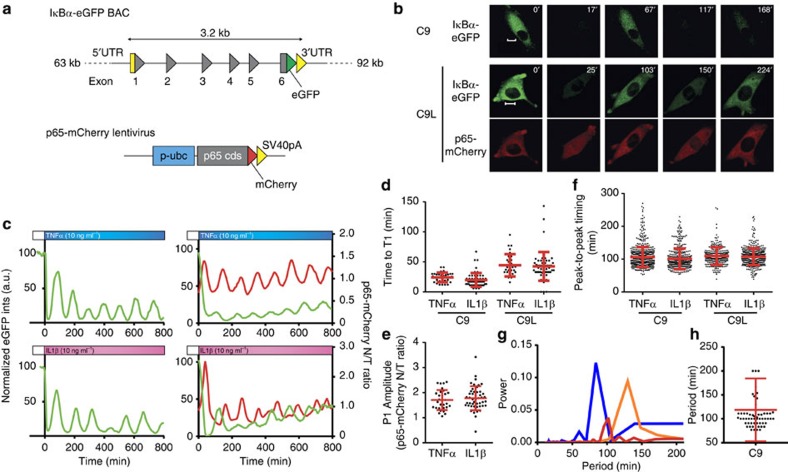
IκBα levels oscillate out-of-phase with NF-κB p65 nuclear localization in response to TNFα and IL-1β stimulation. (**a**) Schematics of IκBα-eGFP BAC and NF-κB p65-mCherry lentiviral constructs. (**b**) IκBα and NF-κB oscillations in response to continuous TNFα. Shown are confocal microscopy images of representative C9 (top panel) and C9L (bottom panel) single cells. IκBα-eGFP and p65-mCherry signal depicted in green and red colours, respectively. Time from stimulation depicted in minutes. Scale bar, 10 μm. (**c**) Representative traces of C9 (left panel) and C9L (right panel) cells stimulated with continuous TNFα or IL-1β, respectively. Shown are the normalized total IκBα-eGFP intensity (in green, with respect to 0 min) and the mean p65-mCherry nuclear to total (N/T) fluorescence ratio (in red). Time depicted in minutes. (**d**) Time to NF-κB activation in response to TNFα and IL-1β stimulation, respectively. Time to activation (T1) defined as the first trough of the total IκBα-GFP signal. Shown are individual C9 and C9L cell data (depicted with dots), with corresponding means (±s.d.), per condition (based on single-cell trajectories as in **c**). (**e**) The amplitude of the first NF-κB p65 translocation. The amplitude (P1) defined as the peak p65-mCherry N/T ratio of the first NF-κB nuclear translocation. Shown are individual C9 and C9L cell data, with corresponding means (±s.d.), per condition (based on single-cell trajectories as in **c**). (**f**) Timing of TNFα- and IL-1β-stimulated NF-κB oscillations. Shown are individual peak-to-peak timings for C9 and C9L cells, respectively, with corresponding mean (±s.d.) per condition. Peak-to-peak defined as the time between consecutive troughs in the total IκBα-eGFP signal. (**g**) Power spectrum analysis of the NF-κB oscillation period. Shown are power spectra calculated for three representative C9 cells (depicted with different colour) in response to TNFα based on the IκBα-eGFP trajectories (for times >35 min, Fig. 5). Peak in the power spectra indicates a dominant period in a cell. (**h**) Period of NF-κB oscillations in single cells. Shown are individual cell oscillation periods identified based on the power spectrum analysis as in **g**, for C9 cells stimulated with TNFα (data from **f**).

To investigate cellular responses to temporal cytokine input, we visualized NF-κB system dynamics using time-lapse fluorescent microscopy^10^,^15^,^18^. We produced SK-N-AS cells (referred to here as C9 cells) stably expressing 3′UTR eGFP-tagged full-length human IκBα gene from a bacterial artificial chromosome (BAC) construct (Fig. 1a, see also Supplementary Note 1 for cell line development). Additionally, we also introduced a lentiviral construct expressing a NF-κB p65-mCherry fusion protein^37^ (Fig. 1a, referred to as C9L cells). Resting cells showed a cytoplasmic localization of both IκBα-eGFP and p65-mCherry. Treatment with 10 ng ml^−1^ of TNFα (or IL-1β) led to a rapid and synchronous degradation of IκBα-eGFP, which coincided with a nuclear p65-mCherry translocation (Fig. 1b–d, see Supplementary Table 1 for the number of cells analysed in specific experiments). The corresponding NF-κB p65 translocation amplitudes were very similar for both cytokine treatments (Fig. 1e, represented by a ratio of p65-mCherry nuclear to total intensity with the total concentration of p65 fusion per cell constant in time^10^,^15^,^37^). The timing of degradation and re-synthesis of IκBα-eGFP matched those exhibited by endogenous IκBα after TNFα stimulation (see Supplementary Figs 1 and 2 for immunoblotting analysis).

Over a long-time course (up to 800 min), single cells exhibited persistent oscillations in the level of IκBα-eGFP corresponding to multiple rounds of IκBα re-synthesis and degradation (Fig. 1c, see Supplementary Fig. 3 for the conserved NF-κB dynamics in different model systems). The IκBα-eGFP oscillations were out-of-phase with respect to nuclear translocations of p65-mCherry, as shown by the frequency analysis (Supplementary Fig. 4). In agreement with previous data in SK-N-AS cells^10^,^15^,^18^, NF-κB oscillations had an average period of ∼100 min, but were heterogeneous between cells, as depicted by individual peak-to-peak timings, Fig. 1f. Frequency analysis demonstrated that many individual cells exhibited distinct periods ranging from 80 to 140 min (see Fig. 1g and Supplementary Fig. 5 for power spectrum analysis, Fig. 1h for distribution of single-cell periods). This highlighted that some key aspects of NF-κB responses might be differentially controlled by parameters inherent to individual cells^13^.

### Cells exhibit refractory periods to pulsatile stimulation

**Figure 2:**
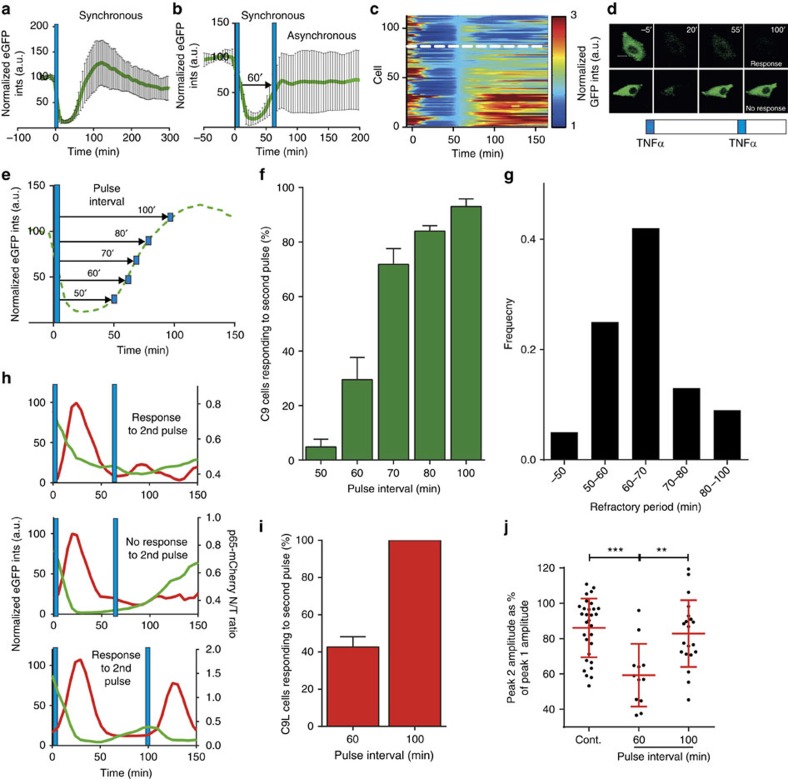
TNFα pulsing reveals a heterogeneous refractory period in the NF-κB system. (**a**) Response to a single 5-min pulse of TNFα. Shown is the mean (in green) (±s.d.) of the normalized total IκBα-eGFP intensities in single C9 cells. Timing of TNFα stimulation represented with a blue bar. (**b**) Response to two 5 min TNFα pulses applied at 0 and 60 min. Shown is the mean (±s.d.) of the normalized total IκBα-eGFP intensity in single C9 cells. Timing of TNFα stimulation represented with blue bars. (**c**) Clustering analysis of single-cell data from **b** with respect to normalized total IκBα-eGFP single-cell intensities at the time of the second pulse. Broken line indicates responding (top) and non-responding (bottom) clusters. (**d**) Confocal images of representative non-responding (top) and responding (bottom) cells from **c**. The IκBα-eGFP signal shown in green colour. Scale bar, 10 μm. (**e**) Schematic representation of repeat pulsing: Two 5 min TNFα pulses applied at time intervals ranging from 50 to 100 min. Green dotted line represents a putative IκBα-GFP response to the first TNFα pulse. (**f**) Fraction of C9 cells responding at different TNFα pulse intervals. Single-cell responsiveness (means ±data range of at least two replicates) based on clustering analysis of normalized single-cell total IκBα-eGFP intensities (as in **c**). (**g**) Distribution of refractory periods based on data in **f**. (**h**) Representative C9L cells stimulated with TNFα pulses at 60 and 100 min intervals (as depicted with blue bars). Shown are normalized total IκBα-eGFP intensities (in green) and the nuclear to total (N/T) ratio of the p65-mCherry signal (in red). (**i**) Fraction of responding C9L cells at 60 and 100 min pulse interval. Shown is mean±data range per condition. (**j**) Amplitude of the second NF-κB translocation. Shown are C9L cell responses to continuous, as well as 5 min TNFα pulses at 60 and 100 min intervals, respectively, with mean ±s.d. of the second peak (P2) nuclear NF-κB p65-mCherry translocation amplitude, expressed as a fraction of the first-peak amplitude. Statistical difference assessed with a Student's *t*-test (**P value<0.01, ***P value<0.001).

To understand how cells may encode changing inflammatory signals, first, C9 cells were stimulated with a single 5-min pulse of 10 ng ml^−1^ TNFα (by manually changing media on cells). This resulted in a single synchronous degradation of IκBα-eGFP with a trough at 20 min in all cells (as shown by a population average of 63 cells, Fig. 2a, mirroring previously described NF-κB p65 nuclear translocation^15^). Subsequently, IκBα-eGFP levels peaked at 125 min and returned to the pre-stimulation levels between 200 and 300 min. However, when two 5-min pulses of TNFα were applied at a 60 min interval (first pulse at time 0 min followed by a second pulse at 60 min), the responses to a second pulse were more asynchronous in comparison with responses to a first pulse, or single-pulse stimulation (Fig. 2a versus b). Clustering analysis of individual traces showed that while all the cells responded to the first pulse, only ∼30% (out of 113) cells responded to a second pulse of TNFα, based on degradation of IκBα-eGFP (Fig. 2c,d for images of representative cells). In the remaining 70% of cells IκBα-eGFP continued to accumulate after treatment, giving a profile similar to the single-pulse stimulation response (Supplementary Fig. 6). In comparison, when two pulses of IL-1β were applied at a 60-min interval, 75% of cells responded to the second pulse, suggesting that cell's responsiveness is stimulus-specific (Supplementary Fig. 9).

Pulsatile TNFα stimulation was then applied at time intervals ranging from 50 to 100 min (as shown in Fig. 2e). We found that while all the cells responded to the first TNFα pulse, at shorter pulse intervals (<100 min) cells increasingly failed to respond to the second pulse (Fig. 2f, see Supplementary Note 2, Supplementary Fig. 7 for single-cell traces, and Supplementary Fig. 8 for the clustering analysis of ‘responding' and ‘non-responding' cells). At a 100-min pulse interval, 93% of cells responded to both pulses; however, the fraction of responding cells decreased to 70 and 30% at 70 and 60 min pulse intervals, respectively. With a 50-min interval, only 5% of cells showed NF-κB activation to a second pulse. These data suggest the existence of a refractory state induced by the initial stimulation, in which cells become temporally unresponsive to TNFα. The refractory state is heterogeneous, as the time period where cells are unresponsive to the second TNFα pulse (defined here as the refractory period) varied across the population (Fig. 2g, calculated based on the data in Fig. 2f, see Materials and methods section) with ∼40% of cells characterized by a refractory period between 60 and 70 min.

Previous analyses suggested that the timing of TNFα pulsing regulated the amplitude of NF-κB nuclear translocation^15^. In IκBα-eGFP p65-mCherry C9L cells pulsed at 100 min interval (see Fig. 2h for single-cell traces, Fig. 2i for fraction of responding cells), the average amplitude of p65-mCherry nuclear translocation was similar to that of cells stimulated with continuous TNFα (80% of the first-peak amplitude, Fig. 2j). However, when pulsed at 60 min, the nuclear p65-mCherry amplitude was significantly lower and equated to 60% of the first-peak amplitude.

Altogether, these data showed that individual cells exhibited stimulus-specific heterogeneous activation (as exhibited by IκBα-eGFP degradation) in response to pulsatile cytokine stimulation, with fewer cells responding at shorter TNFα pulsing intervals. The amplitude of NF-κB p65 nuclear translocation in cells that responded depended on the length of the pulsing interval, suggesting both digital and analogue signal-encoding elements. These responses were apparently stochastic, with most of the variability being captured by the fraction of responding cells (coefficient of variation of 1.1 at 60 min), rather than the amplitude of activation (coefficient of variation of 0.3 for the peaks 2 to 1 NF-κB p65 nuclear intensity ratio in responding cells).

### Refractory period is encoded in the IKK network

**Figure 3:**
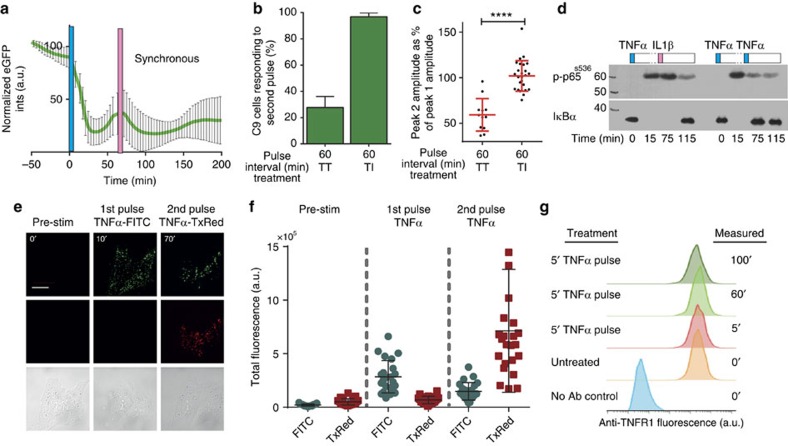
The refractory period is controlled downstream of TNFR and upstream of IKK. (**a**) Response to alternate TNFα and IL-1β pulses applied at 0 and 60 min Shown is the mean (±s.d.) of normalized total IκBα-GFP intensity in single C9 cells. Timing of TNFα (0 min) and IL-1β (60 min) stimulation represented with blue and pink bars, respectively. (**b**) Comparison between responses to two TNFα pulses (TT) and alternate TNFα and IL-1β (TI) pulses at 60 min interval. Shown are fractions (mean ±s.d.) of C9 cells that responded to the second pulse. (**c**) NF-κB amplitude of cells responding to two TNFα pulses (TT) and alternate TNFα and IL-1β (TI) pulses at 60 min interval. Shown are the peak 2 (P2) p65-mCherry translocation amplitudes (expressed as the fraction of the peak 1 amplitude, P1) of individual C9L cells, with corresponding mean and±data range per condition. In TI, TNFα and IL-1β pulses applied at 0 and 60 min, respectively. Statistical difference assessed with a Mann-Whitney test (**** *P* val<0.0001). (**d**) Immunoblotting analysis of IκBα and serine 536-phosphorylated NF-κB p65 levels in WT cells stimulated with two pulses of TNFα, or alternate TNFα and IL-1β pulses at 60 min interval. Timing of TNFα and IL-1β stimulation represented with blue and pink bars, respectively. (**e**) Confocal microscopy images of WT cells stimulated with two pulses of fluorescently labelled TNFα at 60 min interval. FITC-conjugated TNFα (top) was applied at 0 min and measured 10 min after stimulation. Tx-Red-conjugated TNFα (middle) was applied on the same cells at 60 min, and measured at 70 min after the start of the experiment. Corresponding bright field images shown at the bottom. Scale bar, 20 μm. (**f**) TNFα internalization in WT cells as in **e**. Shown is total fluorescence levels per cell measured at 10 min after stimulation at 0 and 60 min, respectively. (**g**) Flow cytometry analysis of TNFR1 receptor expression. WT SK-N-AS cells were stimulated with 5 min pulse of TNFα and TNFR1 expression was measured by flow cytometry at 5, 60 and 100 min after treatment (in addition to untreated and unlabelled controls).

Stimulus-induced activation of IKK is regulated by temporally coordinated conformational and phosphorylation cycles^24^. Existing models suggest that, on stimulation, IKK undergoes a rapid activation and before it can be reactivated must return to the unstimulated state^2^,^14^,^15^,^23^,^28^,^31^. We therefore compared responses to two TNFα pulses (TT) to stimulation with pulses of TNFα and IL-1β (TI). While only 30% of cells responded to two pulses of TNFα at a 60-min interval (Fig. 2f), 95% of cells responded when TNFα-treated cells were stimulated with IL-1β after 60 min (see Fig. 3a for an average of 95 C9 cells and Fig. 3b for the fraction of responding cells). Also, the amplitude of the second NF-κB p65-mCherry nuclear translocation in responding cells was higher when treated with IL-1β rather than TNFα (Fig. 3c) suggesting parallel signal transduction to IKK (refs 27, 28, 29, 30). Immunoblotting for IκBα protein level and serine 536-phosphorylated p65 (ref. 10) in WT SK-N-AS cells, confirmed NF-κB system activation in response to a second pulse of IL-1β, but not TNFα (Fig. 3d). This experiment suggested that the refractory period following TNFα stimulation is controlled upstream of IKK in the TNFα transduction pathway. 54% of cells responded to a TNFα pulse 60 min after a pulse of IL-1β (Supplementary Fig. 9), indicative of some cross-talk between IL-1β and TNFα signal transduction pathways.

We then investigated whether the TNFα refractoriness depended on the receptor availability^2^. First, cells were stimulated with 5 min pulses of fluorescein isothiocyanate (FITC) or Tx-Red fluorescently labelled TNFα at 60 min intervals. In agreement with previous reports^38^, a 5-min pulse produced rapid cell surface binding and internalization of FITC-labelled TNFα (see Fig. 3e and Supplementary Fig. 10 for representative cells and Fig. 3f for population level). When re-stimulated after 60 min with Tx-Red-labelled TNFα, all cells again exhibited rapid binding and internalization of the labelled cytokine indicative of TNFα receptor expression (Fig. 3e,f, and Supplementary Note 3 and Supplementary Figs 11 and 12 with reverse labelling). Importantly, when the availability of the tumour necrosis factor receptor-1 (TNFR1), a cognate TNFα receptor^38^, was measured directly by flow cytometry, no change of cell surface expression was observed between different pulsing protocols (in comparison to untreated cells, Fig. 3g). This suggests a model where the TNFR1 cell surface availability is constantly maintained via efficient trafficking to the cell membrane^39^, with the NF-κB response being independent of endocytosis (Supplementary Fig. 13). Overall, these data show that the TNFα signal was decoded in a transduction pathway upstream of the IKK but downstream the TNFα receptor.

### Signal transduction controls single-cell responses

**Figure 4:**
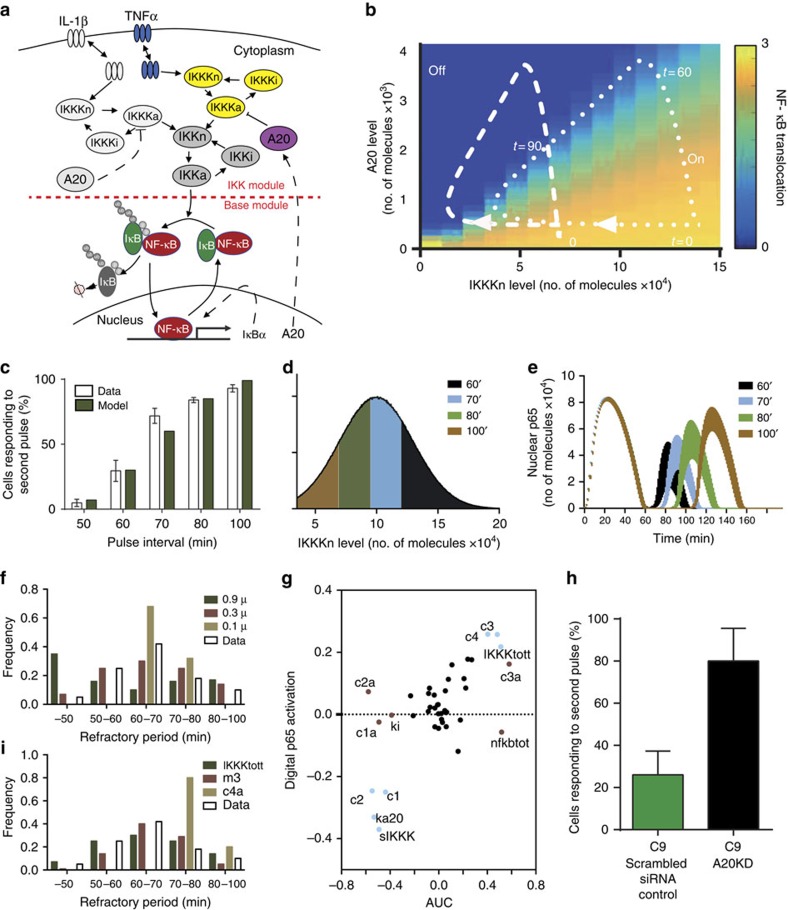
Mathematical model recapitulates single-cell responses. (**a**) Schematic representation of the IKK signalling module. (**b**) IKKK and A20 levels regulate NF-κB response. Cells stimulated with two 5-min TNFα pulses at 70 min interval. Shown is the nuclear NF-κB level in response to a second TNFα pulse, stratified into ‘responsive' (yellow) and ‘non-responsive' (blue) regimes (defined by a normalized net NF-κB translocation), simulated for different A20 and IKKKn levels. Two single-cell trajectories (for different levels of total IKKK, see Supplementary Table 7) in response to a 5-min pulse shown with dashed lines. Refractory periods indicated with time stamps. (**c**) Fraction of cells responding at different pulse intervals. Model simulations (300 cells per condition, in black) versus data (from Fig. 2f). (**d**) Fitted IKKK level distribution. Quantiles define fractions of responding cells at different pulsing intervals. (**e**) Nuclear NF-κB amplitude in responding cells. Shown is the range between single-cell trajectories corresponding to the minimum value of each quantile and the maximum IKKK level as in **d**. (**f**) Refractory period distribution as a function of the s.d. of IKKK distribution (*σ*). Simulations (300 cells) for *σ* equal to 0.9 *μ*, 0.3 *μ* and 0.1 *μ*, where *μ*=10^6^ is mean IKKK level. Shown also, is the measured distribution from Fig. 2g. (**g**) Global sensitivity analysis of NF-κB system response to 70 min TNFα pulse stimulation. Shown is the correlation between sensitivity scores for fraction of responding cells (defined by a net NF-κB nuclear translocation) and area under the curve (AUC) of nuclear NF-κB trajectory in response to the second pulse. Shown in blue are parameters controlling the NF-κB amplitude and AUC, in brown parameters controlling only AUC. (**h**) A20 regulates refractory period. Shown is the fraction of cells treated with A20 siRNA (or scrambled control) responding to the second pulse (mean±data range). C9 cells stimulated with two 5 min TNFα pulses applied at 0 and 60 min. (**i**) Refractory periods simulated with distributed total IKKK level (IKKKtott, *μ*=nominal parameter value), IKKK recovery rate (m3, *μ*=1.2 × nominal value), IκBα protein half-life (c4a, *μ*=nominal value), versus data (Fig. 2g). s.d. of the normal distribution set to *σ=*0.3*μ* for respective parameters (300 simulated cells per condition).

To theoretically investigate the encoding of pulsed TNFα stimulation in the NF-κB system, the structure^2^ and cell-system specific parameters^14^,^15^,^18^ of previously developed models were considered (see Supplementary Note 5 for model development and validation, and Supplementary Figs 14–20; refs 21, 28, 32, 40). The mathematical model comprised two interconnected modules (Fig. 4a). The base module described the interaction between NF-κB and its IκBα negative feedback^15^, whilst the IKK module depicted the transduction pathway downstream of TNFα and IL-1β. In particular, the model incorporated an upstream generic IKK kinase (IKKK) and A20 feedback protein, which together regulate IKK activity^29^,^32^,^41^. In this description, IKKK represents a large network of complex and not fully characterized molecular interactions (for example, TRAF adaptors, RIP and TAK1 kinases)^8^,^30^, while the A20 also includes other A20-like feedbacks (for example, cIAP or Cezanne)^42^ and proteins that control their activity (for example, ABIN, Lubac and TPL2)^25^,^26^,^29^. Based on the data in Fig. 3 and Supplementary Fig 9, we assumed that the TNFα and IL-1β signals converge on the IKK via signal-specific IKKK kinases (see Supplementary Fig. 16 for the model fit to TNFα and IL-1β pulsing)^27^. The total level of IKKK was assumed to be constant per cell^2^,^14^, while A20 limited IKK activity by inhibition of the ‘active' IKKK.

The generic IKKK and A20 (but not IκBα, Supplementary Fig. 21) levels effectively divided the solution space into ‘responding' and ‘non-responding' regimes (Fig. 4b). Typical unstimulated cells are located in a ‘responding' regime, characterized by a high ‘neutral' IKKK (IKKKn, a form that could be activated by a cytokine signal) and low A20 protein levels (as depicted at *t=0* for two representative cell trajectories, shown with broken lines). A pulse of TNFα leads to a rapid decrease of IKKKn (converted to its ‘active' form), subsequent IKK activation, NF-κB translocation and A20 synthesis. This coincided with single-cell trajectories crossing over to the ‘non-responding' regime, characterized by a low IKKKn and high A20, where they become refractory to a second pulse of TNFα (Fig. 4b). Over time equivalent to the refractory period, cells returned into the ‘responsive' regime, as the IKKKn levels recovered from the ‘inactive' state, while the A20 protein degraded.

The distribution of refractory period was recapitulated in the model by IKKK levels normally distributed in the population but fixed per cell (Fig. 4c,d, however, other distributions could also fit the data, Supplementary Fig. 22). For a fixed level of A20 protein, higher IKKK levels resulted in faster IKKKn recovery thus enabling subsequent activation (Supplementary Fig. 15). Therefore, when pulsed at longer intervals, responding cells exhibited higher amplitude of NF-κB p65 translocation as observed in the data (Fig. 4e). We found that changes in parameters of the IKKK distribution directly affected the mode and shape of the refractory period distribution. Increasing the mean IKKK level resulted in a shorter refractory period on average (Supplementary Fig. 23), while increasing the s.d. resulted in a more uniform distribution, with 40% of cells responding at the 50-min pulse interval (Fig. 4f).

We next used sensitivity analyses to understand which biochemical parameters may control the NF-κB responses. Latin hypercube sampling^43^ (and dynamic sensitivity analyses^44^, Supplementary Figs 26 and 27) showed that cell responsiveness to TNFα pulses (as defined by amplitude of NF-κB translocation) was controlled by parameters associated with the IKKK and A20 signalling (for example, A20 mRNA transcription, protein translation rates and half-lives, *c1*, *c2*, *c3*, *c4*; IKKK level, *IKKKtott*; A20-induced IKKK inhibition, *kA20*; half-maximal IKK activation, *sIKKK*, Fig. 4g shown in blue), but not the core NF-κB-IκBα or receptor signalling (see also Supplementary Fig. 28). In contrast, IκBα- and IKK-related parameters (transcription and translation rates, *c1a*, *c2a*; mRNA half-life, *c3a*; total NF-κB level, *nfkbtot*; and IKK inactivation rate, *k1*, Fig. 4g shown in brown) contributed (together with IKKK- and A20-related parameters) to the cumulative amount of nuclear NF-κB. The IκBα-related parameters also controlled the period of NF-κB oscillations, while A20- and IKKK-related parameters contributed to their amplitude (Supplementary Fig. 24). In agreement with the hypothesis that the depletion (or inhibition) of IKKK regulates the refractory period, all cells responded to a 10 ng ml^−1^ pulse 60 min after a low dose 0.1 ng ml^−1^ TNFα pulse. The mathematical model suggested that despite induced A20 protein levels in cells that responded to the first pulse, simulated IKKKn levels were sufficiently high to enable subsequent NF-κB activation, Supplementary Fig. 18a. Additionally, only 40% of cells responded to a 50 ng ml^−1^ pulse when the refractory period was set with a 10 ng ml^−1^ dose (in comparison to 30% of cells responding to two 10 ng ml^−1^ pulses), Supplementary Fig. 18b. This suggested that refractory state could not be efficiently reversed with a high-dose stimulation, supporting mathematical model predictions. Finally, when short interfering RNA (siRNA) was used to reduce A20 expression, a substantial increase in cells responding to TNFα pulses was observed (80% versus 25% scrambled siRNA control, Fig. 4h). In agreement, distributed individual parameters associated with A20/IKKK signalling (such as IKKKn recovery rate, *m3*, or total IKKK, *IKKKt*), but not IκBα (for example, protein half-life, *c4a*) were able to fit the measured refractory period distribution (Fig. 4i). This together showed the critical role of the TNFα signal transduction and A20 protein in the regulation of the refractory period.

### Heterogeneous single-cell responses are imprinted

**Figure 5:**
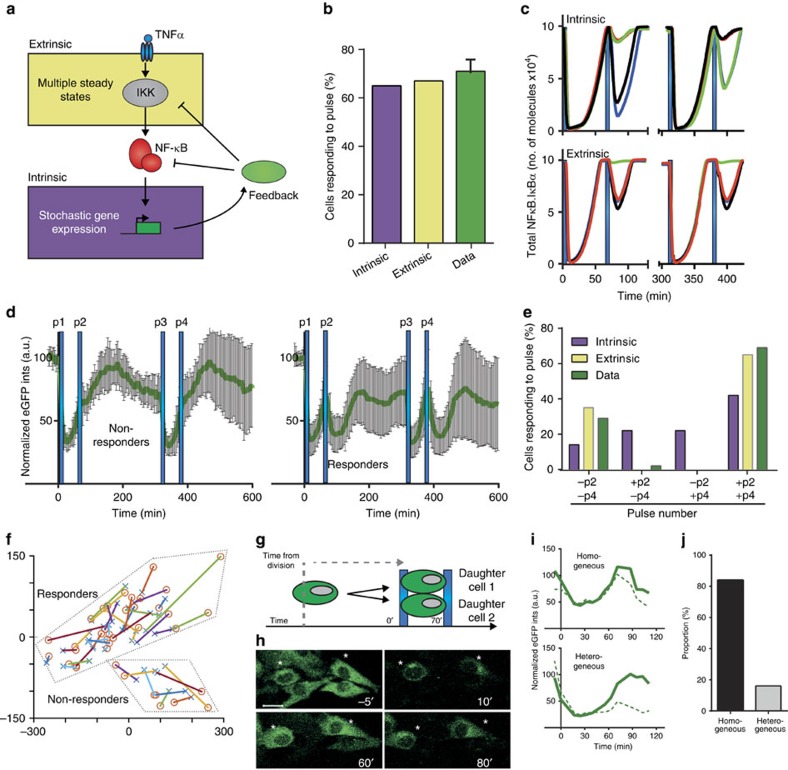
Single-cell responses to pulsed TNFα stimulation are imprinted. (**a**) Schematic representation of the intrinsic (blue) and extrinsic (yellow) noise in the NF-κB system. (**b**) Different noise models fit single-cell data. Shown is the comparison between simulation (300 cells) of extrinsic noise (via the distributed *A20* transcription rate) and intrinsic noise (via the stochastic regulation of *A20* gene activity) model, and the data for TNFα pulses at 70 min interval. (**c**) Simulated single-cell traces (shown as NF-κB:IκBα complex levels, in different coloured lines) for different noise models. TNFα applied at two 70 min intervals separated by 4 h equilibration phase (as depicted with blue bars). (**d**) C9 cell responses to equilibrated TNFα pulses (as in **c**). Shown are means (in green, ±s.d.) of normalized single-cell total IκBα-GFP intensities in nine non-responsive (left panel) and 32 responsive cells (right panel) to second (p2) and fourth (p4) pulse. (**e**) Comparison between model simulations and the data for equilibrated TNFα pulses. Simulation performed with 300 cells assuming intrinsic (in yellow) and extrinsic (in blue) noise (as in **c**). Data from **d** presented in fraction of cells (total 79) responding to second (+p2,−p4), fourth (−p2,+p4) or both second and fourth (+p2,+p4) pulses. (**f**) Principal component analysis of single-cell data from **d**. For each cell, 140 min sub-trajectories corresponding to two stimulation phases were considered (depicted with symbols connected with different colour lines). Responsive and non-responsive cell clusters outlined with dashed lines. (**g**) Daughter cell analysis in response to two TNFα pulses at 70 min interval. Time from cell division to stimulation recorded. (**h**) Representative daughter C9 cell responses (as in **g**): Cells (indicated with stars, IκBα-eGFP intensities shown) respond to the first (depicted at 10 min after stimulation), as well as to the second TNFα pulse (at 80 min after start of the experiment). Scale bar, 20 μm. (**i**) Representative daughter cells trajectories (from the experiment in **g**). (**j**) Fractions of homogenous and heterogeneous daughter cells responses. 56 pairs stimulated as in **g**, stratified by patterns of the IκBα-eGFP signal.

The mathematical model (Fig. 4) suggests a paradigm where heterogeneous responses to pulsatile TNFα stimulation are generated by ‘extrinsic' differences between cells (modelled by distributed network parameters). In contrast, an alternative hypothesis suggests that this behaviour might arise from ‘intrinsic' noise due to low numbers of interacting molecules, for example gene copies^4^ (Fig. 5a for schematic representation of noise in the NF-κB system). A model assuming a stochastic regulation of the A20 feedback, but a fixed level of IKKK, could explain digital NF-κB activation (Fig. 5b) and would be consistent with previous analyses^14^,^20^,^45^. To distinguish between these alternatives we stimulated cells with two pairs of TNFα pulses at 70 min intervals separated by a 4 h equilibration period (Supplementary Fig. 29). Model simulations predicted that under the ‘extrinsic' noise hypothesis individual cells would show reproducible NF-κB activation when re-stimulated (Fig. 5c). However, under the ‘intrinsic' noise hypothesis, the responses would be stochastic, that is, a cell that responded to a first set of pulses might not respond to the second pulsing protocol. We tested this prediction experimentally and found that the ‘extrinsic' noise hypothesis was supported (*P* value<0.00001, for the Fisher's exact test between obtained distributions); among 79 C9 cells (78% responding) only three cells behaved differently after the equilibration period (Fig. 5d,e, see also Supplementary Movie 1 and Fig. 5f for principal component analysis of IκBα-eGFP trajectories).

Altogether, these data suggested that NF-κB responses to pulsed stimulation (and thus refractory periods) were innate to individual cells and represented a non-genetic state. To test the stability of this state, the responses of daughter cells stimulated with two pulses of TNFα at a 70 min interval were compared (Fig. 5g). We found that ∼85% of daughter cells (out of 56 pairs) exhibited homogenous responses as depicted by single-cell IκBα-eGFP traces (Fig. 5h–j and Supplementary Fig. 30). In addition, no correlation between the behaviour of daughter cells and the time from cell division at stimulation was found (Supplementary Fig. 31). These data therefore suggested that the refractory states associated with NF-κB system were subject to change at a timescale longer than the cell cycle.

### Refractory states integrate different cytokine inputs

**Figure 6:**
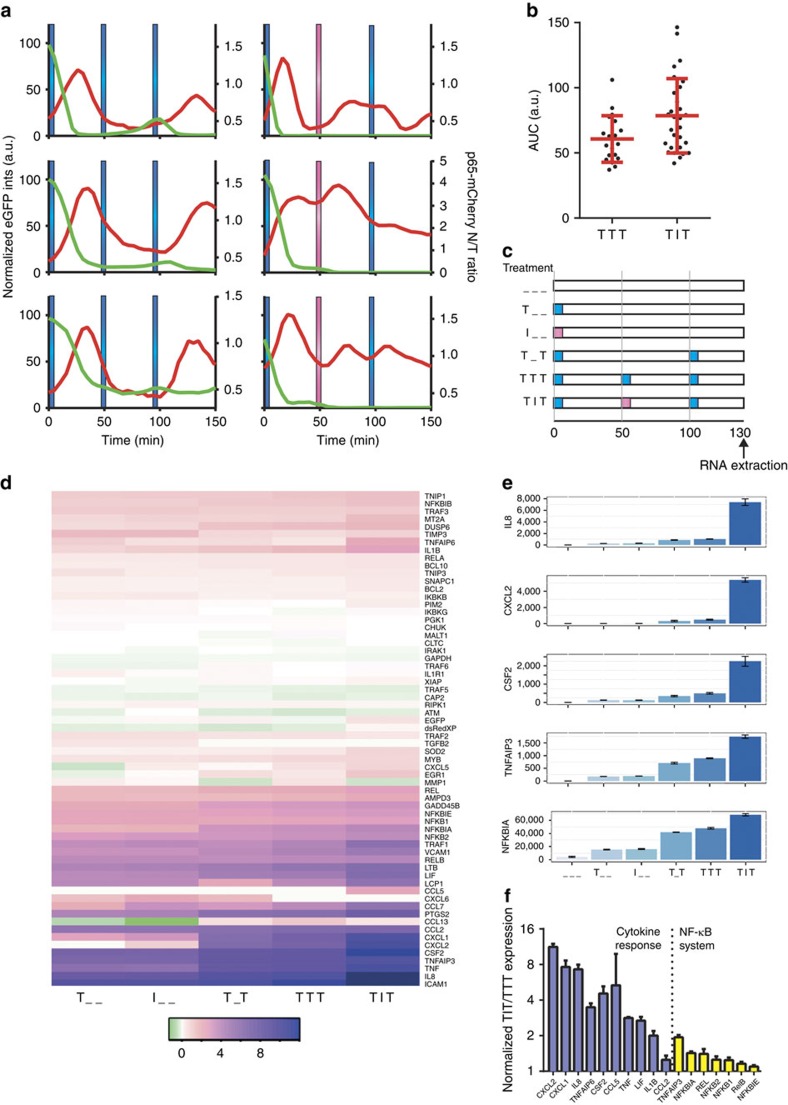
Cells refractory to TNFα encode IL-1β signals. (**a**) Representative single C9L cell traces stimulated with three pulses of TNFα (TTT, depicted with blue bars) or alternate TNFα and IL-1β pulses (TIT, depicted with pink bars) at 50 min intervals. Shown are normalized total IκBα-eGFP fluorescent intensities and the nuclear to total (N/T) ratios of the p65-mCherry signal. Time depicted in minutes. (**b**) Nuclear NF-κB activity in cells stimulated with three pulses of TNFα (TTT) or alternate TNFα and IL-1β pulses (TIT) at 50 min interval. Shown is the area under the N/T p65-mCherry trajectory (AUC) for cells as in **a** (base-line corrected trajectories were normalized to the first peak amplitude). (**c**) Schematic representation of the gene expression assay. Cells were stimulated with pulses of TNFα and IL-1β at different times (as indicated with blue and pink bars, respectively). Measurement obtained at 130 min after start of the experiment. (**d**) Heat map of gene expression levels for data from **c**. Clustering performed for log2 fold changes (as indicated with the colour scale) of three replicates. (**e**) Gene expression levels for *IκBα*, *A20*, *CSF2*, *CXCL2* and *IL8* transcript levels from data in **d**. Shown are mean expression levels (±s.d.) per condition, respectively. (**f**) Differential gene expression analysis between TNFα/IL-1β/TNFα (TIT) and TNFα/TNFα/TNFα (TTT) stimulation. Shown are log2 of expression fold changes for NF-κB system genes (*A20*, *IκBα*, *IκBɛ*, *Rel*, *RelB, NF-κB1* and *NF-κB2*) and cytokine response genes (*CXCL2*, *CXCL1*, *IL8*, *TNFAIP6*, *CSF2*, *TNFα*, *LIF*, *IL-1β* and *CCL2*).

Cells are often exposed to complex cytokine inputs. When stimulated with three pulses of TNFα (TTT) at 50 min intervals, most single cells were refractory to the second pulse, but responded to the third pulse (Fig. 6a). In contrast, stimulation with IL-1β in the second pulse instead of TNFα (TIT) induced a nuclear p65-mCherry translocation in 80% of cells (Supplementary Fig. 32). Although these responses were apparently noisy, responses to TIT stimulation were elevated in comparison to TTT (Fig. 6b), especially when stratified into time intervals between pulses (Supplementary Fig. 34a). This confirms that while refractory to TNFα, other cytokine inputs, for example IL-1β, can be processed by cells. To test if refractory states might enable functional discrimination between different cytokine inputs, we assayed expression of 70 TNFα and IL-1β-regulated genes^46^,^47^ with a Nanostring nCounter system^48^. Cells were stimulated with alternate 50 min TNFα and IL-1β pulses (TT, TIT and T_T), with additional single pulse and untreated controls (Fig. 6c). The comparison between the stimulation with two (T_T) and three (TTT) pulses of TNFα showed only eight differentially regulated genes (out of 62 expressed genes; including 18 constitutively expressed in addition to 5 housekeeping genes, see Supplementary Data 1 for details of the Nanostring analysis including raw and normalized data, and pair-wise comparisons between conditions). This confirmed that the cells were functionally refractory to the second pulse of TNFα at 50 min, in close agreement with imaging data (Fig. 6a,b). Differentially regulated genes included members of the NF-κB system, *NF-κB1*, *NF-κB2* and *IκBɛ*; signalling molecules, *CCL2* and *CSF2*; and A20-interacting *TRAF1* and *TNIP1* (ref. 27). The change in expression level compared with unstimulated control samples was relatively low and did not exceed 50%. In contrast, the comparison between TIT and TTT stimulation, showed 26 genes that were differentially regulated by IL-1β (Supplementary Fig. 33). This set included members of the NF-κB signalling system (*Rel*, *RelB*, *NF-κB1*, *NF-κB2*, *IκBα*, *IκBɛ* and *A20*) and a number of pro-inflammatory signalling molecules including cytokines and chemokines (*IL8*, *CSF2*, *CCL2*, *CCL5*, *CXCL1*, *CXCL2*, *LIF*, *TNFα, TNFAIP6* and *IL-1β*). The first group showed only modest increase (<50%) in response to TIT compared with TTT stimulation (with *A20* and *IκBα* highest with two-fold and 1.4-fold change, respectively). Notably, all of the signalling molecules exhibited changes over two-fold with *CCL1*, *CXCL2*, *IL8* and *TNFAIP6* higher than five-fold (Fig. 6f). In contrast, comparison between single TNFα and IL-1β pulses did not show a difference in the expression of those genes (see Supplementary Fig. 34e and Supplementary Data 1). Integrated single-cell nuclear NF-κB levels were very well correlated with the measured gene expression patterns across different pulsing conditions (Supplementary Fig. 34). In particular, we found the expression of all differentially regulated genes (including NF-κB-system and cytokine genes, Fig. 6f) showed a very high correlation (close to 1) irrespectively of their response amplitude. This analysis suggests that refractory states might enable cells to functionally discriminate between different closely timed cytokine inputs.

## Discussion

In this study, we investigated how rapidly changing cytokine inputs are encoded in the dynamics of the NF-κB signalling system. We employed time-lapse microscopy to quantitatively measure activation of the NF-κB p65 and its negative feedback IκBα in response to a pair of 5 min pulses of TNFα (and IL-1β), which were applied at different time intervals, ranging from 50 to 100 min. Single, or well-spaced pulses of TNFα (>100 min apart) gave a high probability of NF-κB activation. However, we found that at shorter pulse intervals (<100 min) responses were heterogeneous, with progressively more cells failing to respond to the second pulse (Fig. 2). This identified a heterogeneous refractory state in the NF-κB system. We asked how the variability between individual cells was generated (Fig. 5). We used a closely timed pair of TNFα pulses at 70 min interval as these discriminated cells into two pools that showed either a second response in IκBα degradation, or no response. We interpret this to mean that in one group cells have a refractory period of more than 70 min (non-responders), while in the other group cells have a refractory period of <70 min (responders). When the pair of pulses was repeated on the same cells several hours later, the presence or absence of a response was maintained. Daughter cell responses were also maintained in 85% of siblings. This implies that the refractory period was pseudo-stable, as characterized by a low switching probability over the timescale of the cell cycle. This mechanism enabled robust and reproducible digital responses in individual cells, with the timing of stimulation encoded in the fraction of responding cells. We hypothesize that this combination of digital- and analogue-encoding may induce a coordinated population-level response, enabling acute responses to temporal stimuli.

We predicted that the heterogeneous refractory period might be associated with cellular states encoded by the levels of protein in the TNFα transduction pathway, and mediated through a process downstream of TNFR and upstream of IKK. This mechanism has been represented in the mathematical model (in agreement with other NF-κB models^49^) by a simple nonlinear interaction between a generic IκB kinase kinase representing a complex TNFα transduction network (IKKK) and previously characterized NF-κB-dependent A20 negative feedback^50^ (Figs 3 and 4). In agreement with the model, siRNA knockdown of A20 protein increased the number of responding cells. A number of other proteins and interactions previously reported in the literature might also be involved, for example: TRAF adaptors^51^, RIP and TAK1 kinases^8^ as well as proteins involved in regulation of A20 enzymatic activity such as ABIN, RNF11, TAX1BP1 adaptors or the E3 ubiquitin-ligase Itch^25^,^26^,^52^. Response to TNFα (and IL-1β) might also involve other transcriptional networks, for example, mitogen activated protein (MAP) kinases or interferon regulatory factors (IRFs) (ref. 30), which also could constitute regulatory feedback and contribute to the observed behaviour. Experiments with alternate cytokine pulsing suggested that the TNFα stimulation had no effect on the IL-1β response; however, some dependency of the former on the IL-1β transduction pathway was found (Fig. 3 and Supplementary Fig. 9). To provide the simplest explanation for these data, we assumed that the signal cross-talk depended on respective levels of signal-specific IKKK and A20 inhibition; however, other mechanisms, for example, partial IKKK utilization by both pathways, cannot be excluded. It is possible that the cellular states within the IKK module might be controlled, for example, through a temporally stable epigenetic mechanism^6^ and this deserves further investigation. For example, the observed states are consistent with the reported long-term memory of protein levels^53^.

Refractory states in signalling have been previously associated with inhibition of cellular responses^54^,^55^. For example, endotoxin resistance was functionally linked with inhibition of over exuberant pro-inflammatory responses^56^. In contrast, we suggest that the refractory states in the NF-κB system (not only to TNFα and IL-1β, but also possibly to other input signals) might enable robust discrimination between different temporal cues. In accordance with this hypothesis, we showed that while in a refractory state to TNFα, cells responded to IL-1β stimulation (Fig. 6). Analysis of repeated pulses of TNFα, or alternating pulses of TNFα and IL-1β, suggested a strong correlation between single-cell NF-κB responses and the target gene expression (Supplementary Fig. 34). However, other downstream signalling systems including MAP kinases or IRFs^30^, stimulus-specific post-translational modification of the NF-κB (ref. 57) or timing of transcription and mRNA stability^47^ might also contribute to patterns observed for specific genes. At low doses, TNFα and IL-1β are the principal mediators of local inflammatory responses, however, at higher doses (>1 ng ml^−1^ in serum), TNFα (and IL-1β) becomes a danger signal that can lead to septic shock and ultimate death of the organism^58^. The level and timing of the signalling downstream of TNFα and IL-1β are therefore key parameters that dynamically control the activation and resolution of the immune response. We hypothesize that the refractory state might represent a mechanism in which responses to a single cytokine input are effectively dampened to avoid out-of-control activation, while multiple closely timed signals of more than one cytokine could synergistically activate an inflammatory response. This differential encoding of the temporal inputs is in part facilitated by the bow-tie topology of the NF-κB network, with the IKK effectively able to integrate multiple parallel upstream signals^22^,^23^. Other important mammalian signalling systems share a similar topology^59^, including epidermal growth factor^60^, G protein-coupled receptor^61^ and metabolic networks^62^. Hence, refractory states may be used in rapidly changing environments to fine tune patterns of differential gene expression. This may be a fundamental mechanism common to other cellular response systems.

Over the past decade, cellular heterogeneity has emerged as a common functional trait in many signalling systems with a number of studies suggesting a key involvement of stochastic gene regulation^4^. Intrinsic noise was thought to be particularly important for generation of heterogeneous NF-κB dynamics^2^,^14^,^45^. We previously argued that stochastic activation of IκB feedback regulated heterogeneity of NF-κB oscillation timing between individual cells, thus dampening potentially harmful fluctuations of NF-κB-dependent tissue-level cytokine secretion^14^,^63^. Intrinsic noise was also involved in the entrainment and amplification of NF-κB response to long-term periodic TNFα inputs^20^. In the present study, we identified an alternative source of cellular heterogeneity associated with pseudo-stable states embedded in the TNFα transduction network. We expect that many (if not all) network parameters may in fact exist in different states between individual cells (as suggested by measurements of protein^1^,^53^ and transcript levels^64^) and thus would contribute to the emergent behaviour. This behaviour is consistent with observed heritability of NF-κB responses^13^ and has been previously reported in MAP kinase and hypoxia-inducible factor signalling^7^. In agreement with our analyses, long-term repeat stimulation with TNFα pulses at 75 min intervals and lower resulted in a reduced system entrainment, possibly due to the refractory period^20^. This was exhibited by a fraction of cells responding with a frequency lower than that of the input (that is, effectively doubling the period of the NF-κB response). This also suggests that pulsing at intervals longer than the refractory period would result in better entrainment with most cells exhibiting the input frequency. Overall, we believe that the observed heterogeneous refractory states might be a part of the inherent design within the inflammatory response, to avoid out-of-control homogenous cell activation. This may be important for the balance between amplification and resolution of inflammation and its regulation could play a role in pathological inflammatory conditions.

## Methods

### Reagents and cell culture

SK-N-AS cells (ECACC 94092302 (ref. 10)) were cultured in minimum essential medium supplemented with 10% foetal calf serum (Gibco) and 1% NEAA (Sigma-Aldrich), and sub-cultured at densities between 80 and 90%. Stimulations were performed with 10 ng ml^−1^ (unless otherwise stated) of recombinant human TNFα or IL-1β (Calbiochem). Cells were stimulated with TNFα, washed three times with PBS and warmed culture medium was added. IL-1β neutralizing antibody (R&D Systems) was added (0.5 μg ml^−1^) to cells following IL-1β pulses and washed off with PBS before subsequent stimulation. Cells were routinely tested for mycoplasma contamination.

### Derivation of IκBα-eGFP SK-N-AS cell line

To preserve physiological control of the IκBα reporter construct, a BAC system was utilized. Using seamless recombineering techniques^65^ we replaced the stop codon of the IκBα gene within the BAC CTD-3214F11 with the eGFP coding sequence (see Supplementary Note 1 for detailed description). Therefore, the resulting IκBα-eGFP fusion was expressed under the control of large regions of flanking sequences (63 kb upstream and 92 kb downstream), and maintained the UTR/Exon/Intron gene structure (Fig. 1a). The BAC was retrofitted with a Neomycin selection marker. SK-N-AS cells were transfected with IκBα-eGFP BAC, single-cell sorted and a clonal line (C9) carrying the construct was selected. Subsequently, the cell line was transformed with a p65-mCherry lentiviral plasmid^37^. In pulsing experiments, a cell was classified as a ‘responder' if the gradient of the corresponding IκBα-eGFP trajectory at the time of stimulation was not positive; otherwise it was called a ‘non-responder'. This classification was independently verified by unsupervised clustering analysis (Supplementary Fig. 8). All experimental conditions and corresponding cell numbers are detailed in Supplementary Table 1.

### Development of the p65-mCherry expression vector

Human NF-κB p65 (accession number NM_021975) was amplified by PCR using primers flanked by gateway recombination sequences (forward primer: 5′-ATGGACGATCTGTTTCCCCTCATCT-3′, reverse primer: 5′-CGAGCTGATCTGACTCAGCAGGGGCT-3′). A third generation ‘pLNT' lentiviral transfer vector was used to enable Ubiquitin-ligase C promoter-mediated constitutive expression of N-terminally mCherry tagged amplified p65 gene^37^. Lentivirus production and transduction was performed as described fully in ref. 37.

### Western blotting

Cells were lysed in hot lysis buffer (1% (w/v) SDS, 10% (v/v) glycerol, 10% (v/v) β-ME, 40 mM Tris pH 6.8, 0.01% (w/v) bromophenol blue). Samples and ladder (NEB #P7712S, USA) were resolved on polyacrylamide gels. Proteins were transferred to nitrocellulose membranes (Protran BA-83, GE Healthcare), incubated at room temperature in blocking buffer (5% (w/v) non-fat milk powder in TBS-T), washed in TBS-T and incubated overnight with primary human antibody (p65 Cell Signaling Technology #8242, phospho-S536 p65 CST #3039, IκBα CST #9242, Gapdh CST #2118 and α-Tubulin CST #2144) at 1:1,000 dilution in blocking buffer. Membranes were washed (TBS-T × 3), and incubated with 1:1,000 or 1:2,000 HRP-conjugated immunoglobulin-G (CST #7074) for 2 h. Membranes were washed (TBS-T × 3) then incubated with Luminata Crescendo Western HRP Substrate (EMD Millipore Corp.) and signal was detected by exposure to Carestream Kodak BioMax MR film (Sigma-Aldrich). Membranes were stripped using Restore Western Blot Stripping Buffer (Thermo Scientific) before re-probing with another primary antibody where necessary. See Supplementary Fig. 35 for uncropped scans.

### siRNA knockdown

C9 cells were plated into 35 mm culture dishes 24 h before transfection. Transfection mix was prepared using the SK-N-AS transfection kit (Altogen Biosystems, Las Vegas, USA) according to the manufacturer's protocol, including complex condenser and transfection enhancer steps. Each dish was transfected with 100 nM of human A20 On-Target Plus siRNA or On-Target Plus non-targeting pool siRNA (both GE Dharmacon) 48 h before imaging. Cells were transferred to glass-bottomed dishes (Greiner Bio-One) 24 h before imaging.

### Nanostring analysis

Total RNA was extracted from wild-type SK-N-AS cells using the Roche High Pure RNA Isolation Kit. The nCounter Gene Expression assay (Nanostring Technologies, Seattle, USA) was performed according to the manufacturer's instructions. Transcript counts were normalized to the relevant housekeeping genes using the NanoStringNorm package within Bioconductor^66^. The protocol was followed where the geometric mean was used to summarize the positive (CodeCount) and housekeeping controls, with a stringent background correction applied (mean±2 s.d.). Differential expression of genes was assessed on log_2_-normalized data with hierarchical regression models, using the eBayes function within limma^67^. A 0.01 false discovery rate cutoff was used to determine statistical significance. All genes were clustered with respect to median log_2_ fold changes across replicates (treated/untreated, see Supplementary Data 1 for detailed analyses) and visualized as a heat map.

### Confocal microscopy

Cells were plated onto 35 mm-glass-bottomed dishes (Greiner Bio-One) and incubated on the microscope stage at 37 °C in humidified 5% CO_2_. Several Zeiss confocal microscopes were used (LSM Pascal, Exciter, 510meta, 710 or 780) with fluar × 40 numerical aperture (NA) 1.3 or plan-apochromat × 63 NA 1.4 objectives and appropriate excitation and emission wavelengths for the two fluorophores. Image capture was performed using the Zeiss software, either ‘Aim version 4.2 utilizing the Autofocus macro^68^' on the 5-series microscopes or ‘Zen 2010b SP1' on the 7-series microscopes. Quantification of IκBα-eGFP fluorescent signal of whole cells was performed using region of interest analysis in ‘Zen 2010b SP1'. The data were exported as mean fluorescence intensity. For quantification of p65-mCherry fluorescence, Cell Tracker (version 0.6)^69^,^70^ was used to estimate mean nuclear and whole-cell fluorescence level, which was expressed as a nuclear to total ratio.

### Evaluation of TNFα internalization

SK-N-AS cells were plated onto 4-compartment glass-bottomed imaging dishes (Greiner Bio-One) in culture medium and incubated at 37 °C in humidified 5% CO_2_ on the microscope stage. A Zeiss 780 confocal microscope with a plan-apochromat × 63 NA 1.4 oil objective was used with appropriate excitation and emission signal detection. Image capture was performed using Zeiss software ‘Zen 2010b SP1' to take Z stacks using a stack separation of 0.8–1.2 μM. Maximum intensity projections were used for image analysis. Human recombinant TNFα biotin conjugate (1 μg ml^−1^, Fluorokine, R&D Systems, Wiesbaden) was diluted to 25 ng ml^−1^ in either 20 μl of avidin-FITC (10 μg ml^−1^) or 2 μl avidin-Texas-Red (2 mg ml^−1^, Life Technologies) and made up to 50 μl with minimum essential medium. Cells were washed with PBS before stimulation. Cells were pretreated with 80 μM Dynasore hydrate (Sigma) for 1 h where applicable. For acid wash treatment, cells were cooled to 4 °C and incubated with acid wash buffer (150 mM NaCl, 100 mM glycine pH 2.5) for 3 × 2 min. Cells were fixed with 3.7% formaldehyde in PBS for 15 min at room temperature, then washed with PBS. Fixed samples were imaged on a Zeiss 780 confocal microscope as above, with a plan-apochromat × 40NA 1.3 oil objective.

### FACS analysis of TNFR1 level

SK-N-AS cells were scraped and fixed in 4% paraformaldehyde solution and then incubated on ice for 1 h with phycoerythrin conjugated TNFR1 antibody (Santa Cruz) according to the manufacturer's protocol. Specificity of the antibody was confirmed using interferon γ stimulated cells, which exhibited higher TNFR1 expression comparing to untreated cells. Samples were analysed with a FACSVerse Flow cytometer. To eliminate cell debris or aggregated cells, events with low or high side and forward scatter were excluded. Subsequent data analysis was performed with FlowJo Software.

### Mathematical modelling

In this work, we considered the structure^2^ and parameters^14^,^15^,^18^ of previously developed models of the NF-κB system to recapitulate responses to pulsatile TNFα and IL-1β stimulation (see Supplementary Note 5 for model development and validation). Additional single-cell imaging data^10^,^15^ including responses to low TNFα doses^2^,^18^ were also recapitulated. The model also fitted population-level experimental data (nuclear NF-κB, total IκBα and A20 protein and mRNA, and IKK activity levels) in response to continuous and pulsatile stimulation of TNFα (of varying duration and concentration) in WT and A20 knockout cells^21^,^28^,^32^,^40^. Population-level heterogeneity was modelled by (1) normal distributions of network parameters, 
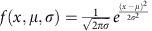
, with mean *μ* equal to nominal values of biochemical parameters simulated, and s.d. *σ*=0.3 *μ*, or (2) stochastic transcription of IκBα and A20 feedback genes in single cells^14^. Each model simulation was directly comparable to single-cell time-lapse microscopy data. A cell was classified as a ‘responder' if the net peak amplitude of the nuclear NF-κB (calculated with respect to the nuclear NF-κB level at the time of stimulation) was >15% of the total level per cell; otherwise it was called a ‘non-responder'.

Simulations were performed in Matlab R2013a environment (see Supplementary Table 7 for all simulated conditions and Supplementary Software for computer codes). Power spectrum analysis was performed in Matlab using DFFT function.

### Distribution of the refractory period

The refractory period is defined as the time period for which cells are unresponsive to TNFα (following initial stimulation). The distribution of refractory periods (Fig. 2g) was calculated based on fractions of responding cells measured at consecutive time points (based on data in Fig. 2f). For example, the fraction of responding cells decreased from 70 to 30% at 70 and 60 min pulse intervals, suggesting that ∼40% of cells had a refractory period between 60 and 70 min.

### Global sensitivity analysis

Latin hypercube sampling was performed to calculate global parameter sensitivity with respect to model outputs^43^. For each model parameter, the range of ±50% around the nominal value was uniformly divided into equal *k=*1,000 slices. Values from each parameter range were then sampled randomly without replacement to ensure that the entire parameter range was explored. The model output was then simulated for each of the randomized *k* combinations of parameter values. A Spearman correlation coefficient was then calculated between each parameter and model output of interest (for example, fraction of responding cells, amplitude of NF-κB activation, period of oscillations and so on).

### Statistical analyses

Statistical analyses were performed in GraphPad Prism V 6.0. For normally distributed samples (as assessed with D'Agostino and Pearson test) two-sided Student *t*-test was performed, otherwise nonparametric tests were applied (as indicated in the text). Goodness-of-fit comparison was performed using Fisher's exact test. Sample sizes were selected based on the number of replicates (up to three) performed for each experiment. Statistical analyses are detailed in Supplementary Data 1.

### Data availability

The data that support the findings of this study are available from the corresponding authors on request.

## Additional information

**How to cite this article:** Adamson, A. *et al.* Signal transduction controls heterogeneous NF-κB dynamics and target gene expression through cytokine-specific refractory states. *Nat. Commun.* 7:12057 doi: 10.1038/ncomms12057 (2016).

## Supplementary Material

Supplementary Information Supplementary Figures 1-35, Supplementary Tables 1-6, Supplementary Notes 1-5, and Supplementary References

Supplementary Data 1 Gene expression and imaging analysis of pulsatile TNFα and IL-1β stimulation

Supplementary Movie 1 Responses to equilibrated TNFα pulses. Confocal microscopy movie of representative C9 cells expressing IkBα-eGFP fusion stimulated with TNFa at two 70 min intervals separated by 4h equilibration phase (as displayed). Shown is a "responsive" and "non-responsive" cell at top and bottom of the imaging field, respectively. Time from the start of the experiment depicted in minutes.

Supplementary Software Matlab scripts for computer simulations of NF-kB mathematical models.
