# Supplementary material for: Signal transduction controls heterogeneous NF-κB dynamics and target gene expression through cytokine-specific refractory states
Source: Nat Commun. 2016 Jul 6;7:12057. doi: 10.1038/ncomms12057 (PMC4935804; doi:10.1038/ncomms12057)
Supplement: Supplementary Information — Supplementary Figures 1-35, Supplementary Tables 1-6, Supplementary Notes 1-5, and Supplementary References [file ncomms12057-s1.pdf]

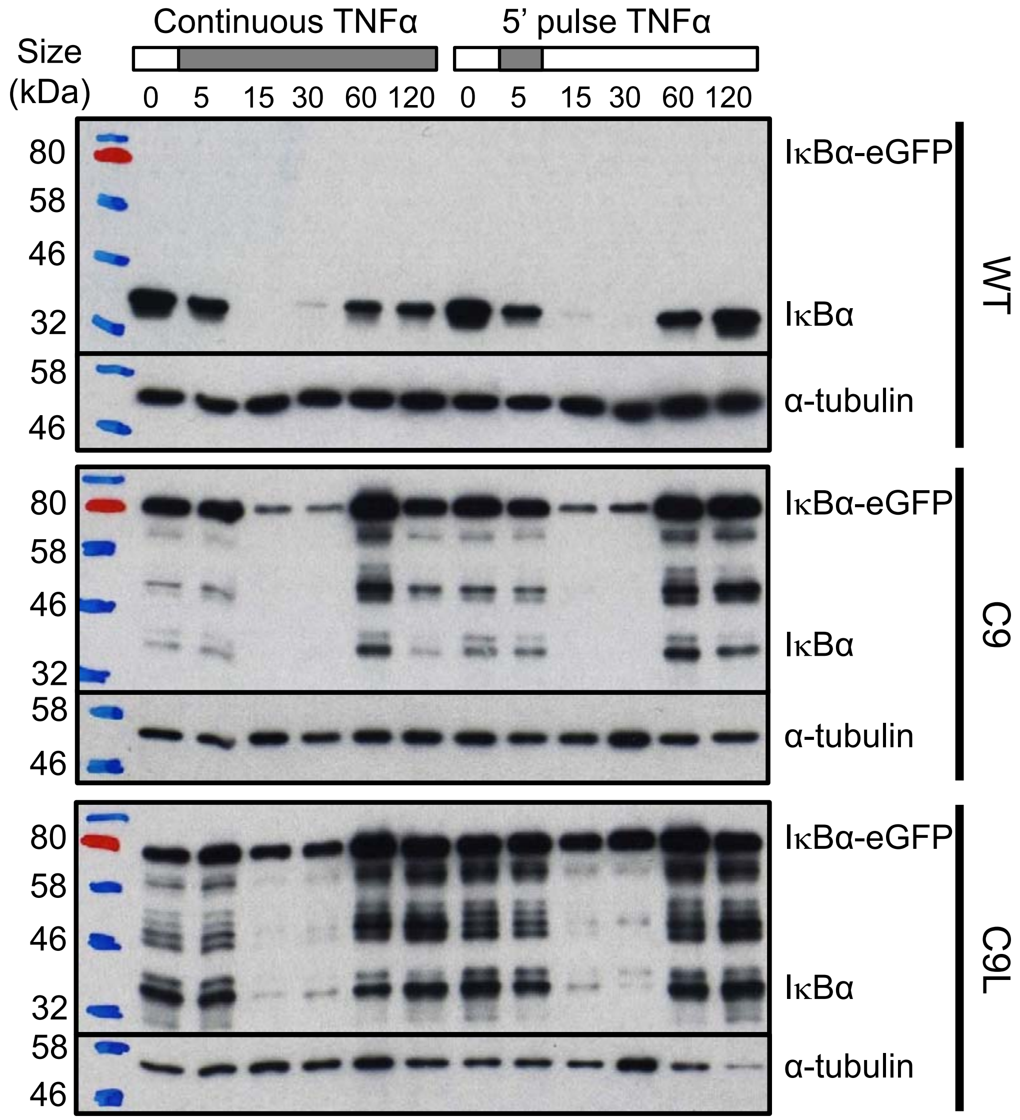

Supplementary Figure 1. Immunoblotting analysis of IκBα protein level in WT, C9 and C9L SK-N-AS cells. Cells stimulated with a continuous or a 5 min pulse of 10 ng ml^{-1} of TNFα. Whole cell lysates harvested at indicated times after simulation (in min). Shown are the levels of the endogenous and exogenous (IκBα-eGFP), as well as α-tubulin loading control.

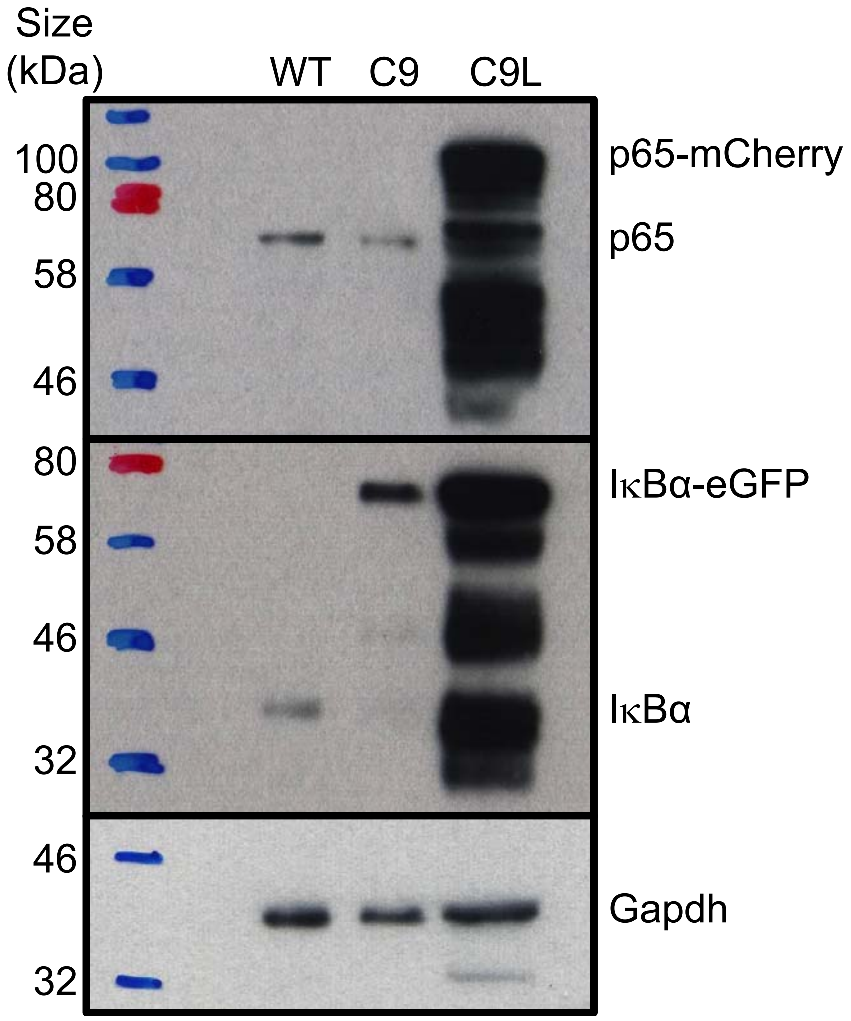

Supplementary Figure 2. Immunoblotting analysis of IκBα and NF-κB p65 levels in WT, C9 and C9L SK-N-AS cells. Shown are unstimulated cells (with Gapdh loading control).

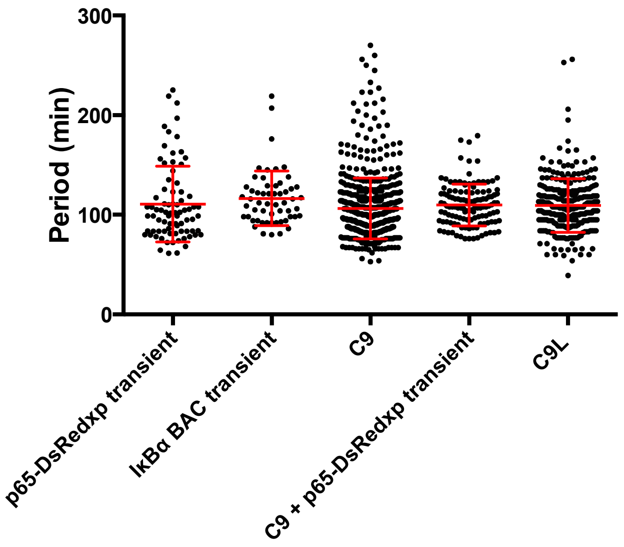

Supplementary Figure 3. Timing of NF-κB oscillations in different model systems. Shown are individual peak-to-peak timings for C9 (163 cells) and C9L cells (30 cells), respectively, with corresponding mean (± s.d.) per condition (data from Fig. 1F). Also shown are SK-N-AS cells transiently transfected with p65-DsRedxp plasmid (34 cells, taken from [1]) or IκBα-eGFP BAC (15 cells), or C9 cells transfected with p65-DsRedxp plasmid (34 cells). Peak-to-peak defined as the time between consecutive troughs of the total IκBα-eGFP signal. Cells stimulated with 10 ng ml^{-1} of TNFα.

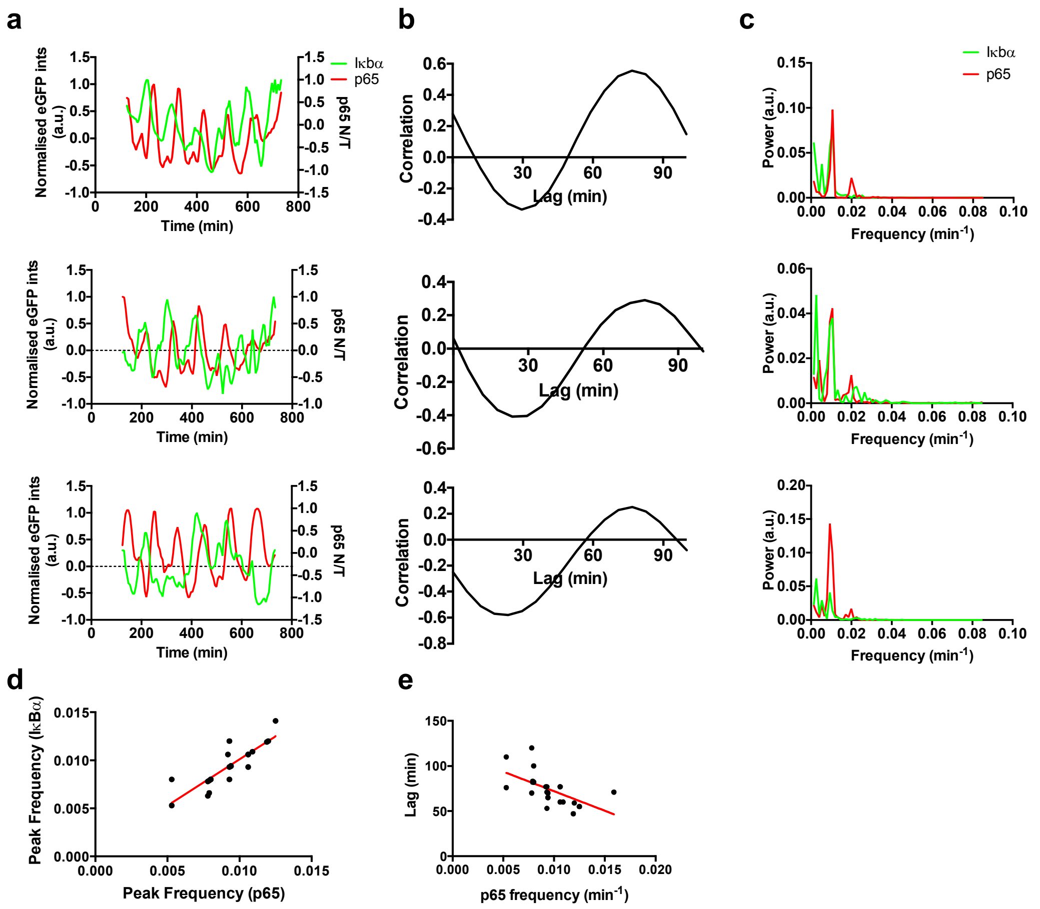

Supplementary Figure 4. Analysis of IκBα and p65 oscillation in C9L cells stimulated with continuous TNFα. Shown are (a) representative IκBα-eGFP and p65-mCherry single cell traces, (b) the cross-correlation function between the corresponding IκBα-eGFP and p65-mCherry single cell traces, (c) the power spectra calculated for IκBα-eGFP (in green) and p65-mCherry (in red) traces, (d) the correlation between peak IκBα-eGFP and p65-mCherry oscillation frequency (in red a fitted linear regression line) (e) the correlation between lag times and p65-mCherry oscillation frequency. 17 C9L cells were analysed (in d and e) and single cell traces were normalized to levels at 0 min, trajectories beyond 120 min were analysed.

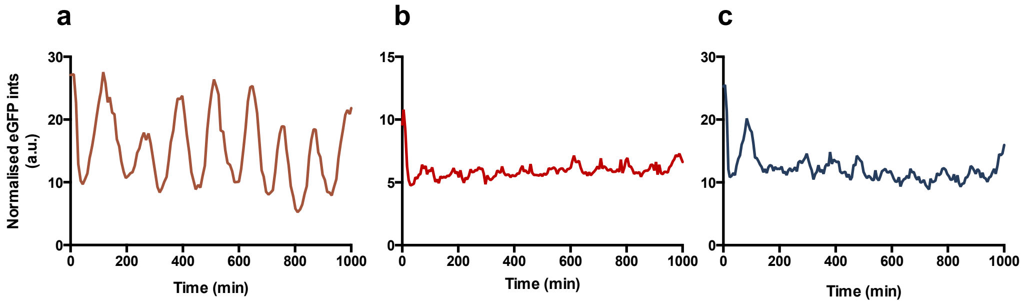

Supplementary Figure 5. Individual cells have unique oscillation frequency. Representative IκBα-eGFP single C9 cell traces stimulated with continuous TNFα used to calculate power spectra in Fig. 1g (using the same colour scheme).

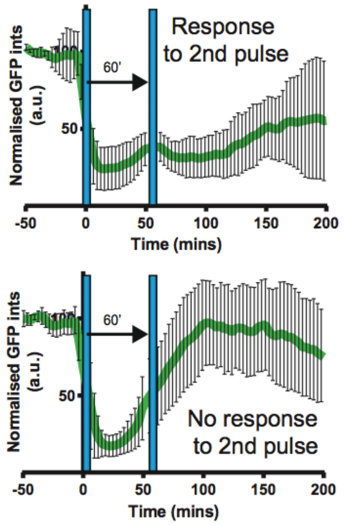

Supplementary Figure 6. Response to two 5 min TNFα pulses applied at 0 and 60 min. Shown are the mean (± s.d.) of the normalized single-cell total IκBα-GFP fluorescent intensity in C9 cells from Fig. 2b. Cells were clustered in responding and non-responding classes based on the IκBα-gradient at the time of the 2nd pulse. Timing of TNFα stimulation represented with a blue bar.

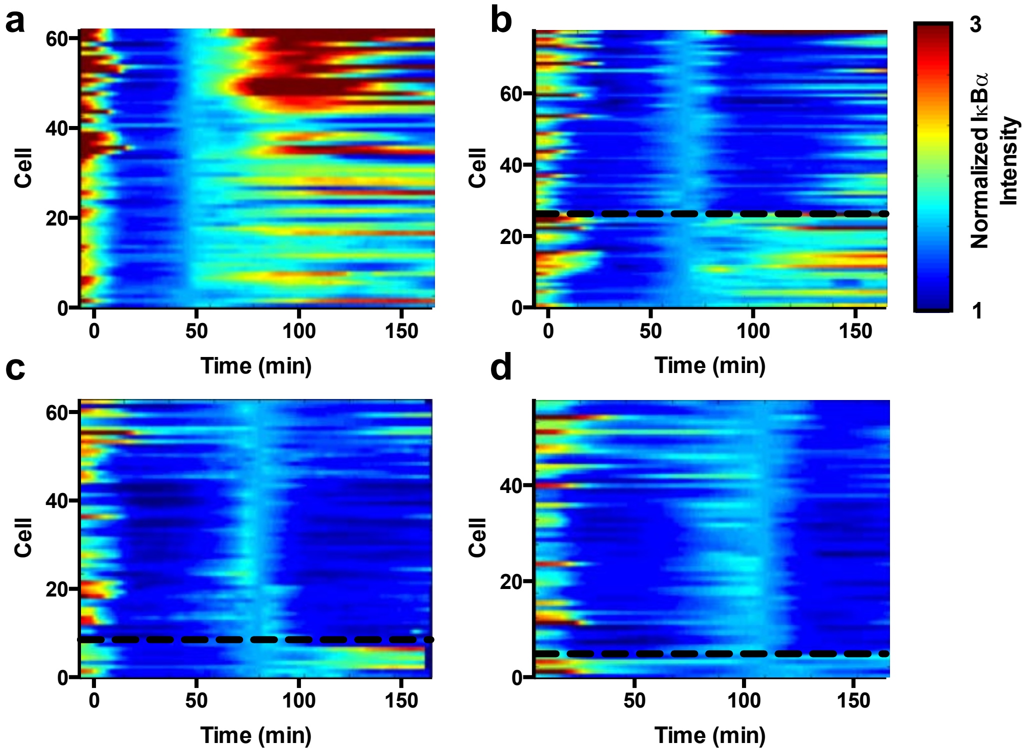

Supplementary Figure 7. Response to TNFα pulses. Single-cell responses to 5 min TNFα pulses at (a) 50, (b) 70, (c) 80 and (d) 100 min interval. Shown is the heat map of individual cell traces (Y axis), clustered by the normalized total IκBα-eGFP single-cell trajectories, per condition. Data from Fig. 2f normalized to the IκBα-eGFP level at the time of 2nd pulse, time depicted in minutes. Broken-lines indicate "*responding*" (above) and "*non-responding*" (below) cell groups.

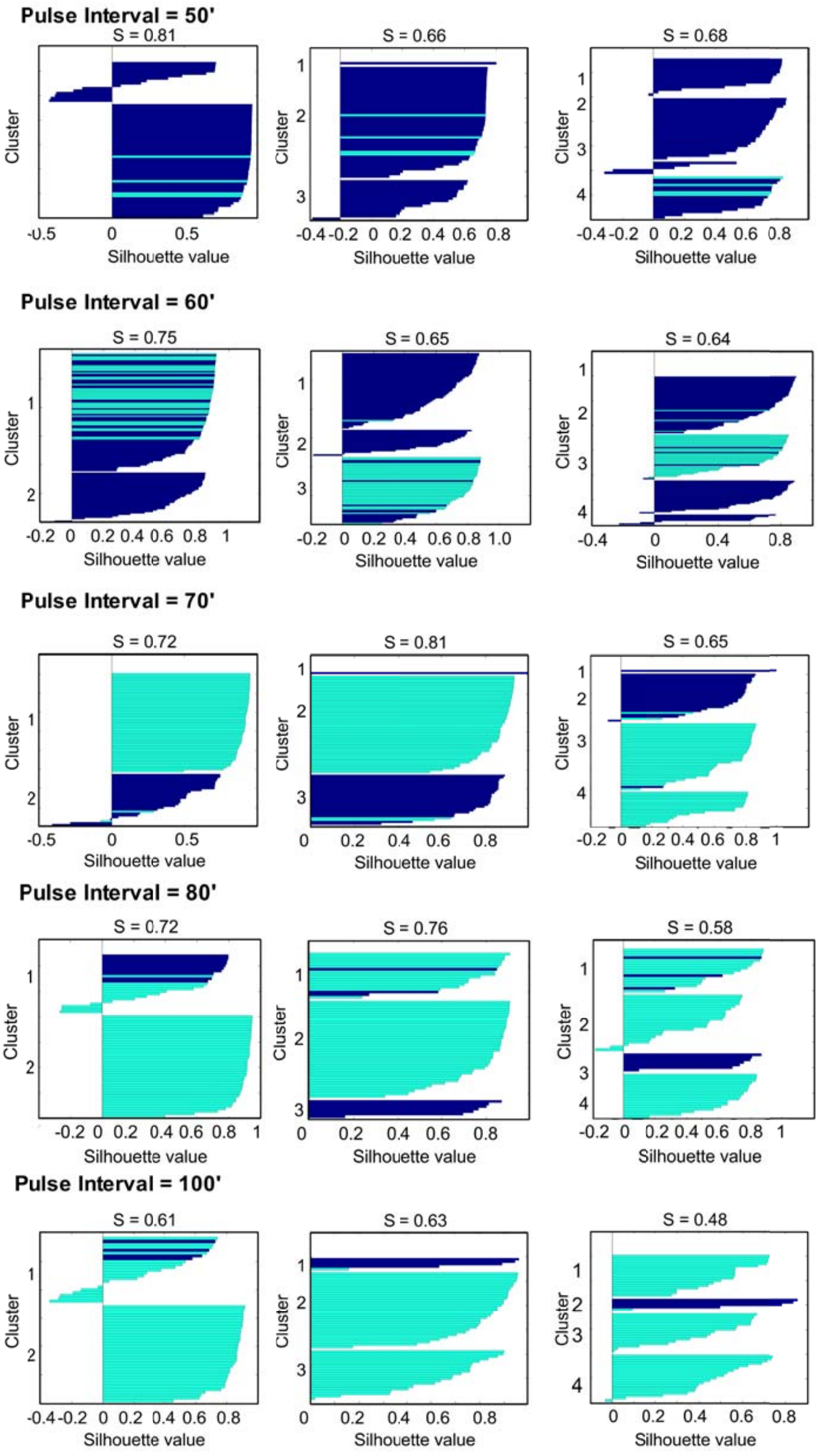

Supplementary Figure 8. Clustering analysis of single cell responses to TNFα pulses. Shown is Silhouette plot and coefficients (S) for each data set (from Fig. 2f) as a function of number of clusters (2, 3 and 4, respectively, from

left to right). Cells classified as "*responders*" base on the IκBα-eGFP gradient at the time of the 2nd peak depicted with green colour.

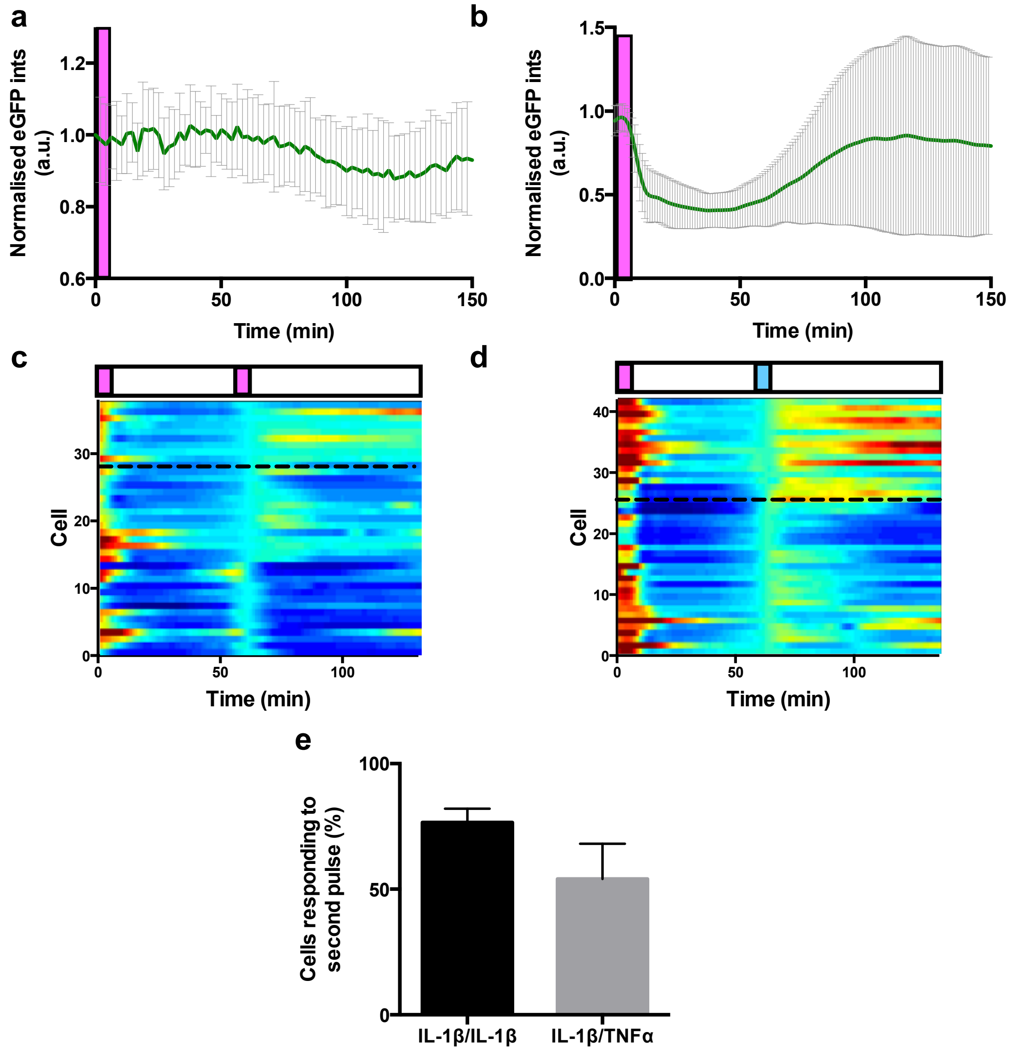

Supplementary Figure 9. IL-1β stimulation induced a refractory state. (a) C9 cells stimulated with IL-1β (depicted with a pink bar) and IL-1β neutralising antibody. Shown is mean and s.d. of 35 single cell traces. (b) C9 cells stimulated with a single 5 min IL-1β pulse (wash performed with media containing IL-1β neutralising antibody at 5 min after stimulation). Shown is mean and s.d. of 37 single cell traces. (c) Responses to two 5 min IL-1β pulses at 60 min interval (washes performed with media containing IL-1β neutralising antibody). Shown is the heat map of individual cell traces (Y axis), clustered by the normalized total IκBα-eGFP single-cell trajectories (from 39 single cells). Data normalized to the IκBα-eGFP level at the time of 2nd pulse, time depicted in minutes. Broken-lines indicate "*responding*" (above) and "*non-responding*" (below) cell groups. (d) Responses to a 5 min IL-1β pulse followed by a 5 min TNFα pulse at 60 min (first wash performed with media containing IL-1β neutralising antibody). Shown is data from 42 single C9 cells, IL-1β and TNFα pulses depicted with pink and blue bars respectively. (e) Fraction of responding cells from data in c and d (shown is mean and data range of two replicates).

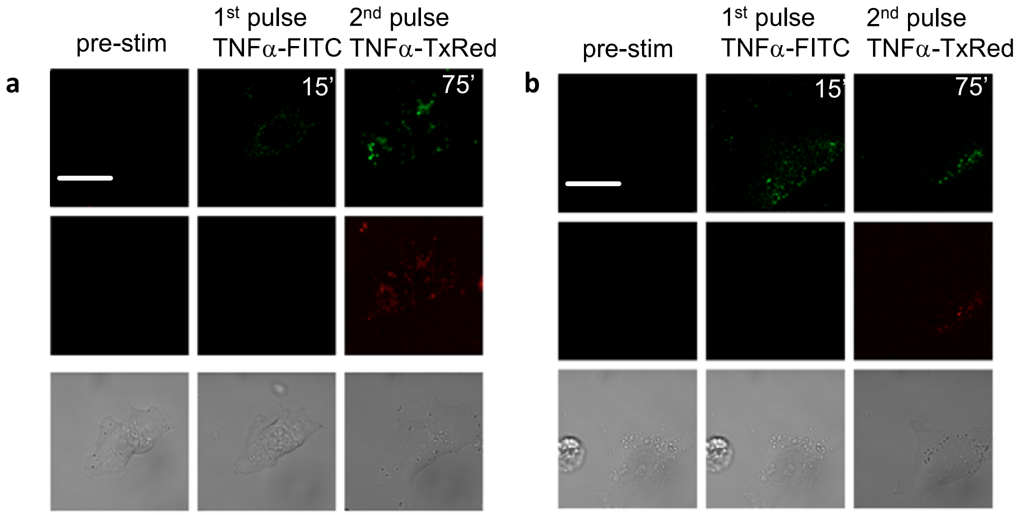

Supplementary Figure 10. Analysis of TNFα internalisation. Confocal microscopy images of WT cells stimulated with two pulses of fluorescently labelled TNFα at 60 min interval. (Two different fields on the imaging dish shown in a and b). FITC-conjugated TNFα (top panel, in green) was applied at 0 min for 5 min, washed with media, and fluorescence intensity measured at 15 min after the start of the experiment. Tx-Red-conjugated TNFα (middle panel, in red) was applied on the same cells at 60 min, washed with media, and then fluorescence intensity measured at 75 min after the start of the experiment. Corresponding bright field images are shown at the bottom. Scale bar 20 μm.

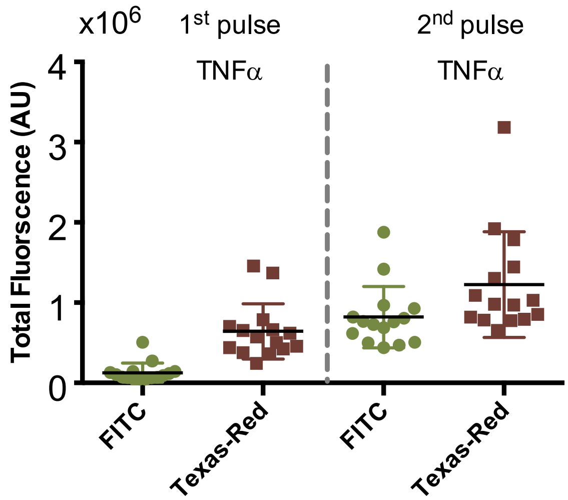

Supplementary Figure 11. Quantification of TNFα internalization levels. WT cells were stimulated with two pulses of fluorescently labelled TNFα at 60 min interval. Tx-Red-conjugated TNFα was applied at 0 min. FITC-conjugated TNFα was applied on the same cells at 60 min after the start of the experiment. Shown are the total fluorescence levels in 15 cells measured at 15' and 75' min after the start of the experiment.

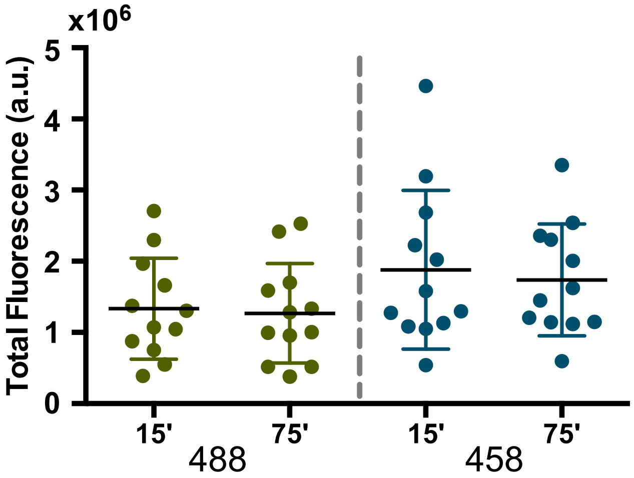

Supplementary Figure 12. Analysis of TNFα-FITC internalization. WT cells were stimulated with a 5-min pulse of FITC labelled TNFα. Shown are the total fluorescence levels in 12 cells measured at 15 and 75 min after the start of the experiment excited either with a 488 and 458 nm laser line.

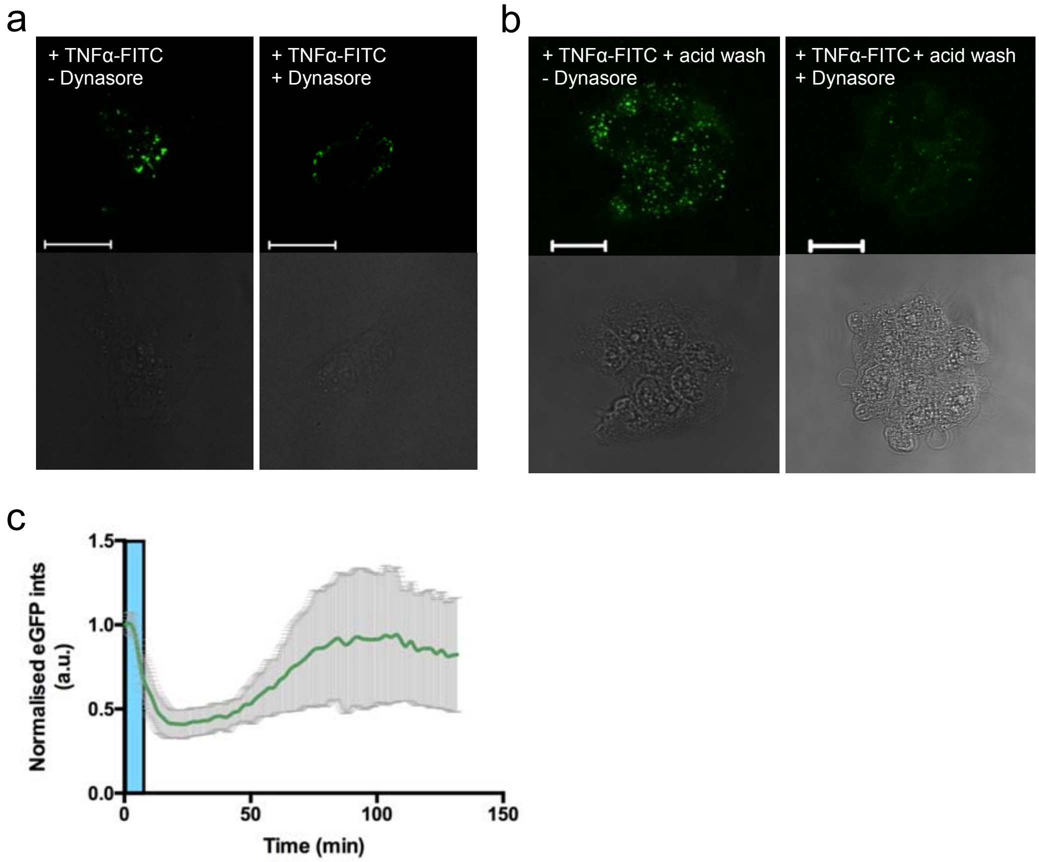

Supplementary Figure 13. NF-κB signalling is not affected by endocytosis. (a) Confocal microscopy images of WT SK-N-AS cells stimulated with a 5-min FITC-labelled TNFα pulse (measured at 40 min) with (left panel) or without (right panel) treatment with endocytosis inhibitor dynasore. Scale bar represents 20 μm. (b) Maximal intensity projections of confocal microscopy images for WT SK-N-AS cells stimulated as in a, then treated with acid wash buffer and fixed prior to imaging. Scale bar represents 20 μm. (c) Response to a 5 min TNFα pulse following a 1 h treatment with dynasore (as in a). Shown are mean and standard deviation of 25 single C9 cell traces.

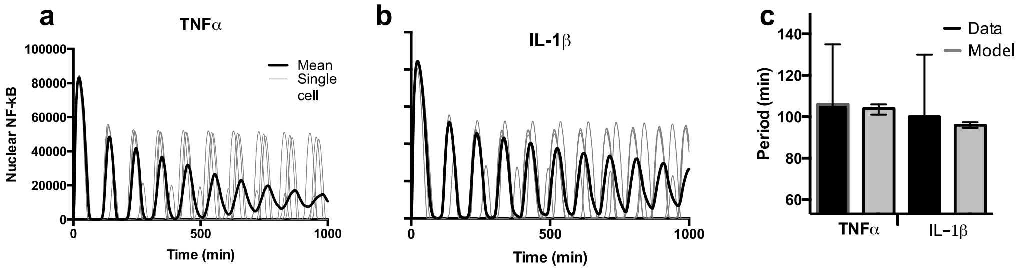

Supplementary Figure 14. Simulated single cell NF-κB dynamics in response to continuous treatments. Shown are responses to 10 ng ml^{-1} (a) TNFα and (b) IL-1β stimulation. Shown are representative single cell responses (in grey) and the corresponding population average, 300 cells (in black) of nuclear NF-κB in a model with distributed IKKK level. (c) NF-κB oscillation period (mean ± s.d.) in the model and the data (from Fig. 1f).

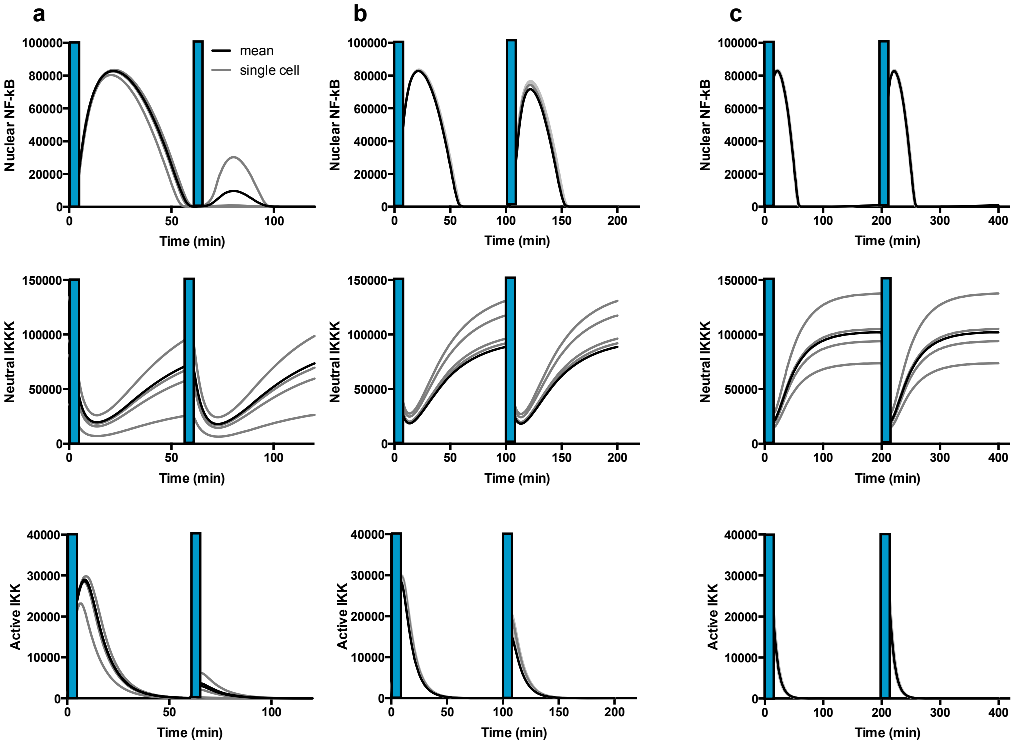

Supplementary Figure 15. Model simulation of the IKK signalling module in response to two TNFα pulses at different time intervals. Shown are simulations of response to TNFα pulses at (a) 60, (b) 100 and (c) 200 min intervals. Shown are representative single cell responses (in grey) and population average (from 300 cells, in black) of nuclear NF-κB (*NFkBn*), neutral IKKK (*IKKKn*) and active IKK (*IKKa*) in number of molecules.

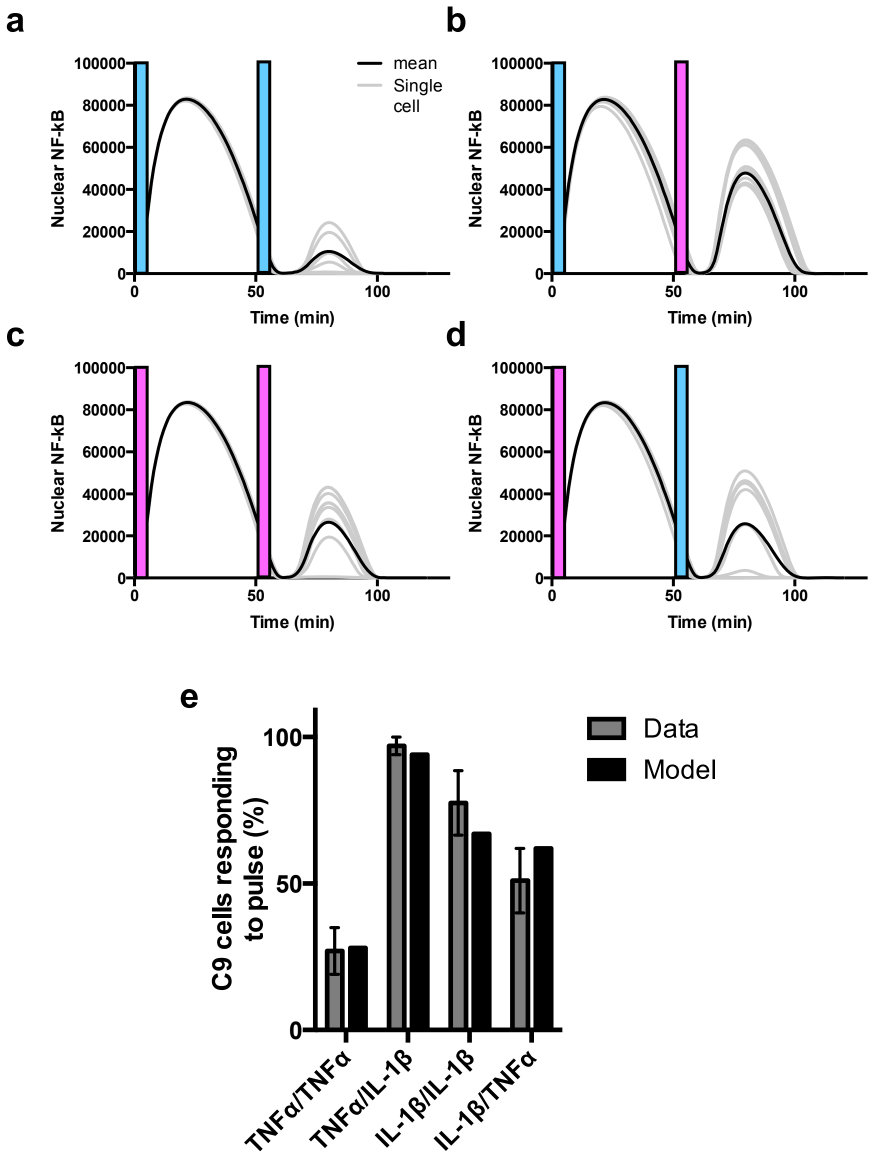

Supplementary Figure 16. Model simulation of the response to alternate TNFα and IL-1β pulses at a 60 min interval. Shown are simulated representative single cell trajectories of the nuclear NF-κB (in number of molecules) and the population average (300 cells, in black). TNFα and Il1β pulses depicted with blue and pink bars, respectively. Shown in (a) TNFα/TNFα, (b) TNFα/IL-1β, (c) IL-1β/IL-1β, (d) IL-1β/TNFα. (e) Fraction of cells responding to 2nd pulse from A-D and corresponding data (Fig. 2b and Supplementary Figure 9). Shown is the mean and s.d. (TT, TI) of three replicates or data range (IT, II) of two replicates.

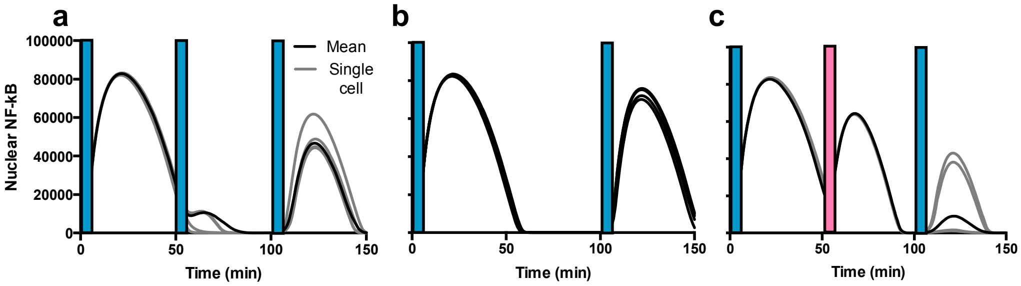

Supplementary Figure 17. Simulated nuclear NF-κB following TNFα (blue) and IL-1β (pink) pulses at 50 min interval. Shown are representative cells (grey) and mean (300 cells, in black) simulated with distributed IKKK parameter values under (a) TTT, (b) T_T and (c) TIT conditions as in Fig. 6.

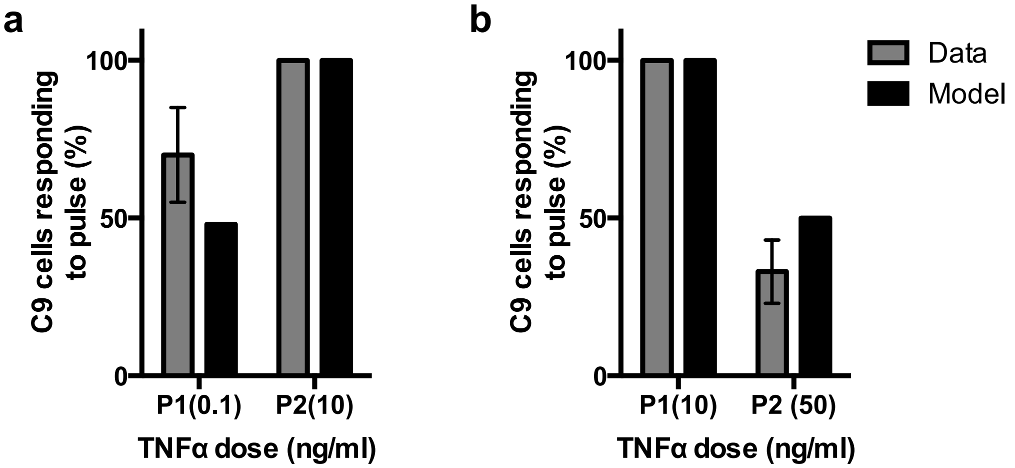

Supplementary Figure 18. Dose-dependent activation of the refractory state. Shown is a fraction of C9 cells responding to two TNFα pulses at a 60 min interval. (a) First pulse (P1, applied at 0 min): 0.1 ng ml^{-1}, second pulse (P2, applied at 60 min): 10 ng ml^{-1} (b) P1: 10 ng ml^{-1}, P2: 50 ng ml^{-1} pulse. Shown is the analysis of (a) 78 C9 cells (in grey, based on two replicates) and 300 simulated cells (in black, using the stochastic receptor module in the model), and (b) 30 C9 cells (grey bars, based on two replicates) and 300 simulated cells (in black, using deterministic receptor module).

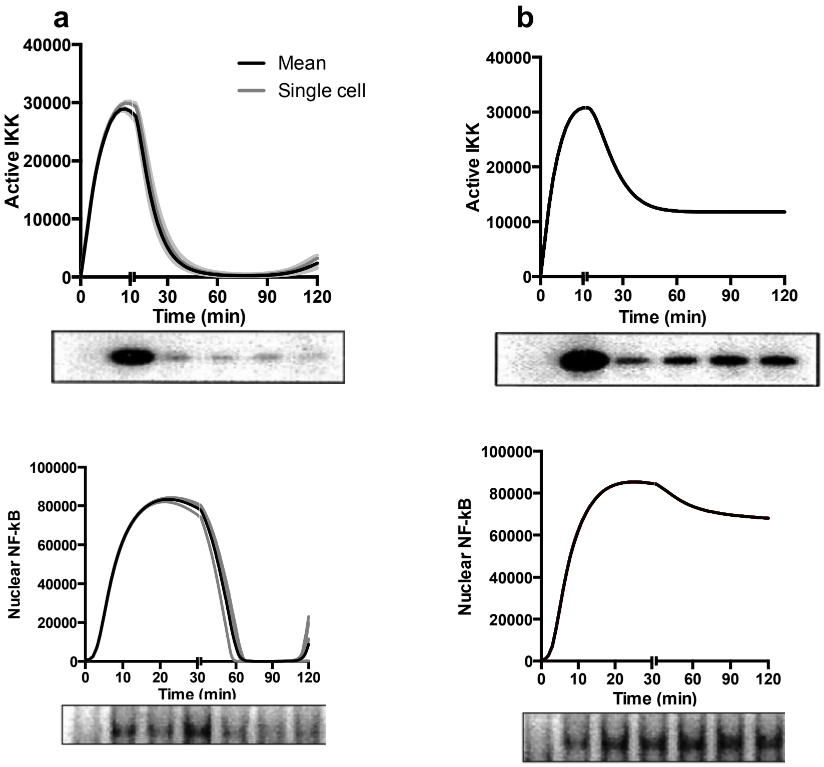

Supplementary Figure 19. A20 feedback regulation following continuous 10 ng ml^{-1} TNFα stimulation. Shown are representative cells (in grey) and population average (black) simulated with nominal parameter values. Number of nuclear NF-κB and active IKK molecules shown in (a) WT and (b) A20 KO cells. The model recapitulated MEFs data from [2] as depicted with the blots for the IKK activity assay (top) and electrophoretic mobility shift assay for the NF-κB activity (bottom).

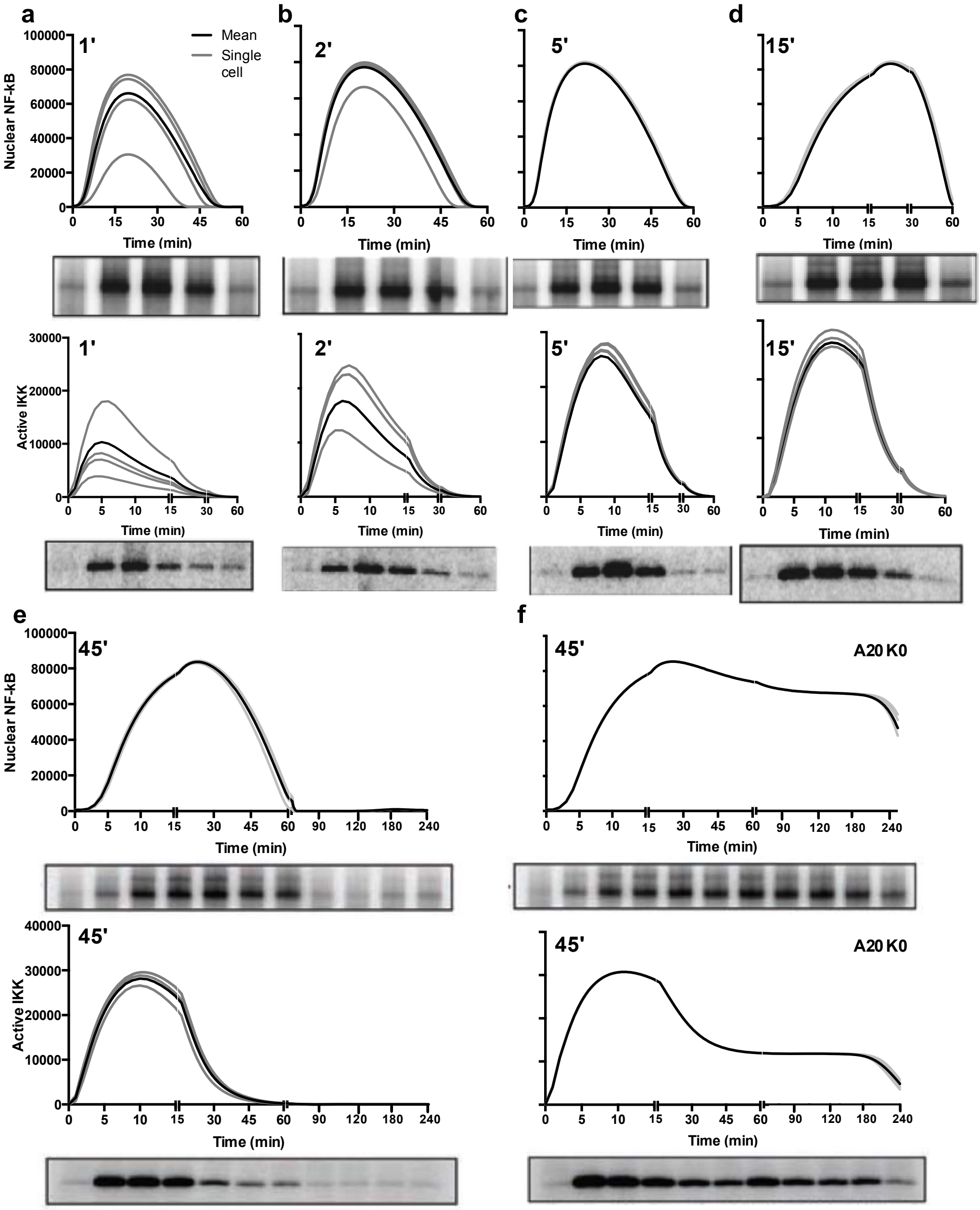

Supplementary Figure 20. A20-regulated IKK and NF-κB activity following a single 1 ng ml^{-1} TNFα pulse of different duration. (a-d) The comparison between model simulations and the level of nuclear NF-κB activity (based on electrophoretic mobility shift assay, top) and IKK activity (bottom) in response to a single 1, 2, 5 and 15 min TNFα pulse. Shown is the simulated number of active IKK and nuclear NF-κB molecules between (representative cells and population average (300 cells, black), and the blotting data from [3]). (e and f) Responses to 45 min pulse in WT and A20 KO MEFs. Blotting data reproduced from [4].

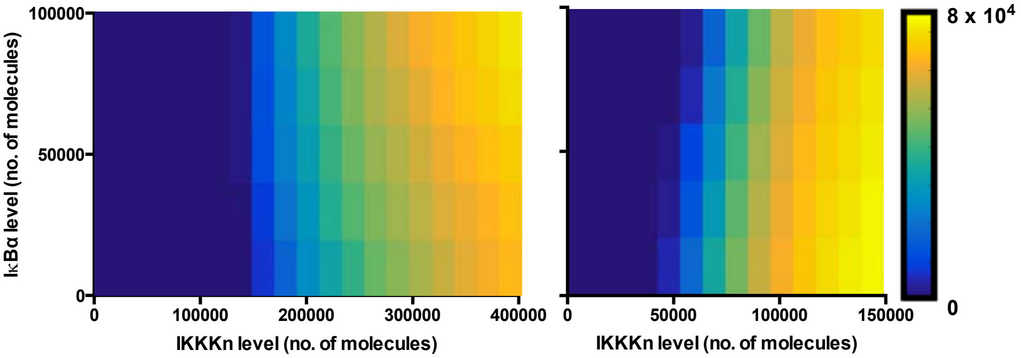

Supplementary Figure 21. Refractory state is independent of IκBα negative feedback. The amplitude of NF-κB nuclear translocation in response to TNFα pulses at 60 (left) and 80 min (right) depended on the simulated IKKKn, but not IκBα protein level. Region in yellow corresponds to parameter values that induce NF-κB activation at the 2nd pulse, while in the blue region no activation was elicited (scale as in Fig. 4b corresponding to the net nuclear NF-κB translocation at the time of 2nd TNFα pulse).

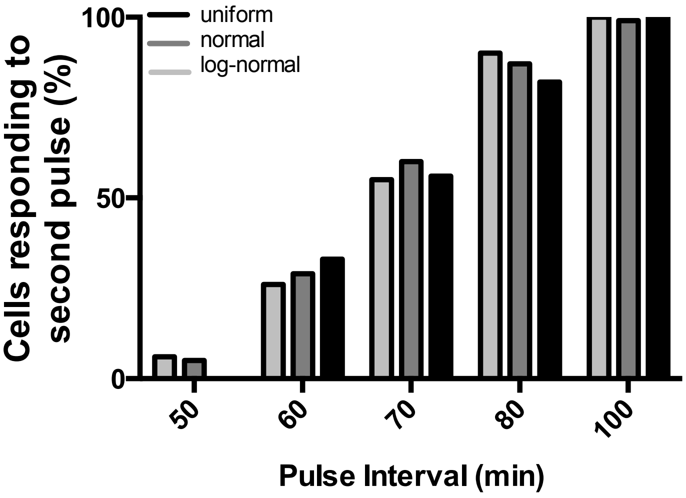

Supplementary Figure 22. Refractory period can be explained by different IKKK distributions. Shown are 300 single cells simulated with the total IKKK levels assuming normal (as in Fig. 4c-e, $\mu = 10^5$, $\sigma = 0.3^* \mu$), log-normal ($\mu = 11.5$, $\sigma = 0.3$) or uniform ($U(5x10^5,15x10^5)$) distribution.

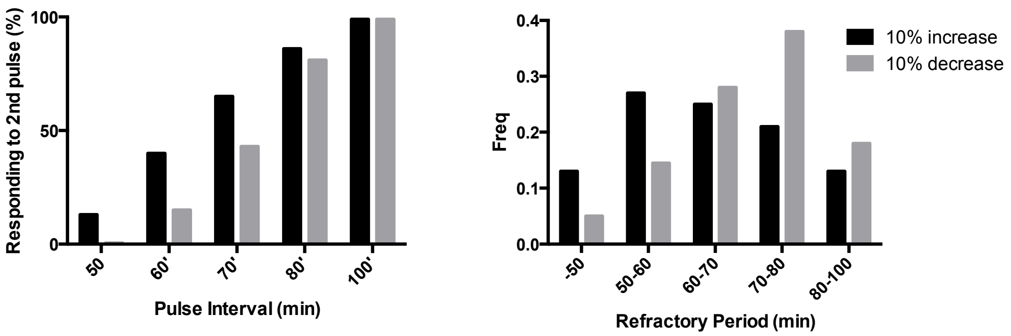

Supplementary Figure 23. Refractory period distribution depends on the mean IKKK level. Shown is the fraction of responding cells (left) and the refractory period distribution (right) in response to 10% change of mean μ (nominal value of 10^6) of the IKKK distribution. 300 single cell simulated per condition, standard deviation set to σ=0.3μ', were μ' is the respective mean.

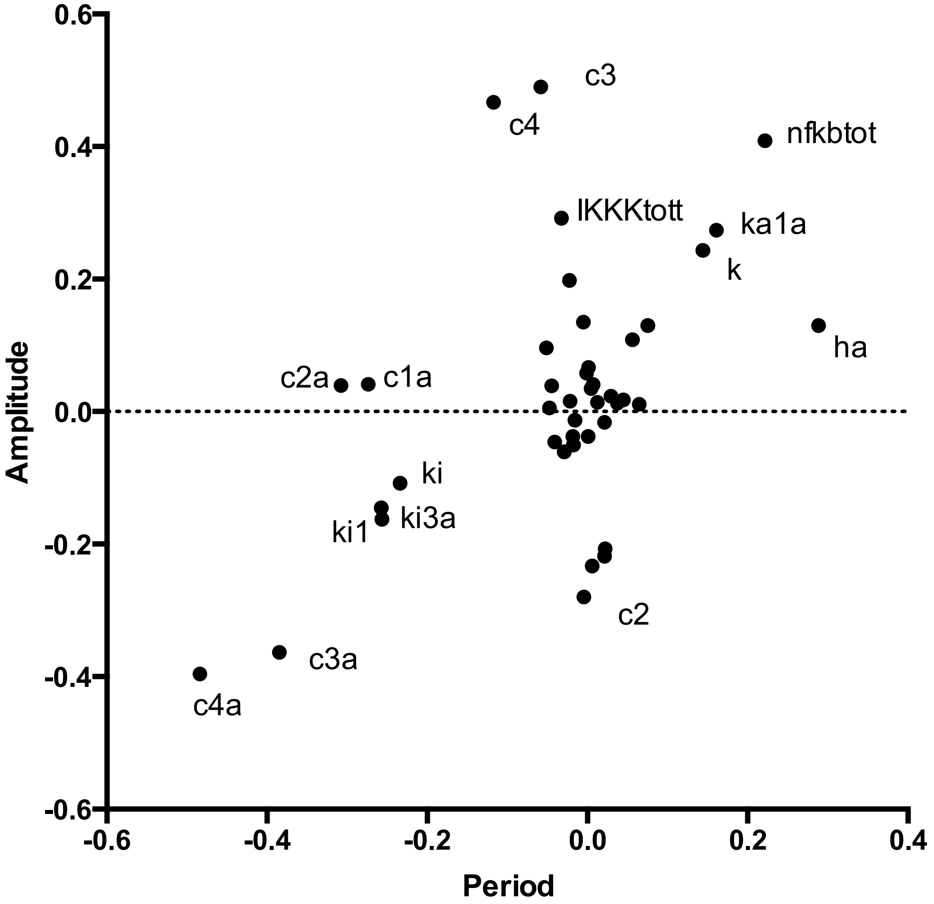

Supplementary Figure 24. Global sensitivity analysis of NF-κB oscillations in response to continuous TNFα stimulation. Shown is the correlation between sensitivity scores for the amplitude (on Y-axis) and the period (on X-axis) of NF-κB nuclear oscillations. Most sensitive parameters (labelled on the graph) include those related to IκBα and A20 transcription, translation and half-life, NF-κB transport, total level of NF-κB and IKKK.

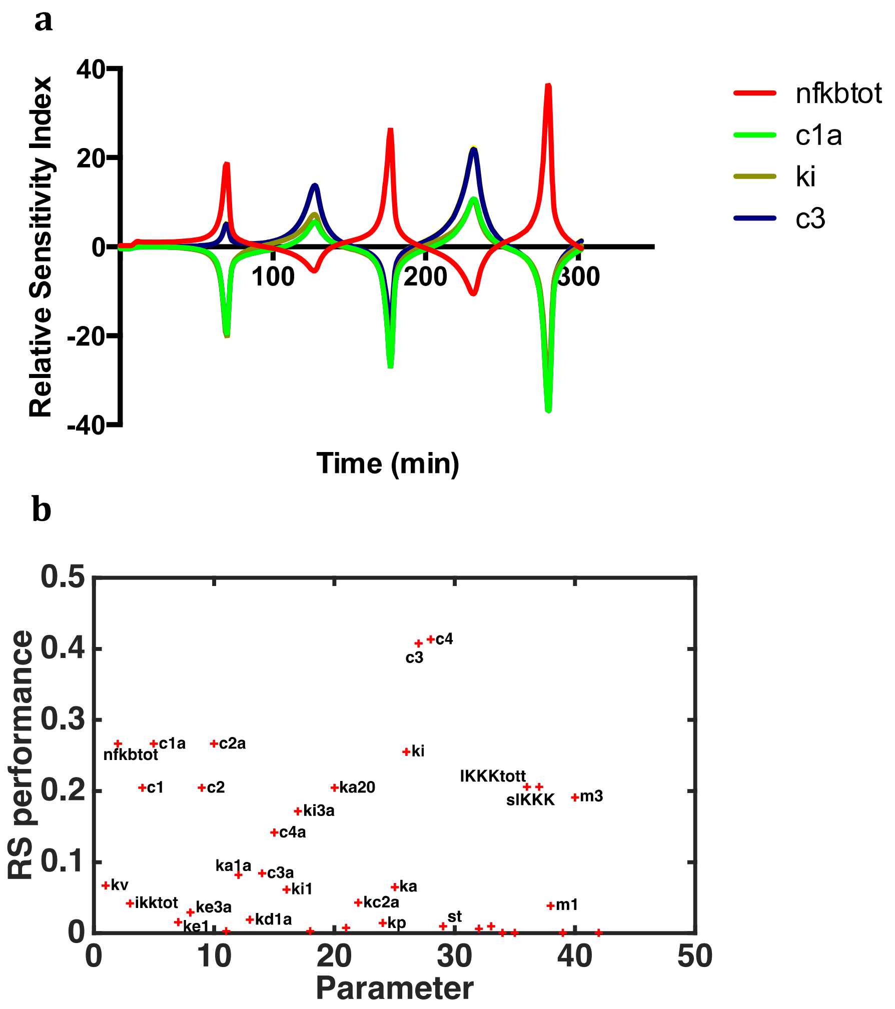

Supplementary Figure 25. Dynamic sensitivity analysis of NF-κB activation in response to continuous 10 ng ml^{-1} TNFα stimulation. (a) Temporal sensitivity coefficients of NF-κB p65 nuclear translocation trajectory. Colour lines correspond to most sensitive model parameters, IκBα transcription rate (c1a), IKK inactivation rate (*ki*), A20 protein half-life (c3), and NF-κB level *(nfkbtot).* (b) Relative sensitivity (RS) performance is shown with respect to nuclear NF-κB level for all model parameters (calculated as an average temporal sensitivity coefficient over the 300 min trajectory). Names of most sensitive parameters indicated on the graph.

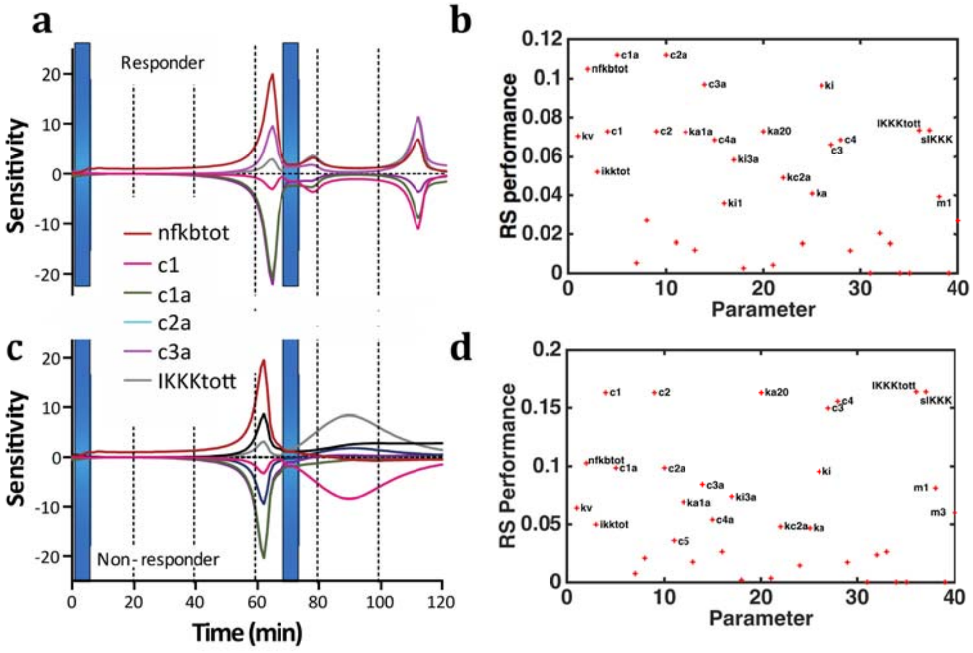

Supplementary Figure 26. Temporal sensitivity coefficients of NF-κB p65 nuclear translocation trajectory in response to TNFα pulses at 70 min interval. Coloured lines correspond to sensitivity scores calculated for different model parameters: IκBα and A20 (*c1*, *c1a*, *c3a*), IKKK (*IKKKtott)* and NF-κB level *(nfkbtot) for a "responding*" (a, b) with relative sensitivity index (RS, calculated as an average sensitivity coefficient over the 120 min trajectory), and *"non-responding*" (c, d) cell. Responding and non-responding cells simulated with total IKKK equal to 1.5 x 10^5 and $7 x 10^4$ molecules, respectively.

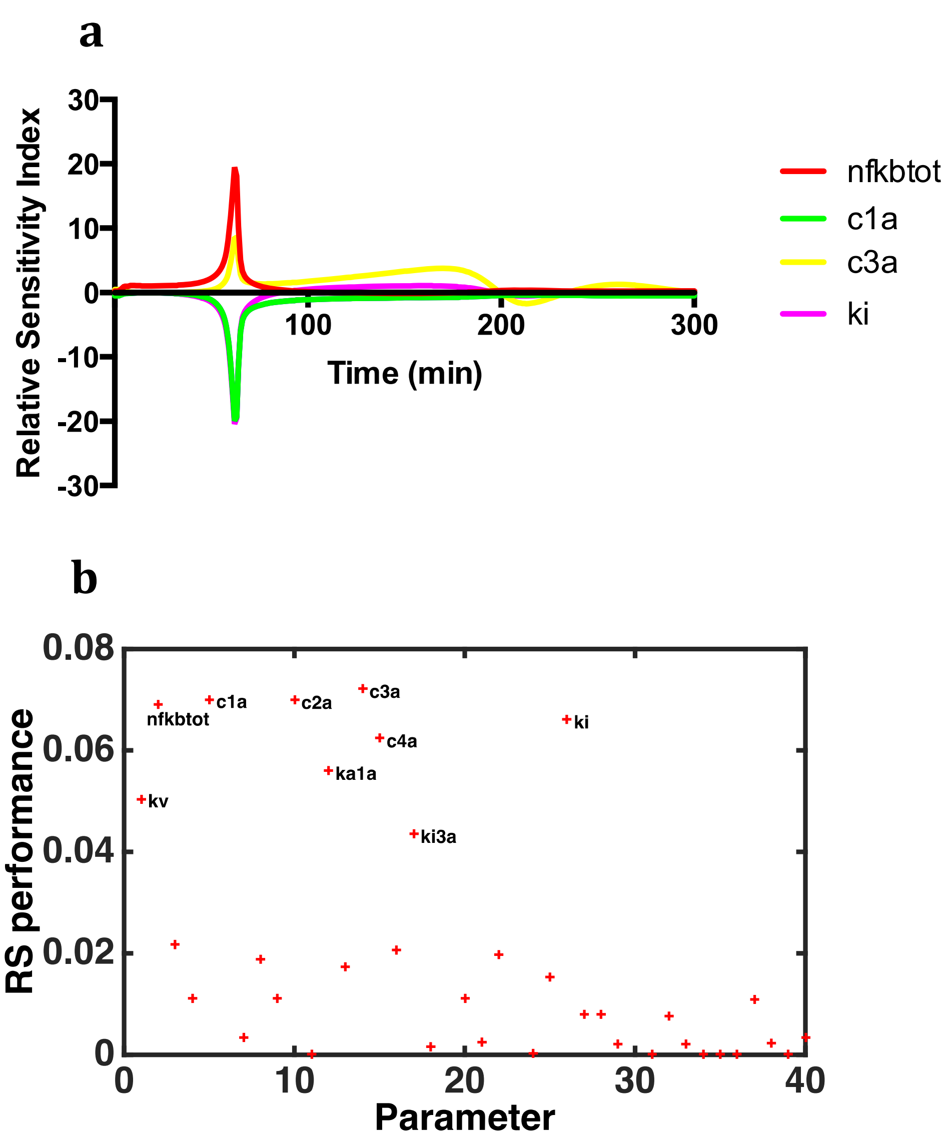

Supplementary Figure 27. Dynamic sensitivity analysis of NF-κB activation in response to a single 5 min pulse of 10 ng ml^{-1} TNFα. (a) Temporal relative sensitivity coefficients of NF-κB p65 nuclear translocation trajectory. Colour lines correspond to most sensitive model parameters, IκBα transcription, translation and mRNA half-life rate (c1a, c2a, c3a), and NF-κB level *(nfkbtot).* (b) Relative sensitivity (RS) performance with respect to nuclear NF-κB level for all model parameters (calculated as an average temporal sensitivity coefficient over the 300 min trajectory). Names of most sensitive parameters indicated on the graph.

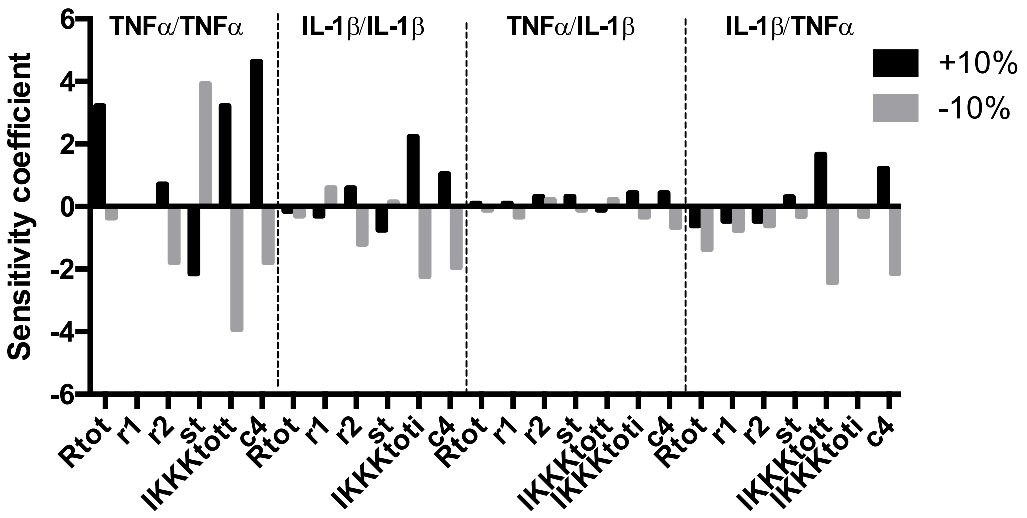

Supplementary Figure 28. Sensitivity analysis of the TNFα and IL-1β receptor modules. Shown is the comparison of sensitivity coefficient calculated with respect to receptor parameters (total TNFα and IL-1β receptors (*Rtot*), receptor activation rate/cytokine binding rate (*r1*); receptor de-activation rate (*r2*), half maximal receptor activation (st)) and IKKK-related parameters (total IKKK in TNFα and IL-1β pathway (*IKKKtott* and *IKKKtoti*, respectively) and A20 degradation rate (*c4*). Sensitivity coefficients calculated based on the fraction of 300 simulated cells responding to cytokine pulses at a 60 min interval (as indicated on the graph) with respect to 10% parameter change.

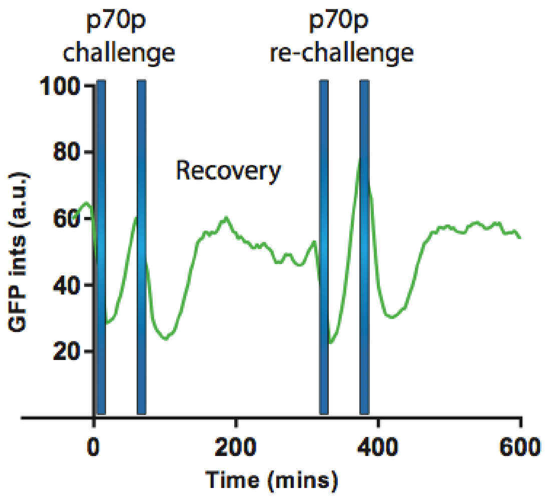

Supplementary Figure 29. Schematics of the TNFα equilibrated pulses experiment. Cells were stimulated with 5 min TNFα pulses at 70 min interval (p70p) in two sets separated by a 4h equilibration period. Shown is a normalized IκBα-eGFP trajectory of a representative single C9 cell. Timing of the TNFα pulses represented with blue bars.

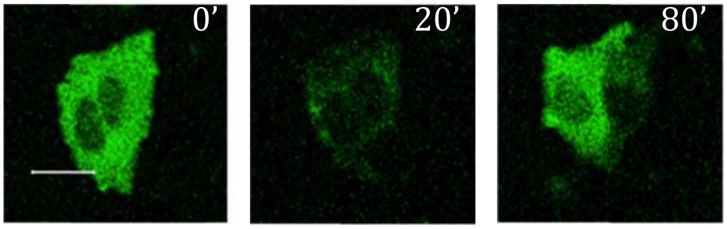

Supplementary Figure 30. Confocal microscopy images of representative daughter cell responses. Both C9 daughter cells respond to the first TNFα (depicted at 20 min after stimulation), but exhibit heterogeneous responses to the second pulse (depicted at 80 min after start of the experiment). Shown is the IκBα-eGFP fluorescence signal. Scale bar denotes 20 μm.

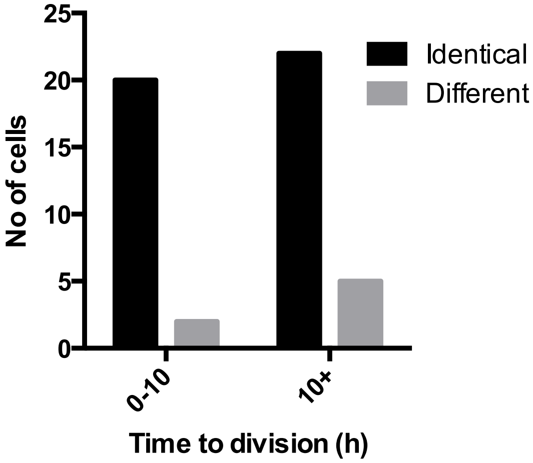

Supplementary Figure 31. Analysis of daughter cell responses. Correlation between daughter cell responses (as in Fig. 5H) and the time from division at stimulation (stratified into <10h or >10h intervals).

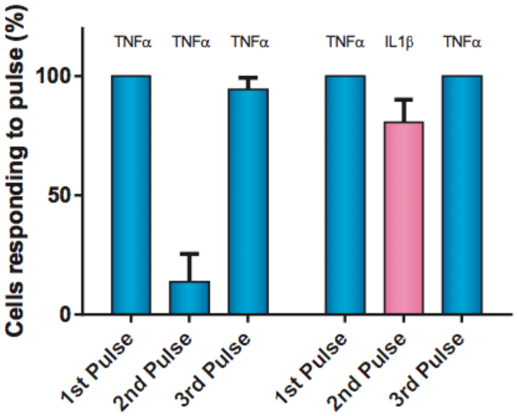

Supplementary Figure 32. Single cell responses to 50 min TNFα and IL-1β pulses. Shown is the fraction (mean ± s.d.) of responding C9 cells to respective stimulation as determined by the p65-mCherry translocation. Timing of TNFα

and IL-1β pulses represented with blue and pink bars, respectively. Data from Fig 6a.

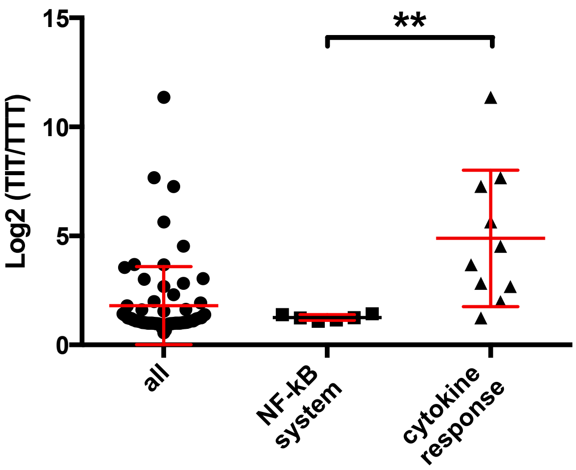

Supplementary Figure 33. Differential gene expression analysis between TNFα/IL-1β/TNFα (TIT) and TNFα/TNFα/TNFα (TTT) stimulation. Shown are log2 of expression fold changes for NF-κB system genes (A20, IκBα, IκBε, Rel, RelB NF-κB1, NF-κB2) and cytokine response genes (CXCL2, CXCL1, IL8, TNFAIP6, CSF2, TNFα, LIF, IL-1β, CCL2), in comparison to two groups merged together (all). Shown in red are respective means (± s.d.) of three replicates. "**" denotes a statistical difference (at $p<0.01$) assessed by Mann-Whitney test (see *Supplementary Data 1*).

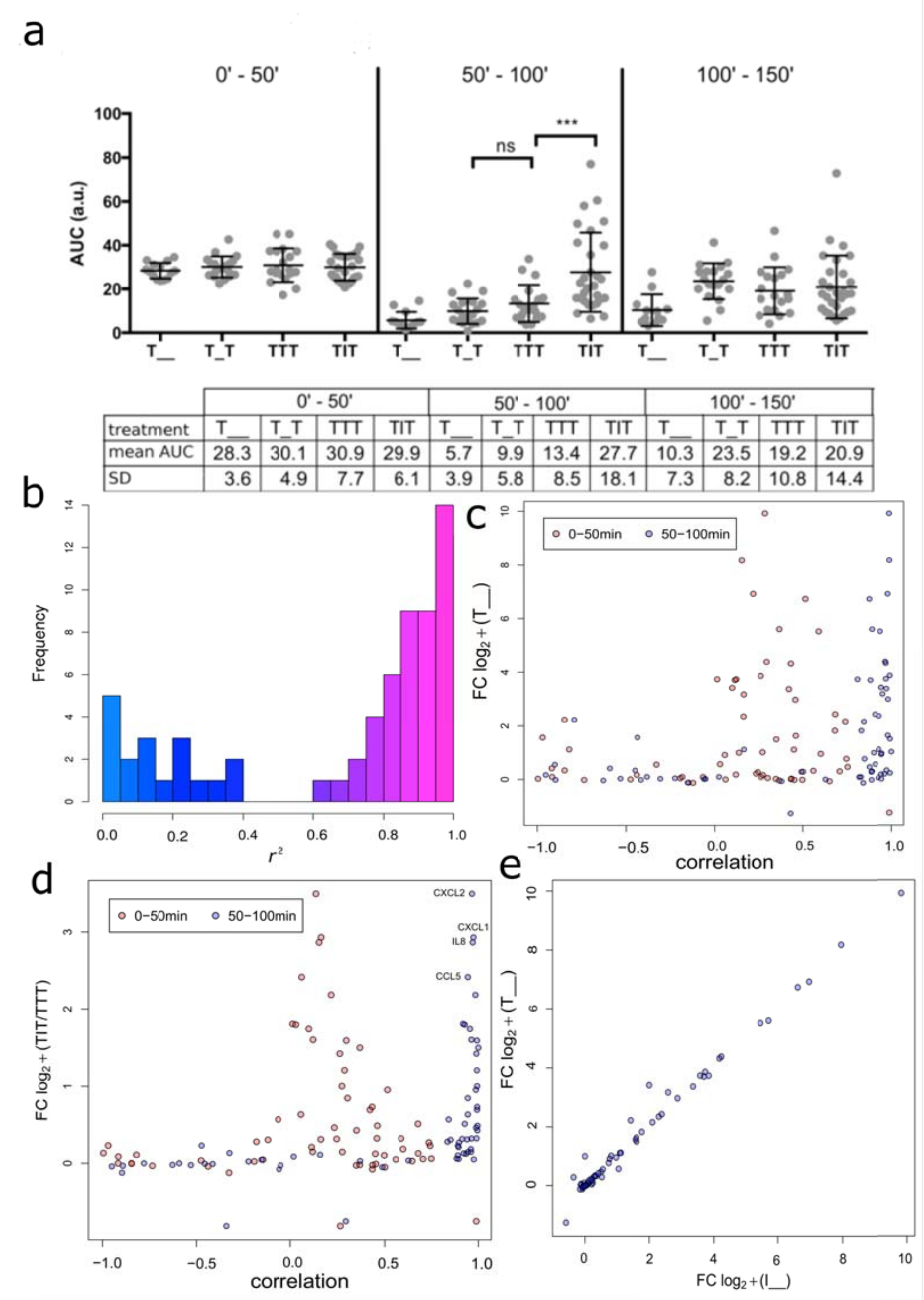

	0' - 50'				50' - 100'				100' - 150'			
treatment	T__	T_T	TTT	TIT	T__	T_T	TTT	TIT	T__	T_T	TTT	TIT
mean AUC	28.3	30.1	30.9	29.9	5.7	9.9	13.4	27.7	10.3	23.5	19.2	20.9
SD	3.6	4.9	7.7	6.1	3.9	5.8	8.5	18.1	7.3	8.2	10.8	14.4

Supplementary Figure 34. Correlation of the single cell microscopy and gene expression (Nanostring) data. (a) Area under the curve (AUC) of nuclear p65-mCherry intensity at different time intervals with varying treatment regimes (as in Fig. 6c). Shown is the mean AUC (top row) ± s.d. (bottom row) of p65-mCherry base-line corrected trajectories normalized to the first peak amplitude. Statistical significance assessed based on the Kruskal-Wallis with post-hoc Mann-Whitney comparisons test (ns-not significant, *** p value =

0.0005) (b) Histogram of the coefficient of determination (r^2, on the x-axis) for the correlation between AUC values at 50-100 minutes and corresponding gene expression levels for varying treatment regimes. (c) Relationship between the Pearson correlation (r, x-axis) calculated between mean NF-κB AUC in single cells and the gene expression patterns, and the fold-change (from untreated, y-axis) in gene expression in response to single pulse of TNFα. Shown in red is the correlation coefficient calculated for AUC between 0-50 min, and in blue for 50-100 min time interval (d) Relationship between the Pearson correlation (as in c) and the ratio of the transcript levels post TIT and TTT treatments. (e) Correlation between FC (fold-change) (from untreated) in transcript level between cells treated with a single pulse of IL-1β (I_ _) and TNFα (T_ _).

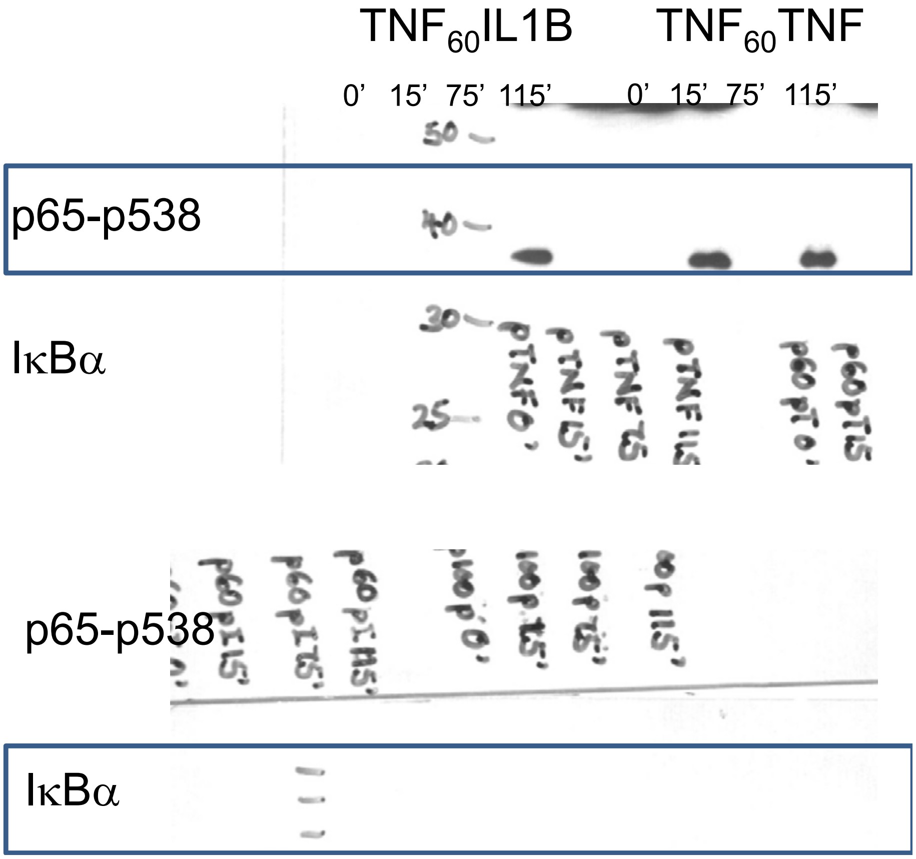

Supplementary Figure 35. Uncropped scans of the blots in Fig. 3d. Shown are the two different exposure times. Boxed bands are presented in the manuscript.

**Figure**	**Cell line**	**Protocol**	**Number of cells**	**Number of replicates**	Notes
**1F**	C9<br><br>C9L 	T Cont.<br>I Cont.<br>T Cont.<br>I Cont.	**163**<br>**94**<br>**30**<br>**47**	>3<br>3<br>3<br>3	
**1D**	C9<br>C9L	T, I Cont.	**37, 57**<br>**30, 47**	1 replicate<br>3	subset of 1F<br>from 1F
**1E**	C9L	T, I Cont.	**30, 47**	3	from 1F
**2A**	C9	T	**63 (20 shown)**	>3	
**2B,C**	C9	TT, 60 min	**113**	3	
**2F**	C9	TT, 50 min<br>TT, 60 min<br>TT, 70 min<br>TT, 80 min<br>TT, 100 min	**61**<br>**113**<br>**79**<br>**63**<br>**57**	2<br>3<br>2<br>2<br>2	 From 2B,C
**2I**	C9L	TT, 60 min<br>TT, 100 min	**36**<br>**19**	2<br>2	
**2J**	C9L	T Cont.<br>TT, 60 min<br>TT, 100 min	**30**<br>**12**<br>**19**	 2<br>2	from 1D<br>subset of 2I<br>2I
**S. 9**	C9	I<br>I pulse<br>II, 60 min<br>IT, 60 min	**35**<br>**37**<br>**39**<br>**42**	2<br>2<br>2<br>2	with IL-1 anti-body
**3A**	C9	T I, 60 min	**95**	3	
**3B**	C9	TI, 60 min<br>TT, 60 min	**95**<br>**115**	3<br>3	From 3B<br>From 2F
**3C**	C9L	TT, 60 min<br>TI, 60 min	**36**<br>**47**	2<br>3	From 2I
**4H**	C9	TT, 60 min, A20 siRNA<br>TT, 60 min, neg. siRNA	**78**<br>**35**	4<br>2	
**5D**	C9	TTTT (Equilibrated)	**79**	2	
**5J**	C9	TT, 70 min	**112 (56 pairs)**	2	
**6B**	C9	TTT, 50 min<br>TIT, 50 min	**19**<br>**29**	3<br>3	
**S. 13**	C9	T pulse	**25**	2	Dynasore
**S. 18**	C9	TT, 60 min<br><br>TT, 60 min	**78**<br><br>**30**	2<br><br>2	Low dose<br><br>High dose
**S. 34**	C9L	T _ _ , 50 min<br>T_T, 50 min<br>TTT, 50 min<br>TIT, 50 min	**14**<br>**19**<br>**19**<br>**29**	2<br>2<br>3<br>3	 From 2I<br>From 6B<br>From 6B

Supplementary Table 1. Imaging conditions and umber of cells used in the study. T and I in the simulation protocol refer to TNFα and IL-1β treatments, respectively.

Variable	Description	Variable	Description
TNF	Extracellular TNFα	IL1	Extracellular IL-1β
Rnt	Neutral TNFR (receptor)	Rni	Neutral IL1R (receptor)
Rt	Active TNFR (receptor)	Ri	Active IL1R (receptor)
$IKKKn_t$	Neutral form of IKKK (TNFR pathway)	$IKKKn_i$	Neutral form of IKKK (IL1R pathway)
$IKKKa_t$	Active form of IKKK (TNFR pathway)	$IKKKa_i$	Active form of IKKK (IL1R pathway)
$IKKKi_t$	Inactive form of IKKK (TNFR pathway)	$IKKKi_i$	Inactive form of IKKK (IL1R pathway)
IKKn	Neutral form of IKK	IKKa	Active form of IKK
IKKi	Inactive form of IKK	NFκB	Free cytoplasmic NFκB protein
nNFκB	Free nuclear NF-κB	IκBα	Free cytoplasmic IκBα
nIκBα	Free nuclear IκBα	tIκBα	IκBα transcript
A20	Cytoplasmic A20 protein	tA20	A20 transcript
NFκBIκBα	Cytoplasmic NF-κB IκBα complex	nNFκBIκBα	Nuclear NF-κB IκBα complex
pNFκBIκBα	Phospho-IκBα:NF-κB complex		

Supplementary Table 2. Description of NF-κB model variables. Variables are quantified in the number of molecules. Letter *n* denotes nuclear variable, *t* denotes mRNA transcript, while *p* denotes phosphorylated form of IκBα.

1. ddt(NF-κB) = kd1a*IκBαNFκB - ka1a*IκBα*NFκB - ki1*NFκB + kv*ke1*nNFκB + kt2a*pIκBαNFκB

2. ddt(IκBα) = kd1a*IκBαNFκB - ka1a*IκBα*NFκB - ki3a*IκBα + kv*ke3a*nIκBα - c4a*IκBα + c2a*tIκBα - kc1a*IKK*IκBα

3. ddt(IκBαNFκB) = ka1a*IκBα*NFκB - kd1a*IκBαNFκB + kv*ke2a*nIκBαNFκB - kc2a*IKKa*IκBαNFκB

4. ddt(nNFκB) = kd1a*nIκBαNFκB - ka1a*kv*nIκBα*nNFκB + ki1*NFκB - kv*ke1*nNFκB

5. ddt(nIκBα) = kd1a*nIκBαNFκB - ka1a*kv*nIκBα*nNFκB + ki3a*IκBα - kv*ke3a*nIκBα - c4a*nIκBα

6. ddt(nIκBαnNFκB) = ka1a*kv*nIκBα*nNFκB - kd1a*nIκBαNFκB - kv*ke2a*nIκBαNFκB

7. ddt(tIκBα) = c1a*(nNFκB^h/(nNFκB^h + k^h))- c3a*tIκBα

8. ddt(IKKn) = kp*(ikktot-IKKa-IKKn) - (IKKKat^ha/(IKKKat^ha + (sIKKK)^ha))*ka*IKKn - (IKKKai^ha/(IKKKai^ha + (sIKKK)^ha))*ka*IKKn

9. ddt(IKK) = (IKKKat^ha/(IKKKat^ha + (sIKKK)^ha))*ka*IKKn -ki*IKK + (IKKKai^ha/(IKKKai^ha + (sIKKK)^ha))*ka*IKKn

10. ddt(tA20)= c1*(nNFκB^h/ nNFκB^h + k^h) – c3*tA20

11. ddt(A20)= c2*tA20 - c4*A20

12. ddt(IKKKtn) = m3*(IKKKtott-IKKKat-IKKKnt) - (Rt/(Rt + st))*m1*(IKKKtn)

13. ddt(IKKKt) = (Rt/(Rt + st))*m1*(IKKKnt) - (m2+ka20*A20)*IKKKat;

14. ddt(Rt)= q1*TNF*(Rtot - Rt)-q2*Rt;

15. ddt(TNF)= -c5*TNF - q1*TNF *(Rtot - Rt)

16. ddt(IKKKin) = m3*(IKKKtoti-IKKKai-IKKKni) - (Ri/(Ri + st))*m1*(IKKKni)

17. ddt(IKKKi) = (Ri/(Ri + st))*m1*(IKKKni) - (m2+ka20*A20)*IKKKai

18. ddt(Ri)= q1*IL1*(Rtot - Ri)-q2*Ri

19. ddt(IL1)= -c5*IL1 -q1*IL1*(Rtot - Ri)

Supplementary Table 3. Rate-equations for the deterministic mathematical model of NF-κB signalling. *ddt(X)* denotes the derivative of X with respect to time.

Parameter	Symbol	Value	Ashall et al., 2010	Reference
Spatial Parameters				
Cytoplasmic/nuclear ratio	kv	3.3	3,3	As in [1]
Initial Concentration				
Total NF-κB	nfkbtot	10^5 molecules	10^5 molecules	As in [1]
Total IKK	ikktot	10^5 molecules	10^5 molecules	As in [1]
Total IKKKt	IKKKtott	10^5 molecules	-	Fitted
Total IKKKi	IKKKtoti	1.3×10^5 molecules	-	Fitted
IKK				
IKKn → IKKa<br>*Order of hill function, ha=3*<br>*Half-max constant, sIKK=350 (fitted)*	*ka*	0.0014 s^{-1}	0.003 s^{-1}	Re-fitted
IKKa → IKKi	*ki*	0.002 s^{-1}	0.002 s^{-1}	As in [1]
IKKi → IKKn	*kp*	0.00033 s^{-1}	-	Fitted
IKKK				
IKKKn → IKKKa<br>*Order of hill function, ha=3*<br>*Half-max constant, st=50 (fitted)*	*m1*	0.005 s^{-1}	-	Fitted
IKKKa → IKKKi	m2	0.0005 s^{-1}	-	Fitted
IKKKi → IKKKn	*m3*	0.0005 s^{-1}	-	Fitted
IKKKa →IKKKi (A20 mediated)	*ka20*	0.00023 s^{-1}	-	Fitted
Complex formation and dissociation				
IκBα + NF-κB → IκBα·NF-κB	*ka1a*	8×10^{-7} s^{-1}	4×10^{-7} s^{-1}	Re-fitted
IκBα·NF-κB → IκBα + NF-κB	*kd1a*	8×10^{-4} s^{-1}	5×10^{-4} s^{-1}	Re-fitted
IκBα protein synthesis and degradation				
nNF-κB → nNF-κB + tIκBα<br>*Order of hill function, h=2*<br>*Half-max constant, k=0.002 (fitted)*	*c1a*	0.2 s^{-1}	0.08 s^{-1}	Re-fitted
tIκBα→ tIκBα + IκBα	*c2a*	0.5 s^{-1}	0.5 s^{-1}	As in [1]
tIκBα→ Sink	*c3a*	0.00044 s^{-1}	0.0003 s^{-1}	Re-fitted
IκBα→ Sink	*c4a*	0.00076 s^{-1}	0.0005 s^{-1}	Re-fitted
A20 Protein synthesis and degradation				
nNF-κB → nNF-κB + tA20<br>*Order of hill function, h=2*<br>*Half-max constant, k=0.002 (fitted)*	*c1*	0.005 s^{-1}	0.0873 s^{-1}	assumed
tA20→ tA20 + A20	*c2*	0.5 s^{-1}	0.5 s^{-1}	As in [1]
tA20→ Sink	*c3*	0.00066 s^{-1}	0.00048 s^{-1}	Re-fitted
A20→ Sink	*c4*	0.00075 s^{-1}	0.0045 s^{-1}	Re-fitted
Transport				
NF-κB → nNF-κB	*ki1*	0.0026 s^{-1}	0.0026 s^{-1}	As in [1]
nNF-κB → NF-κB	*ke1*	0.00052 s^{-1}	0.00017 s^{-1}	ki1/50 [5]
nIκBα·nNF-κB → IκBα·NF-κB	*ke2a*	0.01 s^{-1}	0.01 s^{-1}	As in [1]
IκBα → nIκBα	*ki3a*	0.001 s^{-1}	0.002 s^{-1}	Re-fitted
nIκBα → IκBα	*ke3a*	0.0005 s^{-1}	0.01 s^{-1}	ki3a/2 [5]
Base Module IKK Interaction Parameters				
IKKa + IκBα → pIκBα	*kc1a*	9.8×10^{-8} s^{-1}	2.96×10^{-7} s^{-1}	Re-fitted
IKKa + IκBα·NF-κB → pIκBα·NF-κB	*kc2a*	8.5×10^{-7} s^{-1}	2.96×10^{-7} s^{-1}	Re-fitted
pIκBα.NF-κB →Sink	*kt2a*	0.1 s^{-1}	0.1 s^{-1}	As in [1]
Receptor Module				
Total receptor	*Rtot*	2500	-	assumed
Rn+ TNFα →Rt<br>(the same assumed for IL-1R)	*r1*	4×10^{-9} s^{-1}	-	assumed
Rt → Rn<br>(the same assumed for IL-1R)	*r2*	0.0032 s^{-1}	-	assumed
TNF/IL1 →sink	*c5*	0.000037 s^{-1}	-	assumed

Supplementary Table 4. Parameter values for the deterministic NF-κB model.

Parameter	Symbol	Value	Reference
nNF-κB+GIκBα(0)→ nNF-κB + GIκBα(1)	q1a	4×10^{-7} s^{-1}	[6]
nNF-κB+GA20(0)→ nNF-κB + GA20(1)	q1	4×10^{-8} s^{-1}	Assumed
nIκBα +GIκBα(1)→ nIκBα + GIκBα(0)	q2a	10^{-6} s^{-1}	[6]
nIκBα +GA20(1)→ nIκBα + GA20(0)	q2	10^{-6} s^{-1}	[6]
nNF-κB → nNF-κB + tIκBα	c1a*	c1a/2	Assumed
GA20(1) → GA20(1) + tA20	c1*	c1/2	Assumed
tA20 → sink	c3a	c3a	As in deterministic model
Rn + TNFα → Rt	*r1*	4×10^{-9} s^{-1}	Adapted from [6]
Rt →Rn	*r2*	0.0032 s^{-1}	Fitted

Supplementary Table 5. Parameter values for the stochastic NF-κB model.

Figure	Model simulation	Parameters varied
4B	Deterministic	Total $IKKK_t$ level varied between 0 – $15 x 10^4$ and total A20 protein level varied between 0 – 4000 molecules<br>Single cell trajectories simulated with total $IKKK_t$ = 140,000 and 60,000 molecules
4C,D,E	Deterministic	Total $IKKK_t \sim N(100000, 30000^2)$
4F	Deterministic	Total $IKKK_t \sim N$(100000, specified in figure)
4G	Deterministic	All parameters changed to 50 or 150% of their respective nominal values
4I	Deterministic	Total $IKKK_t \sim N(100000, 30000^2)$<br>m3~N($6x10^{-4}$, $3.2x10^{-8}$)<br>c4a ~$N(6x10^{-4}, 5x10^{-8})$
5B,C,E	Deterministic[1] and stochastic gene expression	[1] Total $IKKK_t \sim N(100000, 30000^2)$
S. 14<br>S. 15<br>S. 16<br>S. 17<br>S. 19<br>S. 20<br>S. 22	Deterministic	Total $IKKK_t \sim N(100000, 30000^2)$
S. 18	Deterministic[1] and Stochastic receptor	[1] Total $IKKK_t \sim N(100000, 30000^2)$
S. 21	Deterministic	Total IKKK level varied between 0 – $15x10^4$ and total IκBα protein level between 0 – $10x10^4$ molecules
S. 23	Deterministic	Total IKKK (specified in figure)
S. 24	Deterministic	All parameters changed to 50 or 150% of their respective nominal values
S. 25<br>S. 26<br>S. 27	Deterministic	Responding and non-responding cell simulated with total $IKKK_t$ equal to 1.5 x 10^5 and 7 x 10^4 molecules, respectively.
S. 28	Deterministic	Total $IKKK_t \sim N(100000, 30000^2)$

Supplementary Table 6. Models and parameters used for specific simulations performed in the study.

Supplementary Note 1: Derivation and validation of stable cell lines.

Generation of the IκBα-EGFP BAC

The IκBα BAC (CTD-3214F11, obtained from Life Technologies, UK) was maxi-prepped using Machnery-Nagal Nucleobond BAC100 kit and 100ng was used to transform SW102 *E. coli* cells (a kind gift from Neal Copeland) by electroporation (1.8kV, 200 ohm, 25mF). SW102-IκBα positive clones were selected with by Southern blot and pulsed field gel electrophoresis analysis.

For recombination, the 300bp immediately 5' of the IκBα stop codon were amplified by the primers *IκBα 5'300H KpnI F* ACACTA*GGTACC*CTTATCAGAGGGGTATCTACATAATGAGTCTCTCAAA and *IκBα 5'300H SalI R* GGAACA*GTCGAC*TAACGTCAGACGCTGGCCTCCA. The 350bp immediately 3' of the IκBα stop codon were amplified by the primers *IκBα 3'350H BamHI F* ATAATA*GGATCC*GCGCAAAGGGGCTGAAAGAACATG and *IκBα 3'350H XbaI R* AATCAA*TCTAGA*ACAGGATACCACTGGGGTCAGTCA. The homology arms were sequentially cloned into the pBlueGalK vector either side of the EM7-GalK coding sequence via the restriction tags, first generating pBlueGalK-IκBα^{FP}-3' then pBlueGalK-IκBα^{FP}-5'/3'. The eGFP coding sequence was amplified with the primers *eGFP EcoRI F* GAGTCC*GAATTC*ATGGTGAGCAAGGGCGAGGAG and *eGFP BamHI R* TTCTGA*GGATCC*CTACACATTGATCCTAGCAGAAGCACAGG and cloned into the pBlueGalK-IκBα^{FP}-5'/3' vector via the EcoRI and BamHI restriction sites to give pBlueGFP-IκBα^{FP}-5'/3'.

The pBlueGalK-IκBα^{FP}-5'/3' and pBlueGFP-IκBα^{FP}-5'/3' vectors were used as a template in a PCR with the primers *IκBα RecCas Amp F* GCTTATCAGAGGGGTATCTACATAATGAGTCTCTC and *IκBα RecCas Amp R* GATACCACTGGGGTCAGTCACTCGA to generate both the H-GalK-H recombination cassette (1.9kb) and the H-GFP-H recombination cassette (1.5kb). These recombination cassettes were used in the primary and secondary targeting steps respectively using protocols described in [7]. Briefly, 1 ml of an overnight culture was diluted in 50ml Luria–Bertani (LB) medium with chloramphenicol selection (12.5 mg ml^{-1}) in a 50 ml baffled conical flask and grown at 32°C in a shaking

incubator to an OD_{600} of 0.6. Then, 15 ml was transferred to another baffled 50 ml conical flask and heat-shocked at 42C for exactly 15 min by swirling in a water bath. The remaining culture was left at 32C as the un-induced control. The two samples were then transferred to precooled 50 ml Falcon tubes and pelleted using 5000 r.p.m. at 0°C for 5 min. The supernatant was poured off and the pellet was suspended in 10 ml ice-cold ddH2O and the samples pelleted again. This step was repeated once more. After the second washing and centrifugation step, the supernatant was removed, and the pellet suspended in 1 ml ice-cold ddH2O and spun at full speed in a pre-chilled minicentrifuge, with two more 1 ml washes. The final pellet was suspended in 50ul ice-cold ddH2O and mixed with 100ng of recombination cassette and electroporated in a 0.1 cm cuvette (Geneflow) at 25 mF, 1.75 kV and 200 V. After electroporation the bacteria were recovered in 1 ml LB for 1 h in a 32°C shaking incubator. For the secondary targeting step, the bacteria were recovered in 10 ml LB in a 50 ml baffled conical flask and incubated for 4.5 h in a 32°C shaking incubator. After the recovery period, the bacteria were washed three times in 1x M9 salts as follows: 1 ml culture was pelleted in an eppendorf tube at 13 200 r.p.m. for 15 s and the supernatant was removed with a pipette. The pellet was suspended in 1 ml of 1xM9 salts, and pelleted again. This washing step was repeated once more. After the second wash, the supernatant was removed and the pellet was suspended in 1 ml of 1xM9 salts before plating on minimal medium plates [7].

Colonies were screened by Pulsed Field Gel Electrophoresis for clones that cut with an additional SalI site (inserted between 5' Homology arm and GFP sequence). A single positive clone was detected from ~80 screened. Sequencing confirmed the correct recombination.

Retrofitting BAC with a mammalian selection marker

To generate stable cell lines it was necessary to retrofit the IκBα-EGFP BAC with an appropriate mammalian selection marker. We developed retrofitting constructs that could universally be applied to any BAC. Briefly, due to derivation from the same parent BAC vector construct, pBAC108L, alignment of the most common BAC vectors, pe3.6 (from the Roslin Park institute library) and the pBeloBAC vector (from the California Institute of Technology) showed perfect

homology in approximately 6kb of construct. Within this homologous region the chloramphenicol resistance gene was identified as a suitable target for replacement with a new selection marker as this would not disrupt any important bacterial sequences. Restriction site tagged homology arms 300-400bp in length were amplified from the BAC sequence using the primers (underlined indicates enzyme site) 5'H KpnI F tgtcaaGGTACCGGCAGCCACATCCAG, 5'H EcoRI R ggtgccGAATTCTCAACGTCTCATTTTCGC, 3'H BamHI F aatgggGGATCCTGGACAACTTCTTCGCC, 3'H SacII R aatgggCCGCGGGCCGTCGACCAATTCTC and cloned using the appropriate enzymes into the multiple cloning sites of pL451 [8]. This resulted in a recombination cassette containing H-pGK-pEM7-Kan/Neo-H. Retrofitting was performed in the same SW102 strain hosting the BAC by heat inducing the bacteria for recombination, transforming with the cassette and plating on LB containing Kanamycin (25 ug ml^{-1}). Clones were screened by PFGE and >90% recombination efficiency was observed.

BAC Stable transfection

BAC DNA was prepared by maxiprep (BAC100 Nucleobond kit, Macherey-Nagel, Germany) and 1 ug or 3 ug were used to transfect 10^6 SK-N-AS cells in a 10 cm dish using 9.87 ul ExGen500 transfection reagent (following manufacturer's recommendations). Media was changed 3 days post transfection and supplemented with 500 μg ml^{-1} G418. Media + antibiotic were refreshed every 3-4 days. Colonies formed 2-3 weeks after culturing in selection containing media were ring cloned into individual wells of a 48 well plate and sequentially scaled up to large culture vessels as necessary.

Analysis of IκBα and NF-κB p65 levels in BAC cell lines.

The temporal NF-κB response was investigated in developed BAC IκBα-eGFP cell lines. In C9 cells, which were used for most of the single cell experiments, TNFα stimulation resulted in a rapid degradation and re-synthesis of IκBα protein, similar to that in WT cells (Supplementary Figure 1). At 15 min after treatment, the endogenous IκBα protein was undetectable, which was potentially

consequence of low resting levels, while a residual expression of IκBα-eGFP was present. A simultaneous recovery of the endogenous and IκBα-eGFP protein was observed, with a peak at 60 min (after continuous TNFα stimulation). Further accumulation of IκBα levels was observed 120 min after a single TNFα pulse, but not after continuous stimulation, reaching pre-stimulation steady-state (i.e., high IκBα-eGFP and low endogenous IκBα levels). This behaviour was in a good agreement with single-cell data in Fig. 2a. In both cases the fluorescently labelled protein mirrored the response of the endogenous IκBα. A delayed degradation of IκBα-eGFP was observed in C9L, comparing to that in C9 cells, Figure 2d and Supplementary Figure 1, while the endogenous IκBα in C9L showed a rapid and complete degradation at 15 min after stimulation, similar to that of WT cells. A comparative analysis of IκBα in different cell lines showed that the resting endogenous IκBα levels we elevated in C9L, comparing to C9 cells (Supplementary Figure 2). We expect that this be due to transduction of fluorescently labelled p65-mCHerry, which might enhance production of or stabilise IκBα protein [1,9].

We found that NF-κB p65 oscillation patterns in our stable cell lines were consistent (period of 106 ±30 and 109 ±27 min in C9 and C9L, respectively, as defined by individual peak-to-peak timing of IκBα-EGFP, **Supplementary Figure 3**). Similarly, SK-N-AS cells transiently transfected with IκBα-eGFP BAC showed a similar period of 116 ±27 min. Also, C9 cells transfected with p65-Dsredxp plasmid a period of 110 ±21 min, while the period in WT SK-N-AS cells transfected with the same plasmid was 110 ±38 min. These data confirmed that our cell lines provide a good model system studying single cell NF-κB responses.

Often the IκBα-eGFP fluorescence intensity remains below its initial value as it oscillates (Fig. 1c). We expect that this effect could be due (at least in part) to the time taken for folding and maturation of the fluorophore [10,11], despite IκBα-eGFP protein level reaching the pre-stimulated steady state (or higher) at 60 min after treatment (Supplementary Figure 1). Thus, only a proportion of stimulus-induced IκBα-eGFP molecules might be detected in the microscopy experiment and this would be lower at higher oscillation frequencies. In addition we observe

that C9L cell line is characterised by lower amplitude of IκBα-eGFP oscillations (comparing with C9 cells, Fig. 1c). We expect that this could be due elevated IκBα-eGFP levels in C9L cells, which could lead to more damped oscillation patterns. Damped oscillations at the single cell level are consistent with partial IκBα degradation observed in the population (Supplementary Figure 1).

IκBα and p65 oscillate out of phase.

In response to continuous TNFα stimulation C9L cells exhibited out-of-phase oscillations in the IκBα-eGFP level and the p65-mCherry nuclear translocation (Fig. 1c and Supplementary Figure 4a, for representative single cell traces). Cross-correlation analysis of representative cells showed about 50 min time lag between the IκBα-EGFP and p65-mCherry signals (Supplementary Figure 4b). The overlapping peaks in the power spectra highlighted a common oscillation frequency of IκBα-eGFP and p65-mCherry in single cells (Supplementary Figure 4c, at around 0.01 min^{-1}, which corresponds to about 100 min period). The frequencies of IκBα-eGFP and p65-mCherry oscillation were tightly correlated, while the lag time was inversely proportional to the oscillation frequency in single cells (Supplementary Figure 4d, e).

Supplementary Note 2: Single cell responses to TNFα and IL-1β pulses

C9 cells were stimulated with two 5 min TNFα pulses at intervals ranging from 50 to 100 min (Fig. 2e). The average IκBα-eGFP traces corresponding to the "responding" and "non-responding" clusters at 60 min interval are shown in Supplementary Figure 6. Single cell IκBα-eGFP trajectories are depicted with heat maps (see Fig. 2c for 60 min, and Supplementary Figure 7 for 50, 70, 80 and 100 min pulse interval). Cells were then classified as "*responders*" if a gradient of the corresponding IκBα-eGFP trajectory at the time of stimulation was not positive; otherwise they were called "*non-responders*".

Unsupervised k-means clustering was used to validate this classification (Supplementary Figure 8). Cells were first clustered into different number of groups varying from two to four. The previously inferred "*responding*" and "*non-responding*" cells (based on the IκBα-eGFP gradient) were mapped on to obtained clusters (depicted in green and blue respectively). We found that overall the gradient-based classification was recapitulated very well in the k-means clusters (with exception of the 50 min pulsing). The Silhouette coefficient could be used to find the optimal number of clusters, however in practice it would be more difficult to implement. For example, 2 optimal clusters were identified in the 60 min pulse interval data (indicated by the highest Silhouette coefficient, in comparison with the coefficients obtained for three and four clusters, respectively), while three of four clusters separated the "*responding*" and "*non-responding*" cells better. Based on this analysis, we therefore applied a method based on the IκBα-eGFP gradient in this manuscript.

In order to test whether multiple refractory states exist within NF-κB system C9 cells were stimulated with two pulses of IL-1β. 76% of cells responded to the second pulse (**Supplementary Figure 9**), suggesting existence of a similar stimulus specific refractory state to that seen with TNFα. All the cells stimulated with a pulse of TNFα responded to the pulse of IL-1β 60 min later, suggesting that the refractory period to TNFα stimulation might be controlled upstream of IKK in the signal transduction (Fig. 3a-d). When a TNFα pulse was applied after

IL-1β stimulation, 54% of cells responded, **Supplementary Figure 9** (in comparison almost ~100% of cells responded to a IL-1β pulse 60 min after stimulation with TNFα, Fig. 3a). This suggested some influence of the IL-1β transduction pathway on TNFα signalling.

Supplementary Note 3: Analysis of TNFα internalization.

Cells were stimulated with 5 min pulses of FITC and Tx-Red fluorescently labelled TNFα at a 60 min interval (Figs 2e-f). Confocal microscopy images of representative WT cell stimulated first with FITC- and then Tx-Red-labelled TNFα showed that internalization pattern was not affected (Supplementary Figure 10). As a control, the cells were stimulated first with Tx-Red and then FITC-labelled TNFα, showing no change in internalization kinetics (Supplementary Figure 11). We noticed in Fig 1g the levels of TNFα-FITC fluorescence decreased over time, while the levels of TNFα-TxRed increased in the corresponding experiment (Supplementary Figure 11). We tested if the decrease of FITC (but not in the Tx-Red) fluorescence was due to its sensitivity to low-PH environment [12]. We excited TNFα-FITC at 488 and 458 nm wavelength, however saw no change in the fluorescence levels over time (Supplementary Figure 12). This suggested that changes in TNFα-FITC and Tx-Red over time were due to their fluorescent properties [11].

Treatment with endocytosis inhibitor dynasore [13] prevented internalisation of labelled TNFα, however, no change in NF-κB activation was observed in single cells (Supplementary Figure 13). Functionality of the dynasore was confirmed with an acid wash. This suggested that endocytosis did not affect NF-κB system responses.

Supplementary Note 4: Correlation between single cell responses and gene expression

Cells were stimulated with three pulses of TNFα, all single cells responded to the 1st and 3rd pulse (at 0 and 100 min, respectively), but were refractory to the 2nd pulse applied at 50 min. In contrast, when stimulated with IL-1β at 50 min most of the cells exhibited a corresponding nuclear p65-mCherry translocation (Supplementary Figure 32). The comparison between TNFα/IL-1β/TNFα (TIT) and TNFα/TNFα/TNFα (TTT) stimulated genes (as assessed by Nanostring assay, Fig. 6c), showed a panel of 26 genes that was differentially regulated by IL-1β at a time when cells were refractory to TNFα (Fig. 6e). In particular, the members of the NF-κB signalling system and a number of pro-inflammatory signalling molecules including cytokines and chemokines were differentially regulated (Supplementary Figure 33).

We asked if the differential response to IL-1β and TNFα pulsing (Supplementary Figure 33) could be explained by the patterns of nuclear NF-κB in single cells. We quantified the level of nuclear p65-mCherry in C9L cells stimulated as in Fig. 6c (for all conditions except of the single IL-1β pulse). In agreement with Fig. 6a-b, the differences in the temporal NF-κB kinetics were recapitulated by the area under the curve (AUC) (Supplementary Figure 34a). In particular, the AUC calculated for T_T and TTT calculated for the 50-100 min interval was significantly lower than for TIT (mean of 9.9 and 13.4 vs. 27.7), whereas there was no difference between treatments for the 0-50 and 100-150 min interval (see Supplementary Data 1 for statistical analyses). Coefficient of determination (r^2) calculated between the AUC and gene expression patterns showed a bimodal distribution (Supplementary Figure 34b, see also Supplementary Data 1 for specific values). High correlation (r^2 >0.6) suggested that patterns observed for many genes could be explained by the AUC values for respective treatments (in terms of the total variance). Those highly correlated genes were in fact up-regulated by a single TNFα pulse (T _ _ , as showed by a high Pearson correlation coefficient, r, for 50-100, but not 0-50min interval, Supplementary Figure 34c). A similar correlation structure was seen for the fold ratio between TIT and TTT

treatments (as in Fig. 6f and Supplementary Figure 34d), with genes differentially regulated by the TIT showing the highest correlation with the AUC. In agreement, a marked correlation was found between the gene expression levels in cells stimulated with single TNFα and IL-1β pulse (Supplementary Figure 34e). This analysis suggested that expression of genes differentially regulated by TNFα and IL-1β pulses could be explained in part by the patterns of nuclear NF-κB responses in single cells.

Supplementary Note 5: Mathematical model of NF-κB signalling

1. Model development

To theoretically investigate the encoding of pulse TNFα stimulation in the NF-κB system, the structure [6] and parameters of our previously developed model of the system was used [1,9,14]. The revised model was fitted manually and validated against the following single-cell and population level data (see Supplementary Figure 14 - Supplementary Figure 20 for fitted model outputs):

- Fraction (and NF-κB p65 amplitude) of cells responding to 10 ng ml^{-1} TNFα pulses at different intervals in SK-N-AS cells (data from Fig. 2).
- Single-cell NF-κB responses to IL-1β and alternate TNFα and IL-1β pulses in SK-N-AS cells (data from Figs 3, 6 and **Supplementary Figure 9**).
- Single-cell NF-κB responses to different doses of TNFα stimulation in SK-N-AS cells (data from Supplementary Figure 18).
- Single cell NF-κB p65 dynamics after continuous and repeat pulse stimulation with 10 ng ml^{-1} TNFα (and IL-1β) in SK-N-AS cells (Fig. 1), also in [1,15]
- Population-level IKK and NF-κB activity in WT and A20 knock-out (KO) mice fibroblast cells, in response to continuous TNFα treatment [2,16].
- Population-level transient IKK activity (and corresponding NF-κB activation kinetics) in response to a single pulse of TNFα of different duration [3,4].

Model parameters were fitted manually based on the parameters that were specifically developed for the SK-N-AS cell line [1]. We noticed that in the fitted model, parameters corresponding to the rate of A20-mediated inhibition of IKK (a reaction assumed in previous models [6,9,17]) was negligibly small (had no effect on systems behaviour). Therefore we removed this reaction from the model's structure arriving at the final model structure as presented in Fig. 4a.

The developed model consists of 19 ordinary differential equations and 41 parameters describing formation of complexes and their degradation, transport between nucleus and cytoplasm, transcription, translation and regulation of gene

activity (see Supplementary Table 2 for description of model variables, Supplementary Table 3 for equations and Supplementary Table 4 for parameter values). The heterogonous NF-κB responses were recapitulated in the model by distributions of parameters related to TNFα (IL-1β) signal transduction pathway (Fig. 4d).

The effects of stochastic TNFα binding and feedback gene transcription were investigated in respective stochastic formulations of the model (see Supplementary Table 5). All simulation conditions used in the study are given in Supplementary Table 6.

2. Model validation

The model recapitulated single cell NF-κB oscillatory responses (period of ~100 min) to continuous TNFα and IL-1β stimulation (Supplementary Figure 14). Simulated single cell traces appeared to be quite synchronized, suggesting that distribution of the IKKK had a limited effect on generation of heterogeneous NF-κB oscillations in response to continuous stimulation. The responses to pulses of TNFα (in terms of fraction of cells and the NF-κB amplitude) depended on the recycling of the generic IκB kinase kinase (IKKK) (A20-mediated), and specifically level of the neutral IKKK at the second pulse (Supplementary Figure 15). As described previously [1], the system exhibited full responses when pulsed at 200 min interval. The TNFα and IL-1β pulses were fitted by assuming that TNFα and IL-1β signals converge independently on the IKK, with the stimulus-specific IKKKs (Supplementary Figure 16). The increased cell responsiveness to IL-1β stimulation (75% vs. 30% for TNFα pulses) was fitted assuming a higher IKKK level in comparison to that of the TNFα transduction pathway. The alternate TNFα and IL-1β pulses at 60 min interval were fitted assuming differential A20 inhibition of the respective IKKK activation (Supplementary Figure 16). However alternative models, with other mechanisms, for example partial IKKK utilisation by both pathways, could also fit the data. Similarly, the mathematical model was able to recapitulate NF-κB activation dynamics in

response to the alternating TNFα and IL-1β pulses at 50 min interval (Supplementary Figure 17).

Responses to two 5 min pulses of TNFα at different doses were recapitulated by additional consideration of stochastic TNFα binding to its cognate receptor [6,17]. Cells were also stimulated with a TNFα pulse of 0.1 ng ml^{-1} applied at 0 min, and a 10 ng ml^{-1} pulse at of 60 min, Supplementary Figure 18a. While only a fraction of cells responded to the first pulse, all cells responded to the 2nd pulse. When a second pulse in the refractory state was 50 ng ml^{-1} no change in the number of responding cells was observed (in comparison to 10 ng ml^{-1} second pulse, Supplementary Figure 18b).

The model was fitted and validated against the published population level data in mouse embryonic fibroblasts (MEFs), which informed the temporal relationship between NF-κB and IKK activation levels (in particular in the A20 deficient cells) (Supplementary Figure 19-Supplementary Figure 20).

3. Sensitivity analysis of the model

Sensitivity analyses were performed to determine which model parameters controlled different features of the NF-κB response. Latin hypercube sampling showed that the period of NF-κB nuclear oscillations in response to continuous TNFα stimulation was controlled by parameters related to IκBα feedback, while the amplitude of oscillations was in addition controlled by A20 feedback (Supplementary Figure 24). IκBα- and A20-related parameters were also most sensitive in the dynamic sensitivity analysis of the nuclear NF-κB trajectory (Supplementary Figure 25).

Dynamic sensitivity analyses [18] of nuclear NF-κB trajectories in cells that responded to two TNFα pulses showed sensitivity towards IκBα and A20 parameters (e.g., *c1*, *c1a*, *c3a*; Supplementary Figure 26 and 27 for the response to a single pulse). In contrast, the NF-κB dynamics in a non-responding cell was dominated by a slowly decaying dependence on IKKK and A20 parameters (e.g., *IKKKtott*; *c1*, Fig. 4h and Supplementary Figure 26).

Sensitivity analyses of the TNFα and IL-1β receptor modules showed that while related parameters could be relevant (e.g., total number of receptors, *Rtot* for TNFα pulses), the IKKK and A20 levels were more important for single cell refractoriness (Supplementary Figure 28).

4. Model formulation

The IKK signalling module: The IKK module incorporates the multi-step transduction pathway downstream of TNFR and IL1R. The respective pathways are complex and not fully elucidated [19,20], however our data suggested that the refractory period was controlled by molecular events above the IKK and below the receptor level (Fig. 3). The simplest biologically relevant structure that could explain this data was used, effectively reducing this complex network of interaction into a single process (Fig. 4a). In particular, the model assumed previously described interaction between the generic IκB kinase kinase (IKKK) and the A20 feedback protein [6,9,17,21-23] (Fig. 4B). An alternative mathematical description of the TNFα transduction pathway has also been proposed by [3] and included additional molecular events leading in IKKK activation. In agreement with [6,9], we assumed that IKKK exists in three states: 'neutral' (IKKKn) that can be activated by TNFα, 'active' that regulates IKK phosphorylation, and 'inactive' that constitutively converts into a 'neutral' state The total level of IKKK was assumed to be constant per cell, while the A20 inhibited 'active' IKKK, by facilitating the transition to an 'inactive' state and thus limiting the extent of IKK activity. We assumed that TNFR and IL1R transduction pathways utilized different upstream IKKKs ($IKKK_t$ and $IKKK_i$, respectively), which independently converged on the IKK [24]. Similarly, we assumed that A20 inhibited both TNFα- and IL-1β-dependent responses. This effect has been described by a prolonged NF-κB activation in A20 deficient cells in response to TNFα [2-4,16]. Signalling downstream from IL-1β is less characterized; in particular no effect was shown by [2,3] in A20 deficient cells, while a prolonged NF-κB activation was observed by [16] using knockout studies. For simplicity we used a parallel A20-dependent activation mechanism for IL-1β and TNFα transduction pathway.

Distributed IKKK: The distribution of refractory periods was recapitulated in the model by heterogeneous expression of IKKK in the population of cells (Fig. 4d). Specifically, we assumed that the total IKKK level was constant in a single cell but normally distributed in the population with mean $\mu=1x10^6$ and $\mu=1.3x10^6$ for TNFα and IL-1β pathways respectively, and standard deviation $\sigma=0.3\mu$ molecules:

$$f(x,\mu,\sigma) = \frac{1}{\sqrt{2\pi}\sigma} e^{-\frac{(x-\mu)^2}{2\sigma^2}}, x > 0.1 \cdot \mu.$$

When simulating the effect of parameters other than IKKK level (e.g. IKKK recycling rate, m3, and IκBα half life, c4a) on the refractory period, an above normal distribution was used with mean μ_{c4a} equal to its nominal value and μ_{m3} equal to 1.2×nominal value. A standard deviation of $\sigma=0.3\mu$ was used in all cases (as in Fig. 4i). However, other distributions (e.g., log-normal or uniform) could also be used to fit the data (Supplementary Figure 22).

The NF-κB base module: The base module described the core interaction between the NF-κB and the IκBα negative feedback [1,15,25]. Previously, we characterized the role of the IκBε negative feedback in generation of NF-κB oscillation timing in response to the continuous TNFα stimulation [9,26,27]. This mechanism was omitted in the current model and as a result simulated single cell responses to continuous TNFα (and IL-1β) were more synchronous (Supplementary Figure 14).

A20 mRNA and protein level: Previous models assumed that the parameters controlling the NF-κB-dependent IκBα and A20 feedback (rates of transcription, translation, mRNA and protein half-lives) were the same [1,6,9]. This resulted in similar simulated mRNA and protein levels (around 200 mRNA and 100,000 protein molecules) per cell. While quantitative IκBα and A20 protein levels are not yet available, population-level gene expression assays indeed suggested up to 300 IκBα and 600 A20 transcript on average in mouse fibroblast cells [6]. In agreement, recent smFISH measurements of A20 and IκBα transcript abundance in HeLa cells following 10 ng ml^{-1} TNFα stimulation showed heterogeneous

expression patterns with average levels of 250 molecules per cell, respectively [28]. In contrary, our quantitative Nanostring assay suggested that in SK-N-AS cells, the average A20 transcript expression was about 40 times lower than the expression of IκBα following treatments with TNFα and IL-1β (Fig. 6e). Therefore, while not changing any IκBα-related parameters, the A20 transcription rate was decreased accordingly, resulting in approximately 6 mRNA and 3,000 protein molecules of A20 on average. Such relatively low levels might generate substantial intrinsic noise (as shown in in the stochastic formulation of the model, Fig. 5b, c). However, the analysis of equilibrated TNFα stimulation showed that the NF-κB responses were seemingly not affected by this heterogeneity, and instead were deterministic (Fig. 5d). This suggested that the intrinsic noise in A20 regulation has a limited effect on the system's response to TNFα pulses. We expect, that the tight control of the enzymatic activity [16,19,20], rather than apparent heterogeneous expression level drive the function of A20 protein.

Low dose activation due to stochastic receptor binding: In order to recapitulate responses to low doses of TNFα stimulation in SK-N-AS cells (Supplementary Figure 18) stochastic binding of TNFα to its cognate receptor was considered with a fixed number of receptors in each simulation. Here we found that about 70% of C9 cells responded to a 5 min TNFα pulse at 0.1 ng ml^{-1} dose. Previously, using transient transfection with p65-DsRedxp plasmid, we found that about 20% of SK-N-AS cells responded to the same simulation dose [14]. We expect that this difference is due to increased sensitivity of the IκBα-eGFP reporter cells. In the model we assumed that a single cell resides in 1 nl of media. Other volumes could be recapitulated by rescaling receptor-binding rates. The extracellular volume is substantially larger than the volume of a cell (~1 pl on average) therefore the receptor-binding rate would be limited by diffusion of limited number of TNFα molecules in the volume [6,17].

5. Stochastic model description

Stochastic simulation algorithm was used to investigate the role of the intrinsic

noise in the system. A hybrid model was developed in which the biochemical reactions were split into *fast* and *slow*; *fast* reactions were approximated by rate equations, while *slow* reactions were considered stochastic [29]. In particular, these former included the regulation of IκBα and A20 gene activity [9,30] and TNFR receptor binding [6]. In addition, the A20 mRNA death and birth process was considered stochastic due to low number of molecules involved (<10 mRNAs).

We assumed that each gene has two independent homologous copies activated due to NF-κB binding and inactivated by the nuclear IκBα protein [30]. Let $G^i(t) \in \{0,1\}, i = 1,2$ denote the binary state of a promoter of single gene copy: $G^i(t)=1$ whenever NF-κB is bound to the promoter, and $G^i(t)=0$ otherwise. The gene state describes the state of both homologous copies and is given by $G_{A20}(t) = G^1{}_{A20}(t) + G^2{}_{A20}(t)$, and $G_{I\kappa B\alpha}(t) = G^1{}_{I\kappa B\alpha}(t) + G^2{}_{I\kappa B\alpha}(t)$ for *A20* and *IκBα* promoters, respectively. Let the binding propensity r^b be the rate at which the NF-κB binds to its unbound promoter, i.e., if the binding site is unbound then the probability of binding in a short time interval of duration *dt* is $r^b dt$. The dissociation propensity r^d is defined similarly. It is assumed that for NF-κB r^b is proportional to the amount of nuclear NF-κB, while the dissociation propensity r^d is proportional to the amount of nuclear IκBα protein:

$$r^b(t) = q1_x \cdot NF\kappa B_n(t, G),$$

$$r^d(t) = q2 \cdot I\kappa B\alpha_n(t, G),$$

where *G* is the state of the gene, and $q1_x$ (*x={i, a}r* for IκBα and A20 respectively) is the binding rate of NF-κB and q2 is the inducible IκBα dissociation rate. Because the A20 mRNA transcript was detected at later time points than the IκBα mRNA in SK-NA-S cells stimulated with TNFα [1], we assumed different NF-κB binding rates for A20 and IκBα promoters, while dissociation rates were kept constant.

When considering an A20 mRNA birth and death process, let r_t^t and r_t^d be propensities of synthesis and degradation of one A20 mRNA molecule, respectively. Propensity of synthesis if proportional to the transcription rate and the number of active genes, while propensity of degradation is a function of number of A20 mRNA molecules (*tA20*):

$$r_t^t(t) = q_{1t} \cdot G_{A20}(t),$$

$$r_t^d(t) = q_{2t} \cdot tA20(t).$$

Finally, the TNFR receptor-biding propensity, r_r^b is proportional to the number of available TNFα molecules. Similarly, receptor inactivation propensity (due to dissociation of TNFα), r_r^d is proportional to the number of active receptors (R_t):

$$r_r^b(t) = r_1 \cdot TNF(t),$$
$$r_r^d(t) = r_2 \cdot R_t(t).$$

The total propensity function $r(t)$ for the occurrence of any of the reactions is given by

$$r(t) = r_{A20}^b(t) \cdot \left(2 - G_{A20}(t)\right) + r_{I\kappa B\alpha}^b(t) \cdot \left(2 - G_{I\kappa B\alpha}(t)\right) + r_{A20}^d(t) \cdot G_{A20}(t) + r_{I\kappa B\alpha}^d(t) \cdot G_{I\kappa B\alpha}(t) + q_{1t} \cdot G_{A20}(t) + q_{2t} \cdot tA20(t) + r_r^b(t) \cdot (R_{tot} - R_t) + r_2 \cdot R_t(t),$$

where r_{A20} and $r_{I\kappa B\alpha}$ are individual propensities for *A20* and *IκBα*, respectively. The numerical scheme implemented follows that of [30]:

1. At simulation time *t*, for given state of the system calculate the total propensity function *r(t)* of occurrence any of the reactions.
2. Select 2 random numbers p_1 and p_2 from the uniform distribution [0,1].
3. Evaluate the system of ODEs accounting for deterministic reactions until time $t+\tau$, such

$$\log(p1) + \int_t^{t+\tau} r(s)\,ds = 0.$$

4. Determine which reaction occurs at time $t+\tau$ using inequality

$$\sum_{i=1}^{k-1} r_i(t+\tau) < p_2 \cdot r(t+\tau) \le \sum_{i=1}^{k} r_i(t+\tau),$$

where $r_i(t+\tau)$, i=1,..., are individual reaction propensities and *k* is the index of the reaction. Time t+τ is then replaced by τ and the process is repeated.

6. Model simulations

<u>Deterministic model:</u> The system was initialized with the total NF-κB in the complex with IκBα in the cytoplasm, and the total IKK and IKKK was initialized in the respective neutral form (all other state variables are set to zero). The total IKKK level was randomized from the reference distribution per cell, and the

system was simulated to reach the steady state before TNFα (and IL-1β) activation.

<u>Stochastic model:</u> The stochastic receptor binding was considered to recapitulate low dose TNFα simulation. Separately, the intrinsic noise due to activation of A20 and IκBα genes we preformed to show that such model could recapitulate TNFα pulse data at 70 min interval (Fig. 5b). This model, however subsequently failed to agree with the equilibrated TNFα pulse data (Fig. 5c-e), suggesting that extrinsic noise was more important. In the intrinsic noise simulations, cells were simulated for time *t* uniformly distributed on the interval from 2 to 5 hours prior to TNFα stimulation. Due to the natural degradation of IκBα and the resulting basal NF-κB translocations sensitivity to any initial conditions is lost [30].

In the stochastic receptor activation model, reactions in equation 14 (Supplementary Table 3) were replaced by propensities for receptor binding and dissociation, and simulated using the numerical scheme described above. In this description, active receptor number (*Rt*) became a discrete random variable.

In the stochastic transcription model, reactions in Equation 10 (Supplementary Table 3) were replaced by propensities for A20 gene activation and mRNA transcription. Additionally, Equation 7 refers to discrete state of IκBα gene (*GIκBα),* while equation 11 refers to discrete number of A20 mRNA transcript (*tA20*):

7. ddt(tIκBα) = c1a*GIκBα - c3a*tIκBα

11. ddt(A20)= c2*tA20 - c4*A20

The resulting system was simulated using the numerical scheme described above (with all other equation as in Supplementary Table 3).

7. Dynamic sensitivity analysis

Dynamic sensitivity analysis was performed using a method outlined in [18]. Absolute sensitivity equations were calculated by taking the partial derivative of variables X_i, $i=1,..,n$ with respect to the parameter of interest, θ_j

$$\frac{d}{dt}\frac{\partial X}{\partial \theta_j} = \frac{\partial f}{\partial X}\frac{\partial X}{\partial \theta_j} + \frac{\partial f}{\partial \theta_j} = J \cdot S_j + F_j,$$

where $\dot{X} = f(X, \theta, t), X(t_0) = X_0$ describe the set of ODEs, and

$$J = \frac{\partial f}{\partial X} = \begin{bmatrix} \frac{\partial f_1}{\partial x_1} & \cdots & \frac{\partial f_1}{\partial x_n} \\ \vdots & \ddots & \vdots \\ \frac{\partial f_n}{\partial x_1} & \cdots & \frac{\partial f_n}{\partial x_n} \end{bmatrix}, \qquad F_j = \frac{\partial f}{\partial \theta_j} = \begin{bmatrix} \frac{\partial f_1}{\partial \theta_j} \\ \vdots \\ \frac{\partial f_n}{\partial \theta_j} \end{bmatrix} \text{and } S_j = \frac{\partial X}{\partial \theta_j} = \begin{bmatrix} s_{1j} \\ \vdots \\ s_{nj} \end{bmatrix}.$$

The S_j is the column sensitivity vector with respect to *jth* parameter. Both, model variables $X(t)$ and sensitivity scores S_j can be solved simultaneously over time,

$$\dot{X} = f(X, \theta, t), X(t_0) = X_0$$

$$\dot{S_j} = J \cdot S_j + F_j, S_j(t_0) = S_0.$$

Relative sensitivity (RS) scores

$$\bar{s}_{ij} = \frac{\partial x_i / x_i}{\partial \theta_j / \theta_j} = S_j \frac{\theta_j}{x_i}$$

were calculated to allow direct comparisons of temporal model outputs (X_i) with respect to parameter j (for each model parameter). For the total sensitivity analysis of a single variable, RS performance (RSP) along the time scale was calculated

$$RSP_{ij} = \frac{1}{N}\sqrt{\sum_{k=1}^{N} \left|\bar{s}_{ij}(k)\right|^2}$$

where k is the time instance and N is the number of points in the output.

Supplementary References

1 Ashall, L. *et al.* Pulsatile stimulation determines timing and specificity of NF-kappaB-dependent transcription. *Science* **324**, 242-246, (2009).

2 Lee, E. G. *et al.* Failure to regulate TNF-induced NF-kappa B and cell death responses in A20-deficient mice. *Science* **289**, 2350-2354, (2000).

3 Werner, S. L. *et al.* Encoding NF-kappaB temporal control in response to TNF: distinct roles for the negative regulators IkappaBalpha and A20. *Genes & development* **22**, 2093-2101, (2008).

4 Werner, S. L., Barken, D. & Hoffmann, A. Stimulus specificity of gene expression programs determined by temporal control of IKK activity. *Science* **309**, 1857-1861, (2005).

5 Carlotti, F., Dower, S. K. & Qwarnstrom, E. E. Dynamic shuttling of nuclear factor kappa B between the nucleus and cytoplasm as a consequence of inhibitor dissociation. *The Journal of biological chemistry* **275**, 41028-41034, (2000).

6 Tay, S. *et al.* Single-cell NF-kappaB dynamics reveal digital activation and analogue information processing. *Nature* **466**, 267-271, (2010).

7 Warming, S., Costantino, N., Court, D. L., Jenkins, N. A. & Copeland, N. G. Simple and highly efficient BAC recombineering using galK selection. *Nucleic acids research* **33**, e36, (2005).

8 Liu, P., Jenkins, N. A. & Copeland, N. G. A highly efficient recombineering-based method for generating conditional knockout mutations. *Genome research* **13**, 476-484, (2003).

9 Paszek, P. *et al.* Population robustness arising from cellular heterogeneity. *Proc Natl Acad Sci U S A* **107**, 11644-11649, (2010).

10 Shaner, N. C., Steinbach, P. A. & Tsien, R. Y. A guide to choosing fluorescent proteins. *Nature methods* **2**, 905-909, (2005).

11 Day, R. N. & Davidson, M. W. The fluorescent protein palette: tools for cellular imaging. *Chem Soc Rev* **38**, 2887-2921, (2009).

12 Maxfield, F. R. & McGraw, T. E. Endocytic recycling. *Nat Rev Mol Cell Biol* **5**, 121-132, (2004).

13 Kirchhausen, T., Macia, E. & Pelish, H. E. Use of dynasore, the small molecule inhibitor of dynamin, in the regulation of endocytosis. *Method Enzymol* **438**, 77-93, (2008).

14 Turner, D. A. *et al.* Physiological levels of TNFalpha stimulation induce stochastic dynamics of NF-kappaB responses in single living cells. *J Cell Sci* **123**, 2834-2843, (2010).

15 Nelson, D. E. *et al.* Oscillations in NF-kappa B signaling control the dynamics of gene expression. *Science* **306**, 704-708, (2004).

16 Shembade, N., Ma, A. & Harhaj, E. W. Inhibition of NF-kappaB signaling by A20 through disruption of ubiquitin enzyme complexes. *Science* **327**, 1135-1139, (2010).

17 Lipniacki, T., Puszynski, K., Paszek, P., Brasier, A. R. & Kimmel, M. Single TNFalpha trimers mediating NF-kappaB activation: stochastic robustness of NF-kappaB signaling. *BMC bioinformatics* **8**, 376, (2007).

18 Yue, H. *et al.* Insights into the behaviour of systems biology models from dynamic sensitivity and identifiability analysis: a case study of an NF-kappaB signalling pathway. *Molecular bioSystems* **2**, 640-649, (2006).

19 Hayden, M. S. & Ghosh, S. NF-kappaB, the first quarter-century: remarkable progress and outstanding questions. *Genes & development* **26**, 203-234, (2012).

20 Shembade, N., Pujari, R., Harhaj, N. S., Abbott, D. W. & Harhaj, E. W. The kinase IKKalpha inhibits activation of the transcription factor NF-kappaB by phosphorylating the regulatory molecule TAX1BP1. *Nature immunology* **12**, 834-843, (2011).

21 Pekalski, J. *et al.* Spontaneous NF-kappa B Activation by Autocrine TNF alpha Signaling: A Computational Analysis. *PloS one* **8**, (2013).

22 Kellogg, R. A. & Tay, S. Noise Facilitates Transcriptional Control under Dynamic Inputs. *Cell* **160**, 381-392, (2015).

23 Hughey, J. J., Gutschow, M. V., Bajar, B. T. & Covert, M. W. Single-cell variation leads to population invariance in NF-kappaB signaling dynamics. *Molecular biology of the cell* **26**, 583-590, (2015).

24 Oda, K. & Kitano, H. A comprehensive map of the toll-like receptor signaling network. *Mol Syst Biol* **2**, 2006 0015, (2006).

25 Hoffmann, A., Levchenko, A., Scott, M. L. & Baltimore, D. The IkappaB-NF-kappaB signaling module: temporal control and selective gene activation. *Science* **298**, 1241-1245, (2002).

26 Kearns, J. D., Basak, S., Werner, S. L., Huang, C. S. & Hoffmann, A. IkappaBepsilon provides negative feedback to control NF-kappaB oscillations, signaling dynamics, and inflammatory gene expression. *J Cell Biol* **173**, 659-664, (2006).

27 Longo, D. M. *et al.* Dual delayed feedback provides sensitivity and robustness to the NF-kappaB signaling module. *PLoS computational biology* **9**, e1003112, (2013).

28 Lee, R. E., Walker, S. R., Savery, K., Frank, D. A. & Gaudet, S. Fold change of nuclear NF-kappaB determines TNF-induced transcription in single cells. *Mol Cell* **53**, 867-879, (2014).

29 Haseltine, E. L. & Rawlings, J. B. Approximate simulation of coupled fast and slow reactions for stochastic chemical kinetics. *Journal of Chemical Physics* **117**, 6959-6969, (2002).

30 Lipniacki, T., Paszek, P., Brasier, A. R., Luxon, B. A. & Kimmel, M. Stochastic regulation in early immune response. *Biophysical journal* **90**, 725-742, (2006).
